# An Integrated Model to Conduct Multi-Criteria Technology Assessments: The Case of Electric Vehicle Batteries

**DOI:** 10.1021/acs.est.2c04080

**Published:** 2023-03-13

**Authors:** Joris Baars, Felipe Cerdas, Oliver Heidrich

**Affiliations:** † Fraunhofer Institute for Surface Engineering and Thin Films IST, Bienroder Weg 54E, Braunschweig 38108, Germany; ‡ School of Engineering, Newcastle University, Newcastle upon Tyne NE1 7RU, United Kingdom; § Institute of Machine Tools and Production Technologies, Technische Universität Braunschweig, Braunschweig 38106, Germany

**Keywords:** integrated modeling, lithium-ion batteries, life cycle assessment, life cycle costing, criticality assessment, multi-objective optimization

## Abstract

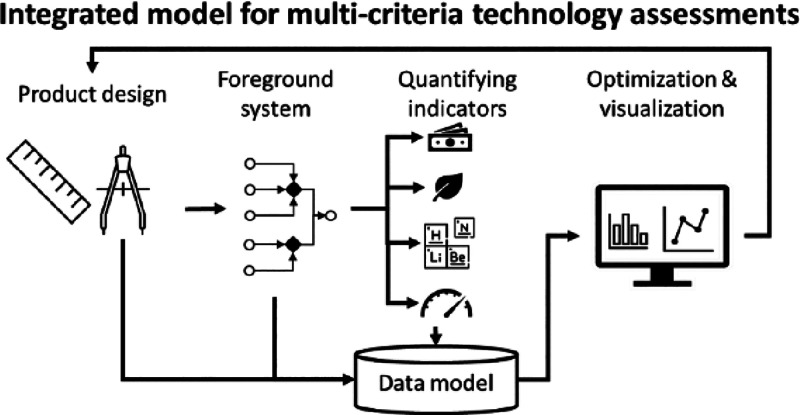

The large-scale adoption of low-carbon technologies can result in trade-offs between technical, socio-economic, and environmental aspects. To assess such trade-offs, discipline-specific models typically used in isolation need to be integrated to support decisions. Integrated modeling approaches, however, usually remain at the conceptual level, and operationalization efforts are lacking. Here, we propose an integrated model and framework to guide the assessment and engineering of technical, socio-economic, and environmental aspects of low-carbon technologies. The framework was tested with a case study of design strategies aimed to improve the material sustainability of electric vehicle batteries. The integrated model assesses the trade-offs between the costs, emissions, material criticality, and energy density of 20,736 unique material design options. The results show clear conflicts between energy density and the other indicators: i.e., energy density is reduced by more than 20% when the costs, emissions, or material criticality objectives are optimized. Finding optimal battery designs that balance between these objectives remains difficult but is essential to establishing a sustainable battery system. The results exemplify how the integrated model can be used as a decision support tool for researchers, companies, and policy makers to optimize low-carbon technology designs from various perspectives.

## Abstract 2

This work presents an integrated model and framework for multi-criteria technology assessments implemented to calculate how material design strategies can improve the cost, carbon footprint, material criticality, and energy density of electric vehicle batteries.

## 1 Introduction

Significant transitions across energy, building, transport, industrial, forestry, and agricultural systems are needed to reach net-zero CO_2_ emissions. To support the development of low-carbon technologies that enable such transitions, substantial amounts of public funding are committed and climate policies adopted.^1−3^ Such technologies, however, can result in technical, socio-economic, and environmental trade-offs. For example, solar photovoltaics (PV) might be one of the most cost-effective and important technologies to reduce greenhouse gas (GHG) emissions^4^ but can increase non-climate-related environmental impacts. Similarly, large-scale offshore wind projects as planned within the next years^5^ result in a significant increase in critical material demand, such as neodymium,^6^ and new challenges related to the treatment of end-of-life carbon fiber turbine blades.^7,8^

Assessing trade-offs of technologies by using analytical models (e.g., life cycle assessment or material flow analysis) guides decision makers in the design and planning of new technologies.^9^ However, mainstream technology assessments and the models they rely on have two main limitations. First, the technical, socio-economic, and environmental indicators of technologies are typically modeled in isolation and addressed by different scientific communities. As a result, trade-offs between sustainability indicators are rarely included in product development processes.^10^ Second, new technologies are subject to high technological variabilities, which requires data calculation and estimations (e.g., process simulation)^11^ and are difficult to capture using mainstream assessment tools (e.g., LCA software). Integrating these into conventional software is time-consuming,^12^ and solving many alternative product configurations is computationally demanding.^13^

To address these two limitations of current technology assessments, different models across scientific disciplines are increasingly integrated.^9,14,15^ This includes, on the one hand, integrations that broaden the impact assessment from a single to several sustainability indicators,^16−18^ and, on the other hand, using engineering calculations and models to establish parameterized models to include technological variability.^15,19−21^ However, there is a need to improve the operationalization of such model integrations to conduct multi-criteria technology assessments. This includes for example practical approaches and guidance on how to integrate different discipline-specific analytical methods,^22,23^ operational frameworks,^24^ computational formulas to determine how data is combined,^25^ and examples to increase the knowledge and practice of integrated assessments.^16,23^

This study presents an integrated model and framework to assess the technical, socio-economic, and environmental aspects of low-carbon technologies, specifically lithium-ion batteries (LIBs) for electric vehicles (EVs). The framework consists of eight procedural steps and combines several analytical models including life cycle assessment (LCA), life cycle costing (LCC), substance flow analysis (SFA), and mathematical optimization. The framework includes general mathematical formulas and a database structure and suggests an open-source software implementation.

## 2 Materials and Methods

**Figure 1:**
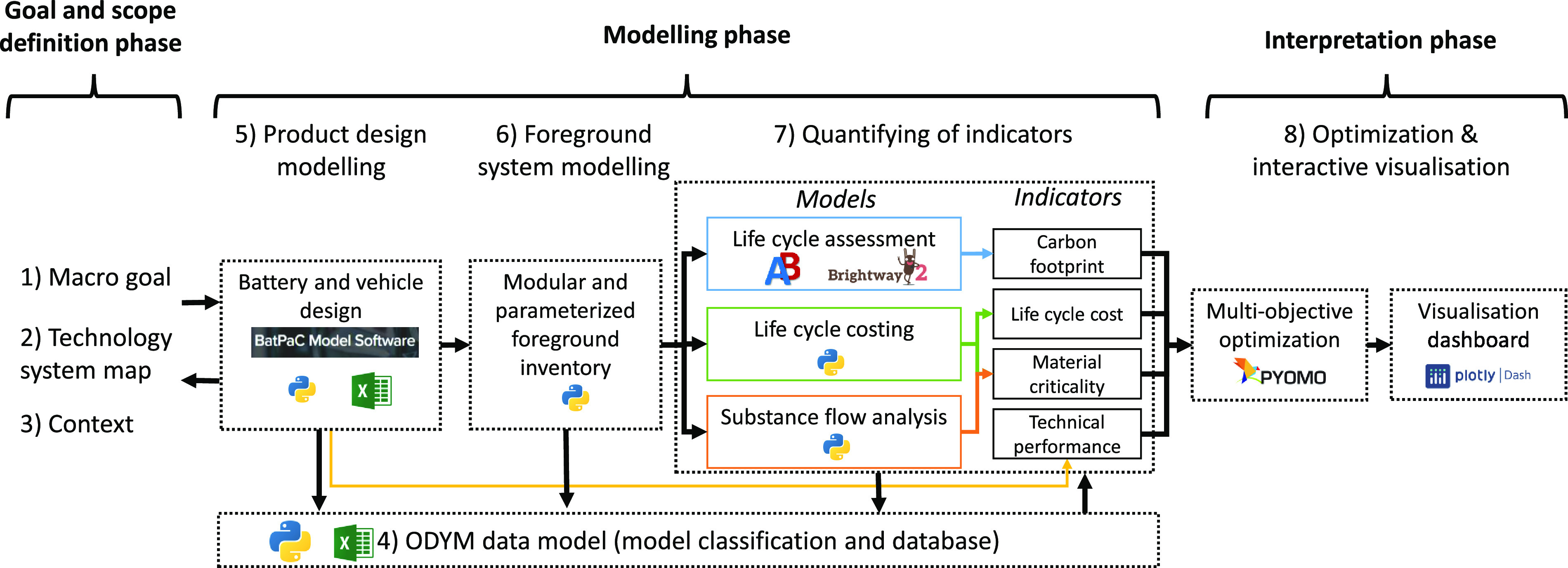
Integrated modeling framework for low-carbon technologies. Each step is exemplified based on the case of LIBs for EVs. The goal and scope definition, modeling, and interpretation phase are based on Heijungs et al.^27^ and steps 1 to 3 are based on Stefanova et al.^28^ The integrated model for batteries is publicly available at the Zenodo repository^29^ as well as the GUI dashboard, available from: https://battery-sustainability-app.herokuapp.com.

The integrated model and framework for low-carbon technology assessments with an implementation for EV LIBs is presented in Figure 1. The framework consists of eight steps, which are divided into three phases based around the basic principles of the life cycle sustainability analysis (LCSA) framework by Guinée et al.:^22^ the goal and scope definition phase, the modeling phase, and the interpretation phase. The original LCSA considers three levels: the product level (micro), the sectorial level (meso), and the economy-wide level (macro). To facilitate the integration of different analytical models of single technologies, we use the LCSA framework here for product level assessments. To improve the operationalization of the LCSA framework, which is considered a key research need,^22,26^ we extend the framework by adding new procedural steps for the modeling phase.

The first three steps in the goal and scope definition phase are based on Stefanova et al.^28^ Steps 4–8 were developed as the integrated modeling part. The integrated model for EV batteries considers many battery designs and calculates four different indicators based on different models. These include carbon footprint (sum of the GHG emissions expressed as kg CO_2_-eq.^30^) calculated with matrix-based LCA,^31^ life cycle cost (sum of the value added per process, expressed in US$) calculated with the computational structure of LCC,^25,32^ material criticality (dimensionless), calculated based on the LCC and matrix-based SFA model,^33,34^ and technical performance (gravimetric energy density (Wh kg^–1^) for the LIB case study) calculated with product design models. Based on these four indicators, multi-objective optimization is used to identify the trade-offs between different perspectives. The model was fully implemented with different open-source software. The model scripts, example Jupyter notebooks, and case study data and an extensive notebook explaining the case study are available from the Zenodo repository.^29^ An additional graphical user interface (GUI) provides access to the model and case study data without the need for programming skills (a public version is available at https://battery-sustainability-app.herokuapp.com). Following is a description of each step of the framework and an example based on the EV LIB case study.

### 2.1 Goal and Scope Definition Phase

#### 2.1.1 Step 1: Macro Goal Definition

The macro goal step identifies the sustainability goals and defines the different objectives that a new technology should address. The global adoption of EVs requires a substantial amount of materials, resulting in new economic, environmental, and supply risk challenges associated with LIB material supply chains.^35^ The macro goal of the case study is therefore to identify optimal designs that aim to improve the sustainability of the battery material system by reducing (1) material cost, (2) material criticality, and (3) carbon footprint and (4) maximizing battery performance.

#### 2.1.2 Step 2: Technology System Map

**Figure 2:**
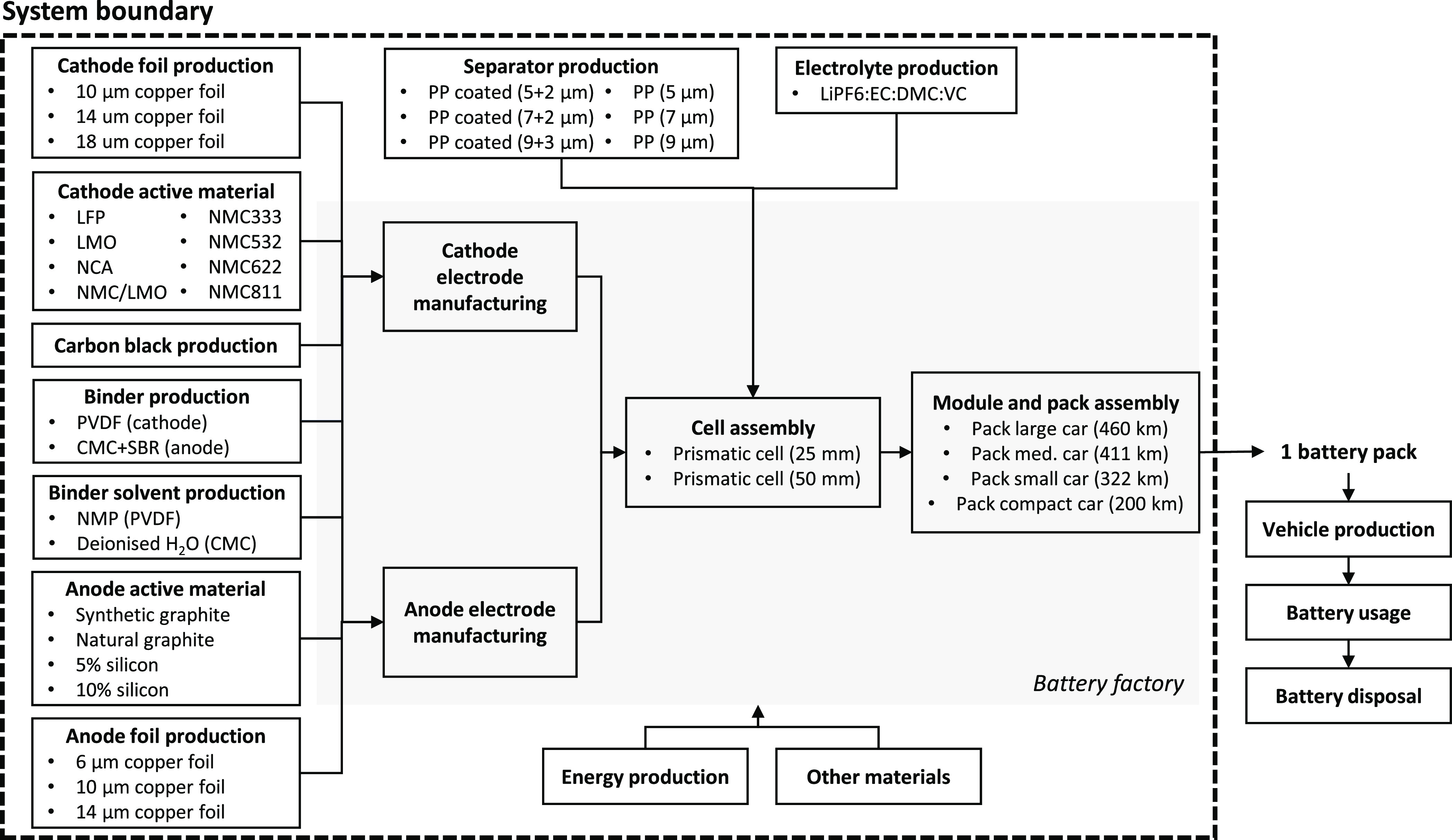
Simplified system boundary of the cradle-to-battery factory gate (gray) representing the most relevant process steps. Technology choices are indicated with the bullet points in different modules (e.g., the anode foil production module can produce three different types of anode foils (6, 10, and 14 μm)) and based on two key sustainable material design strategies: material substitution and dematerializations.^36^ A total of 20,736 different battery designs are included (8 cathode materials × 3 cathode foil types × 6 separator types ... etc.). See SI Section 1 for further details. Abbreviations: LiNi_*x*_Mn_*y*_Co_*z*_O_2_ (NMC_*xyz*_); LiMn_2_O_4_ (LMO); LiFePO_4_ (LFP); LiNi_0.8_Co_0.35_Al_0.05_O_2_ (NCA); polyvinylidene fluoride (PVDF); carboxymethyl cellulose (CMC); styrene butadiene (SBR); ethylene carbonate (EC); dimethyl carbonate (DMC); vinyl carbonate (VC); lithium hexafluorophosphate (LiPF6).

Based on the macro goal definition, the technology system map, a decision tree-like structure, is constructed to identify the relevant technology system and all technology design alternatives. A corresponding technology system map containing all potential battery design choices was established and included a total of 20,736 unique design options (see Figure 2). These design choices were inspired by a range of sustainable material strategies for the product design phase, including dematerialization and material substitution.^36^ While many more battery design choices are possible, the focus of this study is on technologies and sustainable material strategies that can be implemented by battery manufactures in the short term (0–5 years). Novel battery technologies are beyond the scope of this study. Furthermore, material strategies for the use or end-of-life phase are also not included.

#### 2.1.3 Step 3: Context Description

The context description links the goal and scope definition to the modeling phase by identifying the relevant indicators in line with the macro-goal and the empirical models needed to calculate these, as well as the functional unit and system boundary of the study. For the case study, this included (1) battery pack cost calculated using LCC, (2) carbon footprint based on LCA, (3) material criticality based on criticality assessment calculations and SFA, and (4) battery performance based on the pack-level gravimetric energy density calculated with the BatPaC version 5 battery design model^37^ linked to a vehicle energy consumption model.^38,39^ The most commonly used and recommended functional unit for battery LCAs is 1 kWh of storage capacity.^40^ However, as some battery materials do not scale linearly with the storage capacity,^41^ using a capacity-based functional unit would not provide a fair comparison of impacts of different vehicle sizes and the vehicle downsizing as potential mitigation strategy (i.e., large batteries would have a relatively lower impact score compared to smaller batteries due to non-linear scaling of battery pack components such as aluminum for housing). Instead, in this study we used one battery pack for a compact, small, medium, or large EV as functional unit. As the battery capacity in our model is determined by both battery and vehicle design parameters (e.g., cathode active material; drag coefficient) and a fixed desired driving range for different vehicle sizes, we used average driving ranges of current EVs obtained from the EV Database^42^ (see also Section 2.2.2). These include 200, 322, 411, and 460 km for compact, small, medium, and large vehicles, respectively (see also SI Section 2.2). Other functional units, including 1 kWh and 1 kg, can be selected in the online GUI of our integrated model. A cradle-to-gate system boundary is adopted with a temporal scope of 1 year (Figure 2). The LIB factory location is based on the average values of the seven largest potential LIB-producing European countries, Germany, Sweden, Norway, UK, Poland, Hungary, and France,^43^ and has an annual production volume of 500,000 packs (fully utilized). The location and production volume are fully customizable in the GUI. See also SI Section 1 for more info on the goal and scope definition of the case study.

### 2.2 Modeling Phase

The modeling and interpretation phases consist of five steps: establishing of classification and data model, product design modeling, foreground system modeling, calculation of indicators based on different system models, and obtaining of the solution and visualization of the results. Following is a description of each step.

#### 2.2.1 Establish Classification and Data Model

This step establishes a common classification that is used by the data and system model, allowing for an easy alignment of a wide variety of datasets used by the different models (e.g., material prices, process inventories, elemental content). This step is based on the procedures of the Python-based ODYM data model by Pauliuk and Heeren.^44^ The data model in this context can thereby be regarded as the conceptual and logical structure to store and process data.^45^ Although ODYM was initially developed for dynamic material flow analysis, the ODYM data model can, in principle, be used for any type of system modeling. A common model classification is thereby established that is used by both the system and data model. The model classifications refer to a list of items of all dimensions of the system under study (e.g., all chemical elements relevant for the model, all battery factory production processes, all cell materials). By importing a common classification into the software, the datasets and system model are integrated. This makes it easy to align datasets and model developments because both data providers and modelers use the same classification. Such linkage is especially useful when the data provider and modeler are not the same person (e.g., projects within a large consortium or industry collaboration projects).

#### 2.2.2 Product Design Modeling

At the product design modeling step, the bill of materials (BOM) and technical performance data for all design options as identified in the technology system map are obtained. This data is collected using engineering models and calculations to include many different product designs while adhering to technical relations and constraints. The BOM is thereby used as input to the parameterized life cycle inventories (e.g., the weight of aluminum in the cell container of a specific design), and the technical performance is used for technical-related calculations (e.g., energy density of a battery cell).

We utilized the BatPaC battery design model as the underlying product model and obtained the BOM and technical performances. Several adjustments and changes were made to BatPaC to accommodate the included designs choices. Most notably was the inclusion of a vehicle model, used to calculate the energy storage requirements for the different vehicle sizes and include secondary weight savings due to technology choices. The vehicle model is based on Deng et al.^39^ and Kim and Wallington^38^ whereby the required energy storage is calculated based on a desired range and several vehicle specific parameters. A Python script was developed to automate the process of calculating and extracting the BOM and technical performance data from BatPaC based on user-specified design parameters. See SI Section 2 for full details.

#### 2.2.3 Foreground System Modeling

The next step is to model the foreground system, consisting of all energy and material flows. The foreground system modeling step is based on the matrix-based life cycle inventory (LCI) model.^31^ The foreground system is stored as a matrix (the technology matrix and denoted as ***A***′) representing the quantity of material or energy inputs and outputs to produce one unit of that activity (see SI Section 3 for detailed descriptions). Activities directly affected by the product design choices are parameterized whereby input and outputs are based on formulas and linked to the outputs of the product design model. In the case of many alternative product system configurations, the foreground matrix can be modularized to reduce data requirements and improve calculation efficiencies. The result is a non-square matrix where different activities have the same output. Mathematical optimization has been frequently used to solve LCI models with a non-square technology matrix and is further discussed below in the solution step.

The foreground system for the LIB case study consists of all material and energy activity flows that are influenced by the choices of the battery manufacturer (e.g., cathode material choice, cell foil thickness). The material and energy flows within the factory system are parameterized. Material process flows were based on mass balance equations (process output equals process input adjusted for process yield) and product design choices (e.g., cathode material choice) whereby factory process yields were based on BatPaC. Factory energy consumption was based on Degen and Schütte^46^ (cell-producing processes) and Sun et al.^47^ (module and pack assembly). The energy consumption data from both sources was converted from Wh energy per Wh cell/pack to Wh kg^–1^ and multiplied by the design-specific cell and pack weight (with the exception of cell formation) to include the important relation between of energy density and manufacturing energy consumption.^48^

#### 2.2.4 Quantification of Indicators

The next step is to quantify technical, social-economic, and environmental indicators. These are linked to the foreground system and based on different analytical models identified in the context description.

The LCA model (eq 2) is used to calculate the carbon footprint indicator (kg CO_2_ eq. per pack) and refers to the cradle-to-gate GHG life cycle emissions for all foreground production processes. ***h*** is hereby a vector of pre-calculated GHG emissions for each process based on the logic of modular LCA. Using modular LCA, emissions are calculated on the base of single life cycle stages (e.g., 1 kg current collector production) rather than a full life cycle (e.g., 1 battery from cradle to gate). By combining several LCA modules, alternative product life cycle configurations are efficiently modeled at reduced computational effort, which facilitates the integration of mathematical optimization in LCA (see also Steubing et al.^13^). Environmental emissions for each activity in the foreground base matrix are thereby pre-calculated to obtain the emissions of that activity (*h*) using the characterization matrix (***Q***_*h*, *e*_), biosphere matrix (***B***_*e*, *p*_), and scaling vector (***s***_*p*_):^31^
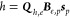
1

***B*** contains the elementary flows (e.g., CO_2_, CH_4_) per activity, and ***Q*** contains the characterization factor to convert the elementary flows to the relevant environmental impact indicator (e.g., global warming potential). The scaling vector (***s***) refers to the desired supply of each activity (*p*) in the background technology matrix (***A***′) to produce one unit of the modularized process product (*g*), captured in the final demand vector,***y*** (e.g., 1 kg of 6 μm copper foil):
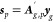
2

The LCA modules were established in the Brightway2^49^ and Activity Browser^50^ software, and ecoinvent 3.7.1^51^ was used as the background database. Most materials in the foreground matrix were not available in the ecoinvent database and were instead modeled based on a wide variety of sources. See SI Section 5 for all LCA inventories, which are also provided in the Zenodo repository.^29^ The ReCiPe 2016^52^ midpoint impact category global warming potential over 100 years (GWP100) was used to express the GHG emissions as a single indicator (kg CO_2_-eq. to air).

The life cycle cost (US$ per pack) was based on the value-added per process and calculated with LCC analysis. Value added in this context refers to the difference between the cost spent on intermediate inputs (e.g., material, energy, or production factors such as labor) and outputs (e.g., waste disposal) and the potential price of the reference product (e.g., battery cell container) of a production activity (e.g., cell production). The sum of the value added thereby equals the total cost of the battery to the customer, the EV producer. The total battery cost was calculated based on two sublayers: production factor and material costs. The production factors (*k*) refer to intermediate inputs other than materials or energy and included direct labor (hours/year), building, land, and utilities (m^2^) and capital (US dollar). The production factor costs was used to obtain the value added for the processes inside the battery factory (e.g., mixing, electrode coating).

The factor requirements for all battery production activities (bp) are represented by the factor requirement matrix (***F***)^53^ and multiplied by the factor cost (**π**) to obtain the value added for all battery production processes (bp):
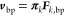
3

The factor requirement matrix was calculated based on Nelson et al.^54^ who use the exponential method (a cost scaling method based on a known cost, production capacity, and an equipment specific exponential^55^) and was scaled to different factory output volumes. Input parameters to calculate ***F*** were thereby directly obtained from BatPaC version 5, and a default factory output of 500,000 packs per year was used. The material cost was calculated with the general computational structure of environmental LCC^25,32^ to obtain the value added of the activities external to the battery factory (ep). The foreground technology matrix was thereby multiplied by the material and energy price vector (**α**):
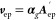
4

Current prices for production factors, materials, and energy were based on a variety of sources as elaborated in Section 6 of the SI. An additional unit cost price was included for several products based on the calculations and data from BatPaC. Overhead costs (e.g., research and development or warranty costs) were allocated to both the factor and material costs based on the basic-to-overhead multipliers as used in BatPaC. Multipliers were thereby calculated from the percentage used to determine the overhead cost, such as profits (e.g., 5% from total investment cost) or depreciation (e.g., 10% of capital equipment and 5% of floor space); see also p. 122 of Knehr et al.^37^

Material criticality is typically defined as the relation between (1) the supply disruption probability related to short-term socio-economic aspects and (2) the vulnerability to such disruption.^56^ In this study, the criticality indicator is based on the product level criticality calculation as described by Lütkehaus et al.^57^ by multiplying the supply disruption probability of a product system with its vulnerability score. The supply disruption probability is thereby based on the demand for materials, expressed as kg of element *e*, with a supply risk characterization factor. For the LIB case study, the ESSENZ characterization factors useful for global supply chains were used, as recommended by the Life Cycle Initiative.^58,59^ The second part of the criticality indicator, vulnerability to supply disruption, is based on the economic vulnerability score of a product system to price hikes of a certain material. Lütkehaus et al.^57^ calculated this as the cost of a material as a fraction of the total product life cycle cost. Following this, the only parameter that is still required for the criticality indicator is the flows of materials, calculated using the matrix-based SFA model formulation. The substance flow matrix (eq 1) represents all the substance flows linked to the foreground system:^33,34^
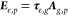
5

where **τ** refers to the transmission matrix, representing the quantity of substance *e* in battery component *g*, and ***A***′ to the foreground matrix, representing the material and energy input and outputs (*g*) per process (*p*), as explained above. The elemental compositions, including Li, Co, Ni, Al, Cu, P, Fe, Si, and natural graphite, of all battery components are included with the exception of the electronic parts (i.e., module electronics and battery management system) due to the difficulty of estimating the elemental composition of these items from modeled batteries in BatPaC. The final indicator, energy density, was calculated with the product model (see SI Section 2). The LCA, LCC, and SFA can also be used as stand-alone models, and several examples are provided in the Zenodo repository.^29^

### 2.3 Interpretation Phase: Multi-Objective Optimization and Interactive Visualization

Following the quantification of the satellite accounts, the next step is to run the model, identify trade-offs between the different design strategies, and visualize them. Multi-objective optimization (MOO) is proposed as a method to solve the model due to its usefulness for ex-ante planning problems when a large set of alternatives are present.^60,61^ A Pareto front is thereby created, highlighting trade-offs between objectives and technology choices.

We constructed a simple MOO model for the LIB case study to identify the Pareto optimal battery design choices. The general matrix-based LCI model was reformulated as an optimization model consisting of four objective functions and two constraints (eqs 6–12). The objective functions (eqs 6–9) include minimizing cost, carbon footprint, and criticality and maximizing energy density and were obtained by multiplying the output of the LCA, LCC, and SFA with the process scaling factor, ***x***. The first term of the criticality score (eq 8) refers to the supply disruption probability and the second term to the vulnerability to such disruption, expressed based on the economic product importance (EPI), i.e., a ratio between elemental specific cost and total product cost. While ratios in optimization models can be solved with fractional programming methods, this results in more complex models. Instead, to keep the case study model simple for illustrative purposes, the EPI for element *e* was simplified by multiplying the battery factory substance flows (*E*) with the substance price, representing the total price of element *e* required for a specific battery design.

Equations 10 and 11 refer to the mass balance equations, whereby material and energy flows must be equal to the final demand vector (***y***) and waste flows can be positive. Finally, eqs 12 to 14 refer to the battery size constraints. The maximum width and height irrespective of the vehicle size are 1200 and 160 mm, respectively. The maximum pack length is based on a battery length to wheelbase ratio of 0.92^62^ and the wheelbase of four representative vehicles, as elaborated in SI Section 2.
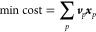
6
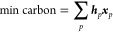
7
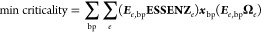
8
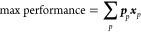
9
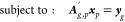
10
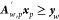
11
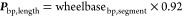
12
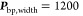
13
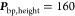
14

The MOO model was established using the open-source Python-based optimization software Pyomo^63^ and solved using the ε-constraint method. The ε-constraint method, one of the most widely used methods to solve MOO, is an a posteriori method where multiple solutions between contrasting objectives are generated and then communicated to the decision maker.^64,65^ This is different from the a priori one (e.g., weighted sum or goal programming), where the preferences of the decision maker are used prior to the optimization to converge to a single solution. The key advantage of a posteriori methods is that different solutions between objectives are generated and can be compared without solving the optimization again with different preferences.^66^ The decision maker is thereby refrained from expressing any preferences prior to the optimization and instead uses the results to decide on the comprise after the optimization.

An additional interactive dashboard was established to access all underlying data and interact with the model. The dashboard allows users to interact with the model by changing battery design (e.g., cathode material type, current collector thickness), process design (e.g., process energy consumption, production location), and impact parameters (e.g., mineral price, material carbon footprints).

## 3 Case Study Results

### 3.1 General Overview of Results

For each of the four vehicle sizes, 5184 different LIB designs were modeled. Of all modeled battery designs, 674 did not fit the vehicle length constraint. This was true only for batteries for large vehicles utilizing lower energy dense cathode materials (LiFePO_4_ (LFP): 324 batteries, LiMn_2_O_4_ (LMO): 268 batteries, and LiNi_0.5_Mn_0.3_Co_0.2_O_2_ (NMC532)-LMO: 82 batteries), which required additional space. A large variance in terms of battery weight and impact results between the different modeled battery designs can be observed (see also Figure SI 8.1 and Figure SI 8.2). For example, the battery weight for large vehicles ranges between 366 and 655 kg while the battery pack cost ranges between $9970 and $16,458 per pack and the carbon footprint between 3837 and 8080 kg CO_2–_eq per pack. The main design parameter to contribute to this variance is the cathode active material choice. LMO and LFP choices achieve the lowest cost, carbon footprint, and material criticality. The resulting energy density of both LFP and LMO batteries, however, is lower (ranging between 110 and 167 Wh kg^–1^ on the pack level) than other cathode materials due to the lower material capacity (mAh g^–1^).

### 3.2 Optimal Material Design Strategies for LIBs

**Figure 3:**
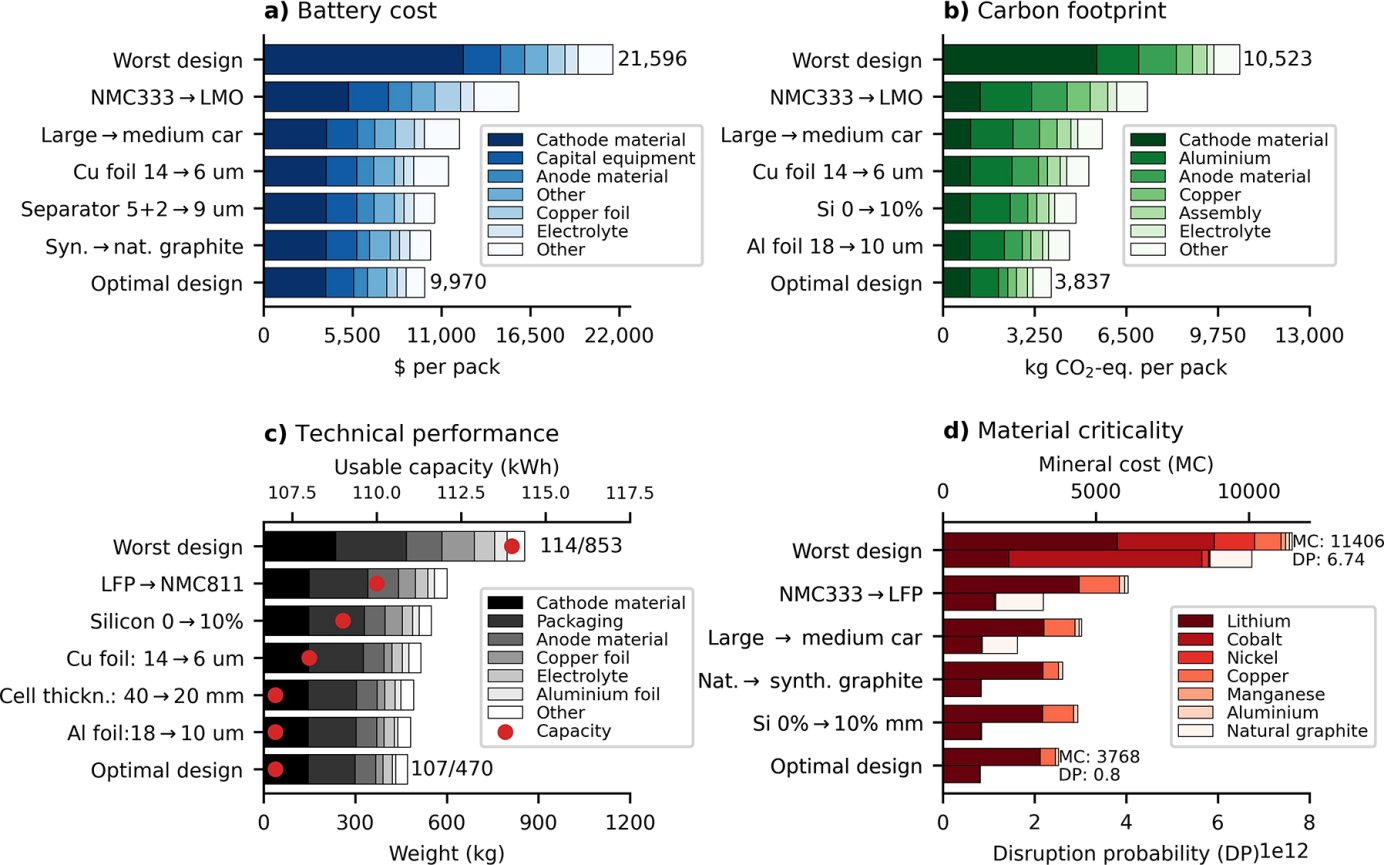
Improvement potential of different material strategies for large vehicles by indicator, including (a) pack-level battery cost in US$ for a factory size of 500,000 packs per year in Europe; (b) pack-level carbon footprint (kg CO_2_-eq.) from cradle to battery factory gate in Europe; (c) gravimetric energy density, defined by the usable pack capacity (kWh_usable_) divided by the weight (kg) indicated with the division operator; (d) pack-level material criticality defined by multiplying the short-term disruption probability potential (DP, dimensionless and based on the ESSENZ characterization factors^58,67^) with the economic vulnerability, expressed as the mineral cost (MC, US$ per element), for each element considered. The figures illustrate the most influential strategies ranging from worst (pack design with the worst score for the indicator) to optimal battery design (pack design with the best score for the indicator). Abbreviations: LiNi_0.3_Mn_0.3_Co_0.3_O_2_ (NMC333); LiMn_2_O_4_ (LMO); LiNi_0.8_Mn_0.1_Co_0.1_O_2_ (NMC811); LiFePO_4_ (LFP); silicon (Si); synthetic (syn.); natural (nat.); mineral cost (MC); disruption probability (DP).

The impacts of the different strategies ranging from the worst to the optimal battery design are shown in Figure 3. To include the vehicle downsizing strategy (i.e., reducing battery size by downsizing to one smaller vehicle size), only the results for the large vehicles are illustrated. Cathode active material substitution and vehicle downsizing are the most promising strategies across all impact categories. Due to the high cost, carbon footprint, and supply risks associated with cobalt, the substitution of NMC333 for LMO and LFP reduces cost, carbon footprint, and material criticality by 27, 32 and 76%, respectively (Figure 3a,b,d). The adoption of lower energy dense LMO and LFP materials however increases the battery weight by ∼150 kg and reduces the energy density. Furthermore, while the total contribution of the cathode active material on the cost, carbon footprint, and criticality is reduced, the increase in battery weight results in a larger contribution of other materials and battery manufacturing-related energy consumption. For example, substituting NMC333 for LMO (Figure 3a,b) results in an increase in materials such as copper (87 to 123 kg), aluminum (48 to 66 kg), separator (3.6 to 5.4 kg), and steel (93 to 101 kg). Aluminum, copper, electrolyte, and manufacturing energy-related CO_2_ emissions therefore increase by 22, 41, 27, and 23%, respectively, while copper and aluminum foil costs increase by ∼47% and separator cost by 50%. In addition, despite the lower Li content in LMO and LFP as opposed to NMC (0.043/0.044 kg of Li per kg of LMO/LFP compared to ∼0.071 kg of Li per kg of NMC), the increase in total cathode material weight (39%) and electrolyte (25%) does not reduce the lithium content in the battery and has thereby a small impact on the criticality score of lithium as compared to substituting cobalt (Figure 3d). Finally, as observed in Figure 3c, strategies to improve the energy density (e.g., substituting LFP for NMC811) reduce both the battery weight (−253 kg) and the usable capacity (−4.75 kWh). The reduction in battery capacity is thereby the result of a lower vehicle weight (reduction of 397 kg) due to lighter battery and secondary weight savings due to a decrease in size of the glider. As a result, the required energy consumption to reach the desired driving range for the large vehicle (460 km) is reduced from 250 to 239 Wh km^–1^, allowing for a smaller battery to reach the same range.

**Figure 4:**
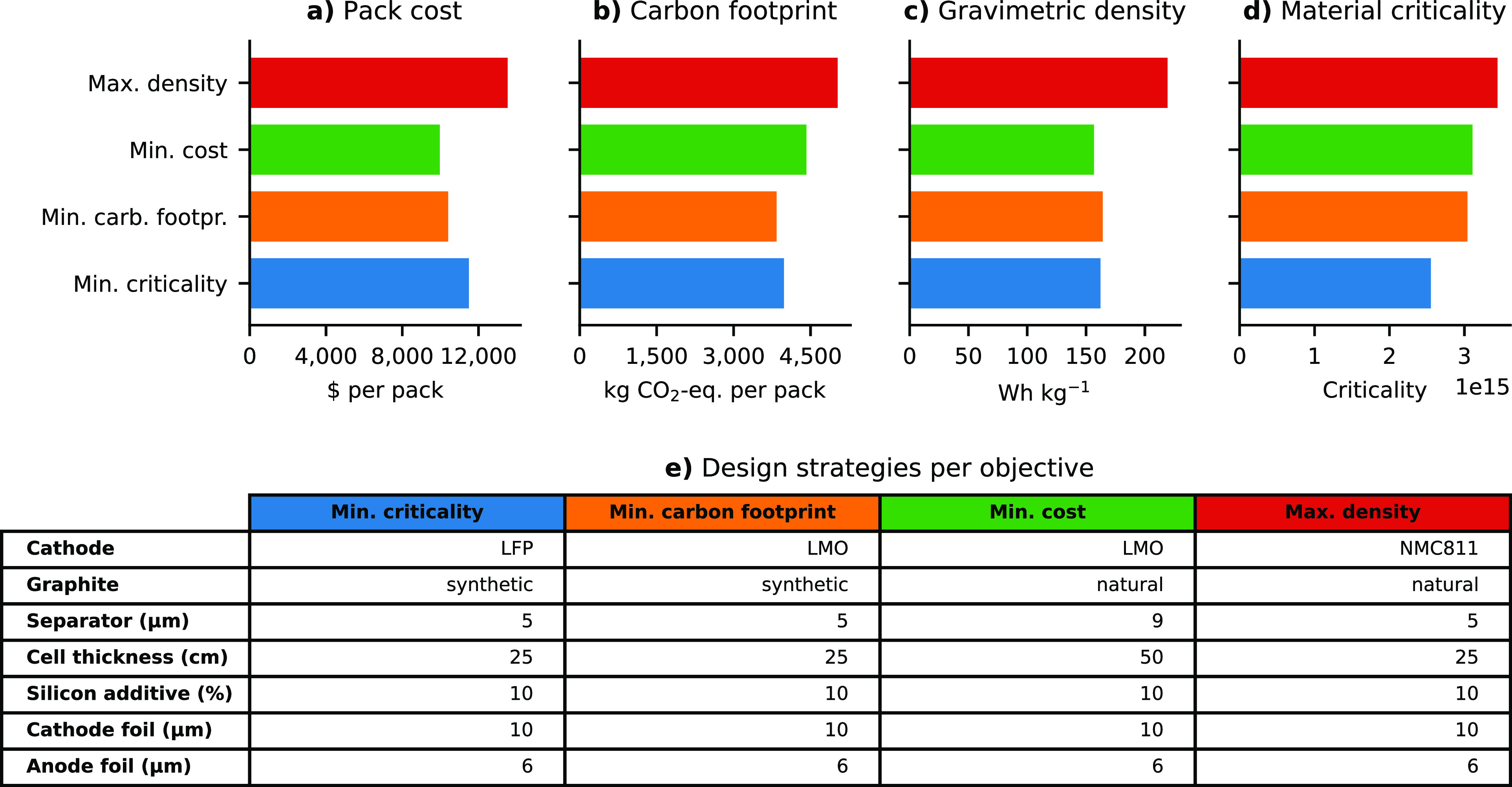
Overview of the single optimization results for a battery in a medium vehicle with a range of 411 km. (a), (b), (c), and (d) refer to the cost, carbon footprint, density, and criticality, respectively, for each objective function. (e) Specific battery design choice for each objective function.

The optimal battery design configuration for a medium vehicle for each objective is presented in Figure 4. The results illustrate a clear conflict between the carbon footprint, cost, and criticality on the one hand and energy density on the other hand. While the impact scores and design choice between the carbon footprint, cost, and criticality are almost identical, the energy density results differ significantly. The main reason is the choice of cathode active material (NMC811 versus LMO and LFP), which results in a smaller and lighter battery (367 kg and 216 L compared to 503–530 kg and 279–327 L for the optimal design of the other objectives) but comes at a higher cost, carbon footprint, and material criticality. Additional trade-offs between objectives include the graphite type (cheaper natural graphite versus lower carbon intensive with less critical synthetic graphite), separator thickness (cheaper 9 μm thickness versus a more expensive but lower impact and a lighter 5 μm separator), and cell thickness (reduces production cost but results in slightly larger and heavier packs).

**Figure 5:**
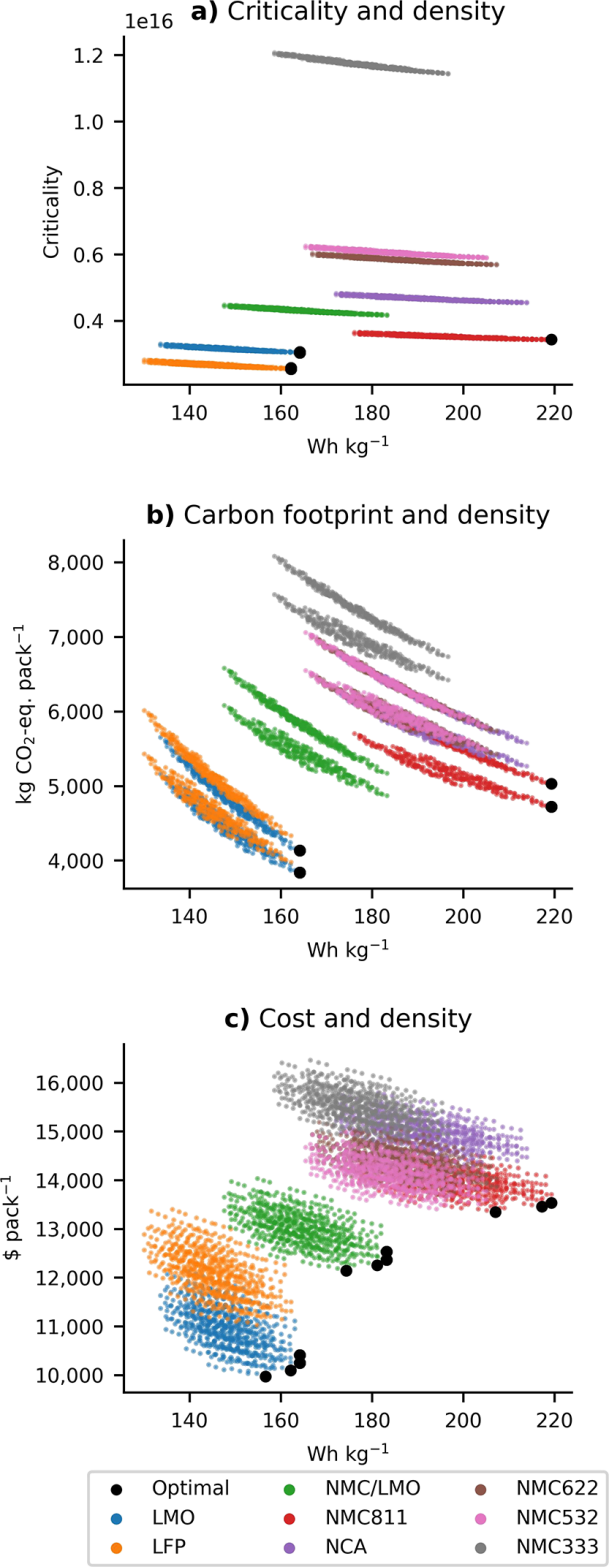
Overview of the Pareto optimal design for all possible battery designs for a medium vehicle with a range of 411 km by cathode active material. The Pareto optimal designs are visualized by the black scatters. All figures illustrate the optimal design between the pack level gravimetric density and (a) material criticality of one pack, (b) pack level carbon footprint, and (c) pack level cost. Abbreviations: LiNi_*x*_Mn_*y*_Co_*z*_O_2_ (NMC); LiNi_0.8_Co_0.35_Al_0.05_O_2_ (NCA); LiFePO_4_ (LFP); LiMn_2_O_4_(LMO).

Following the above observation, MOO using the ε-constraint method was applied to identify several Pareto optimal solutions between the energy density and the other three objectives (Figure 5). The Pareto optimal solution thereby refers to the optimal solution whereby no alternative battery design choice can be identified without making at least one of the objectives worse off (represented by the dark scatters in Figure 5). These points can be used to converge into a decision depending on the compromise that the decision maker is willing to make. As illustrated in Figure 5a,b, finding optimal design choices between the energy density, criticality, and carbon footprint objectives is challenging due to the large discrepancy between the criticality and carbon footprint of the different cathode materials. For example, no favorable material choice can be found between LMO or LFP and NMC811 that has a higher energy density than LMO or LFP but a lower carbon footprint than NMC811 (Figure 5b). This is different from the MOO results of the cost and density (Figure 5c), whereby several Pareto optimal designs are possible. For instance, combining NMC532 with LMO improves the density of batteries as compared to designs with LFP or LMO, while being cheaper than the more energy dense NMC811.

### 3.3 Comparison and Robustness of the Results

**Figure 6:**
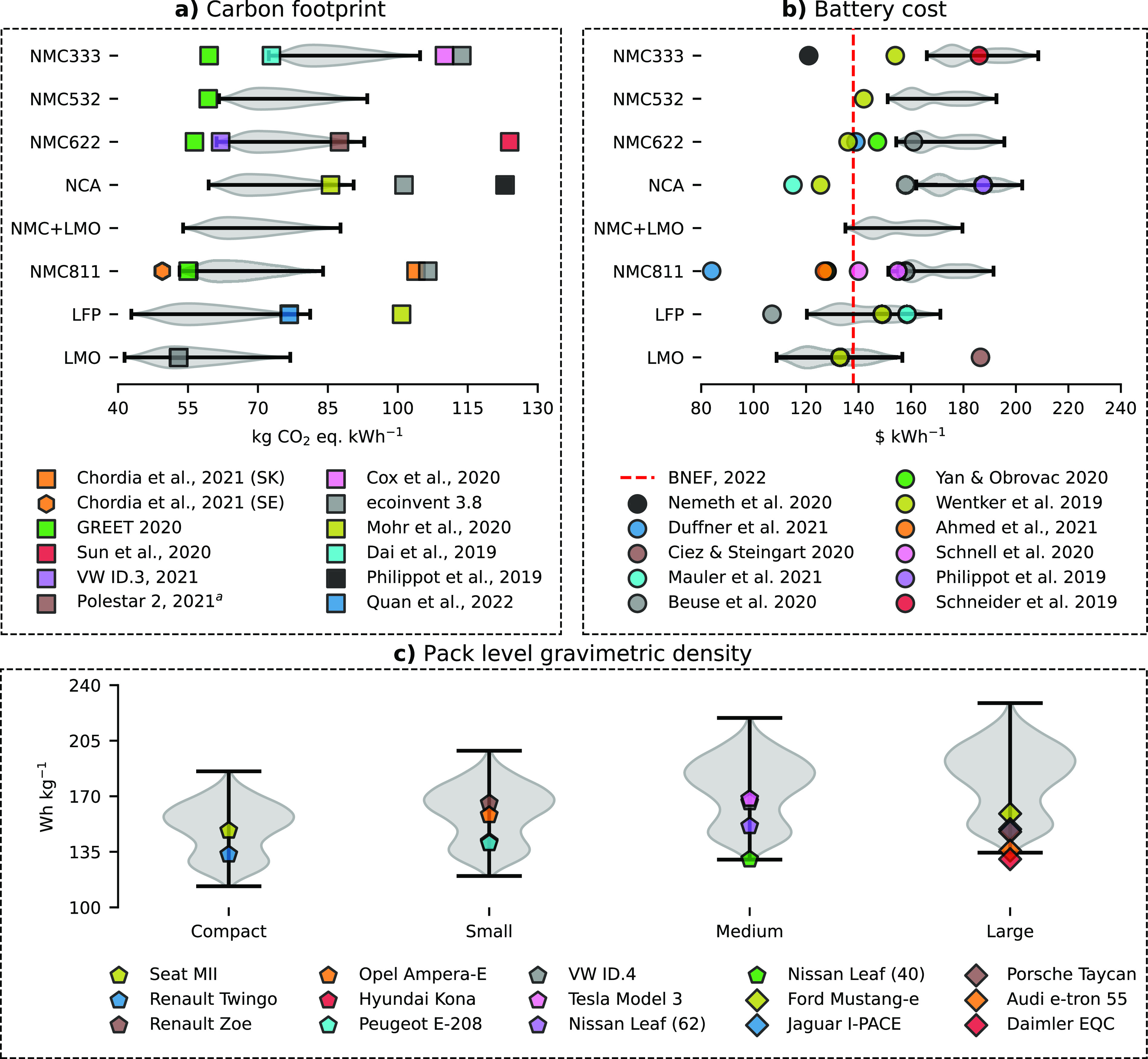
Comparison of the modeled carbon footprint (a) and battery cost (b) results by cathode chemistry with literature values based on a functional unit of 1 kWh. (c) Compares the modeled gravimetric energy density with real values by vehicle sizes from 2021 vehicles as reported in EPA test reports.^70^ Size classification is based on EV Database.^42^

The modeled results are compared with literature data and real values in Figure 6. The carbon footprint results of this study are within the lower to mid-range of recent literature values (Figure 6a). One key factor explaining the difference is the battery manufacturing energy consumption used. For example, in this study, energy consumption ranges between 12 and 27 Wh Wh^–1^ as compared to 41 Wh Wh^–1^ reported by Degen and Schütte^46^ or 34–47 Wh Wh^–1^ reported by Dai et al.^68^ The main reason is that the values of this studies were based on Degen and Schütte^46^ but converted from energy consumption per energy cell (45.5 Wh Wh^–1^) to energy consumption per cell mass (5.1 Wh kg^–1^). As Degen and Schütte^46^ used a relatively low cell energy density (123 Wh kg^–1^) as compared to this study (ranging between 200 and 375 Wh kg^–1^), the energy consumption in this study is considerably lower than the former. The energy consumption per cell mass by Degen and Schütte,^46^ however, is in the range of the reported real-world giga scale energy consumption values by Sun et al.^47^ (5.5 Wh kg^–1^, for 40 GWh factory) and Dai et al.^68^ (9.3 Wh kg^–1^ for a smaller 2 GWh factory). Increasing the battery energy consumption in the model by a factor of 5 (see Figure SI 8.3) increases the carbon footprint between 13 and 35% (see also Figure SI 7.1). This also makes the results more sensitive to the battery production location. For example, high energy consumption and battery cell manufacturing in Poland would increase the carbon footprint between 24 and 60% (see also Figure SI 8.4) depending on the energy density of the battery pack.

Figure 6b highlights that the battery cost results are at the higher end compared to the literature and the average EV LIB industry price of 138 $ kWh^–1^, as reported by BNEF^69^ (red dotted line). The BNEF price in Figure 6b refers to the global average, but a large regional price difference exists with prices for US and Europe 24 and 33% higher, respectively, as compared to China (127 $ kWh^–1^).^69^ Finally, Figure 6c shows the comparison between the modeled energy density with data from several currently available EV models obtained from EPA reports.^70^ As illustrated, most vehicle models fit within the performance results. An additional validation of the vehicle model is presented in SI Figure 8.5 by comparing modeled vehicle weight and electricity consumption (Wh km^–1^) with real values.^71^

A one-by-one parameter sensitivity analysis of the carbon footprint and cost is further presented in SI Section 7.1. The reader is also encouraged to visit the interactive dashboard to further examine the impact of varying parameters (e.g., metal prices or battery production location).

## 4 Discussion and Outlook

This study aimed to improve the operationalization of integrated multi-criteria technology assessment. The LIB case study results demonstrate the usefulness of the framework to inform technology design-related decision-making processes from multiple perspectives. While the results of optimized LIB designs from a cost, carbon footprint, and material criticality point of view are similar, a clear trade-off was found when optimizing solely for technical performance (gravimetric energy density) due to the choice of cathode active material (LMO and LFP versus NMC811). Despite a reduction in battery weight when optimizing performance, the higher cost, criticality, and carbon footprint related to nickel and cobalt makes NMC811 a less favorable active material choice. However, due to the large discrepancy in energy density between LMO and LFP on the one hand and NMC811 on the other, finding Pareto optimal design configurations is challenging.

We identify two key strategies to develop high energy density batteries at a lower cost, carbon footprint, and material criticality. The first strategy is to blend different cathode active materials (e.g., LMO + NMC532 as in the case of Pareto optimal designs when optimizing for cost and density). While blending LMO, NMC, and LFP materials are already found in EV applications to some regard, different options have been proven to be a promising approach for future LIBs.^72,73^ Our model and framework can examine electrodes with multiple types of active materials and identify optimal blends from multiple perspectives at once.

The second strategy is light weighting of structural components on the battery and vehicle level. For example, reducing the anode current collector foil from 10 μm to a thinner, but more expensive, 6 μm copper foil reduces the copper demand for a medium vehicle (81 kWh battery) by 13 kg, improving the carbon footprint, material criticality, and energy density and thereby offsetting the higher price of the thinner foil. Similarly, light weighting structural components is an important lever to improve the energy density of LMO- and LFP-containing batteries. By linking to existing product design models, our framework can be used to explore many more light weighting strategies, e.g., cell-to-pack or cell-to-vehicle concepts,^74,75^ or on the vehicle level, e.g., substitution of high-strength steel for aluminum car bodies and its impact on battery design choice.^76^

Our integrated model serves as a base for future multi-criteria technology assessments to explore novel and emerging battery technologies (e.g., all solid-state batteries, cell-to-pack and cell-to-frame designs). There are several opportunities to improve the model further. First, the currently simplified battery manufacturing energy consumption calculations can be replaced by more sophisticated process models to provide a more accurate energy consumption estimate and explore the impact of more detailed process design parameters (e.g., the relation between cell design and manufacturing energy consumption^77,78^ or simulating different dry room design scenarios^79^). Second, more detailed models for the material supply chain can be added, such as parameterized or regionalized LCI models of battery material extraction^20,80,81^ or included secondary supply chains.^82^ Third, the BatPaC battery design model can be linked to an electro-chemical model to enhance the technical resolution and explore additional design options and constraints such as battery cycle life.^83^ Fourth, time-adjusted background inventories can be integrated to explore prospective scenarios such as the impact of future penetration rates of renewable energies^84^ or future supply scenarios for raw materials (e.g., secondary market share).^85^ Fifth, uncertainty analysis can be integrated in the linear programming formulation to understand how uncertainty impacts the technology choices.^86,87^ In addition, more case studies beyond batteries are needed to illustrate the use of the presented integrated model and framework. This should also include additional indicators currently not considered. Other environmental impact categories, material criticality indicators, performance, or economic impact indicators can be easily added. Social impact indicators can also be further included by aligning social LCA computational models such as described by Thies et al.,^80^ to the current model formulation.

Ultimately, trade-offs and intended and unintended consequences caused by the rapid transition and adoption of low-carbon technologies need to be considered by governments before proposing strategies, regulations, or legislation. For example, battery carbon footprint declarations, secondary mineral requirements, or domestic production incentives as announced in Europe and the USA^88,89^ will have profound impacts as to how batteries are designed, manufactured, and managed at their end of life. Our framework provides an integrated model that is transferable to other technologies. This helps decision makers to understand potential consequences and optimize the adoption of low-carbon technologies across different sectors and geographies.

## Data Availability

The data and code that support the findings of this study are publicly available in the Zenodo repository.^29^

## Supporting Information Available

Detailed description of the goal and scope definition phase (Section 1), modeling phase including case study data (Sections 2–6), and case study results (Sections 7 and 8) (PDF)
