# Supplementary material for: An Integrated Model to Conduct Multi-Criteria Technology Assessments: The Case of Electric Vehicle Batteries
Source: Environ Sci Technol. 2023 Mar 13;57(12):5056–67. doi: 10.1021/acs.est.2c04080 (PMC10061934; doi:10.1021/acs.est.2c04080)
Supplement: Supplementary file 1 — es2c04080_si_001.pdf [file es2c04080_si_001.pdf]

An integrated model to conduct multi-criteria technology assessments: the case of electric vehicle batteries

Supporting Information

Joris Baars[1,2*], Felipe Cerdas[1,3], and Oliver Heidrich[2]
[1]Fraunhofer Institute for Surface Engineering and Thin Films IST, 38108, Braunschweig, Germany
[2]School of Engineering, Newcastle University, Newcastle upon Tyne, NE1 7RU, United Kingdom
[3]Institute of Machine Tools and Production Technologies, Technische Universität Braunschweig, 38106, Braunschweig, Germany

[*]Corresponding author: joris.baars@ist.fraunhofer.de

Contents

1 Supporting information goal and scope definition **S1**

1.1 Macro goal S1

1.2 Technology system map S1

1.3 Context description S11

2 Supporting information battery and vehicle design model **S12**

2.1 Battery and vehicle model S12

2.2 Case study parameters S17

3 Supporting information foreground system **S24**

3.1 Energy consumption battery production S24

3.2 Foreground inventories S29

4 Supporting information substance flow analysis **S41**

5 Supporting information life cycle assessment **S42**

5.1 Anode active materials S42

5.2 Cathode materials S43

5.3 Cell materials other S52

5.4 Module materials S59

5.5 Battery pack materials S63

6 Supporting information life cycle costing **S70**

6.1 Factor requirement and costs S70

6.2 Material and energy prices S72

6.3 Material prices - Mass S72

6.4 Material prices - unit S74

6.5 Energy prices S75

6.6 Calculation cathode active material S76

7 Sensitivity analysis **S81**

7.1 Sensitivity analysis carbon footprint S81

7.2 Sensitivity analysis cost S83

8 Supporting results figures **S85**

References **S88**

List of Figures

S1 Evolution of European EV weight S3
S2 Overview of vehicle mass and battery capacity S3
S3 Cathode active materials used in 2017 vehicles S4
S4 Technology system map of the lithium-ion battery design case study S10

S1 Workflow of the battery and vehicle design model. S12
S2 Vehicle model and BatPaC interaction S14
S3 Available data electric vehicle models EPA and EV-database S19
S4 EV range for all current vehicles S20
S5 BatPaC pack, module and cell format parameters. S20
S6 Battery height and length results S21
S7 Cell capacity results S22
S8 Pack voltage results S22

S1 Workflow of the foreground material and energy flow model. S24
S2 Overview of foreground system S30

S1 Monthly mineral price between 2000 and 2020 . . . . S80

S1 Sensitivity analysis for carbon footprint of all battery designs S82
S2 Sensitivity analysis for the battery cost S84

S1 Overview of modelled battery and vehicle designs. S85
S2 Overview of modelled impact results. S86
S3 Battery manufacturing energy consumption scenarios S87
S4 Battery manufacturing energy consumption scenarios by country S87
S5 Overview of vehicle electricity consumption S88
S6 Gravimetric and volumetric cell energy density for all designs S88

List of Tables

S1 Sustainable material strategies for lithium-ion batteries S2

S2 Overview of anode active material choice and quantity used S5

S3 Overview of anode and cathode current collector thickness and material S6

S4 Overview of separator film material, coating and thickness in teardowns and literature S8

S5 Prices of different separator types S9

S1 General vehicle parameters used S17

S2 General battery design parameters used S18

S3 Segment specific parameters for the vehicle model. S18

S4 Default battery configurations for each vehicle segment S23

S1 Industry based energy consumption data for battery cell production. S25

S3 Nominal machine power for battery production steps S27

S4 Used energy consumption parameters S28

S5 Overview of the default scrap rates S31

S6 Process inventory for anode active material mixing for 1 battery pack S32

S7 Process inventory for cathode active material mixing for 1 battery pack S33

S8 Process inventory for anode coating and drying of 1 battery pack S34

S9 Process inventory for cathode coating and drying of 1 battery pack S35

S10 Process inventory for cathode/anode electrode slitting S36

S11 Process inventory for final electrode drying S36

S12 Process inventory for cell stacking S37

S13 Process inventory for cell terminal welding S37

S14 Process inventory for cell enclosing S38

S15 Process inventory for electrolyte filling and cell enclosing S38

S16 Process inventory for cell formation S39

S17 Process inventory for module and battery assembly S40

S1 Elemental composition of battery components S41

S1 Process inventory synthetic graphite S42

S2 Process inventory for anode current collector copper for all thicknesses S43
S3 Process inventory for NMC333, 532, 622 and 811 precursor production S45
S4 Process inventory for NMC active material production and import S46
S5 Process inventory for NCA precursor production S47
S6 Process inventory for NCA active material production and import S48
S7 Comparison of inventories for the production of 1kg $LiMn_2O_2$. S49
S8 Process inventory for LMO production S50
S9 Process inventory for LFP production S51
S10 Process inventory for cathode current collector aluminium for all thicknesses . . . S52
S11 Process inventory of styrene-butadiene-rubber (SBR S53
S12 Process inventory of non-coated PP separator S54
S13 Process inventory for coated separators S55
S14 Process inventory of elecrolyte production S56
S15 Process inventory of cell container production S57
S16 Process inventory of cell terminal (cathode) S58
S17 Process inventory of cell terminal (anode) S58
S18 Process inventory of module container production for liquid cooled system S59
S19 Process inventory of module terminals S60
S20 Process inventory for module row rack S61
S21 Process inventory of module thermal conductors S62
S22 Process inventory of module polymer spacers gas release S63
S23 Process inventory of battery jacket S64
S24 Process inventory for battery management system S65
S25 Process inventory for module interconnect S66
S26 Process inventory for cooling tubes S67
S27 Process inventory for pack heater S68
S28 Process inventory for pack terminals S69

S1 BatPaC and foreground system process mapping S71
S2 Production factor costs S72
S3 Battery material mass prices S73
S4 Battery material unit prices S75
S5 Electricity and gas prices S76
S6 Cathode active material process cost and profit margin calculation S77

S7 Current mineral prices in dollar per kg of pure element S78
S8 Historic low and high real 2020 prices per kg of pure element S78

S1 Parameters and values for senstivity analysis of battery carbon footprint S81
S2 Upper and lower bounds for the sensitivity analysis of the costs S83

List of Abbreviations

BOM Bill of materials

CAM Cathode active material

CMC Carboxymethyl cellulose

EPA Environmental Protection Agency

HWFET Highway Fuel Economy Test

LCO Lithium cobalt oxide

LFP Lithium iron phosphate

LMO Lithium manganese oxide

NCA Lithium nickel cobalt aluminum oxide

NG Natural graphite

NMC Lithium nickel manganese cobalt oxide

PCPM Product costs and profit margins

PVDF Polyvinylidene fluoride

SBR Styrene-Butadiene Rubber

SG Synthetic graphite

SMM Shanghai Metals Market

SOC State of charge

UDDS Urban Dynamometer Driving Schedule

1. Supporting information goal and scope definition

Following is an additional description of the steps in the goal and scope definition, including macro goal, technology system map and context description (Stefanova *et al.*, 2014).

1.1 Macro goal

The macro goal step identifies the sustainability goals and defines the different objectives that a new technology should address. It is combined with a comprehensive market and policy analysis of the technology to identify potential technology scenarios to improve these trade-offs as outlined in the technology system map (step 2).

The macro goal for the case study can be briefly defined as follow. GHG emissions from the European transport sector have remained stable or increased in the last five decades (Lamb *et al.*, 2021). New policies by European governments have been adopted to rapidly increase the adoption of EVs (IEA, 2021) and large-scale LIB factories are currently planned across the continent (CIC energiGUNE, 2021). The EV transition, however, has significant implications on the raw material supply chains for LIBs. This results in three main material sustainability challenges: 1) the impact of potential material price increases and its negative impact on battery cost (Boer *et al.*, 2021; Mauler *et al.*, 2022), 2) the relatively large environmental impact of raw material extraction and battery production (Aichberger & Jungmeier, 2020; Chordia *et al.*, 2021) and 3) the criticality of key battery materials with high risk supply chains EC (2020*b*). With LIBs increasingly used for automotive applications and the technologies still emerging, improving the sustainability of the material system is an important design objective for LIB and EV manufacturers. For European manufactures this is further enforced by new legislative measures as proposed in the Battery Regulation by the European Commission (EC, 2021), where measures such as target rates for recycled content in new batteries or mandatory carbon footprint declaration are proposed. These measures are thereby likely to become important levers for future LIB designs (Melin *et al.*, 2021).

This case study illustrates how different material design strategies can improve the sustainability of the material system of LIBs by reducing 1) material cost, 2) material criticality 3) carbon footprint and 4) maximizing battery performance.

1.2 Technology system map

Based on the macro goal definition, the technology system map, a decision tree-like structure, is constructed as suggested by Stefanova *et al.* (2014). The purpose of the map is to identify the relevant technology system and all design alternatives. The map represents relations between technology sub-systems through three basic relations: 1) is-a, representing a technology system of a generic type (e.g. different cathode active material types); 2) part-of, relations between technology systems decomposing the technology system into different sub-systems (e.g. cathode active material and battery cell); and 3) mutual exclusion, referring to the fact that two technological options cannot be implemented together (e.g. the choice of a solid state battery excludes several conventional LIB production processes).

The technology system map of the case study (Figure S4) includes all LIB design choices that could improve the sustainability of the LIB materials system. We focus on the short time (0-5 years) and only include the two general sustainable material strategies for the product design

phase: dematerialization and material substitution (Olivetti & Cullen, 2018). To construct the relevant technology system map and identify all relevant design choices, LIB designs of current EVs were examined based on teardown reports (A2Mac1, 2021) and literature sources. The identified material design strategies are provided in Table 1. To limit the size of the technology system map, each design strategy contains a few choices. The final technology system map contains 20,736 battery design options.

Following is a more detailed discussion of each LIB design variable and parameter range included in the model.

Table S1: Sustainable material strategies for lithium-ion batteries

**Strategy**	**Design variable**	**Range**	**Unit**
1) Downsizing	Vehicle size	Compact, small, medium, large	Category
2) Substitution	Cathode material	$NMC_{333/532/622/811}$, NCA, LFP, LMO, LMO/NMC	Category
	Graphite type	Synthetic, natural	Category
	Silicon additive	0 - 10	%
3) Foil & film thickness	Cathode foil thickness	10:18	µm
	Anode foil thickness	6:10	µm
	Separator thickness	5:9	µm
4) Form factor	Cell thickness	20, 40	mm

1.2.1 Strategy 1: vehicle downsizing.

Downsizing is here defined as a switch from a large to a small vehicle size. EVs are substantially heaver than ICE vehicles due to the additional weight of the battery. While traditionally compact EV models where electrified, improvements in energy density has resulted in growing battery sizes and vehicle weights (Shaffer *et al.*, 2021). This trend can also be observed when comparing the yearly average weight of all fully electric vehicles registered in Europe compared to the general vehicle fleet (Figure S1).

While vehicle downsizing is a straightforward way to reduce overall battery weight and corresponding carbon emissions (Wolfram *et al.*, 2021; Ellingsen *et al.*, 2016), it has not received much attention yet (Shaffer *et al.*, 2021). The first strategy therefore refers to a reduction in battery size through vehicle downsizing. Based on the currently available EV models as reported on the EV-Database (2021), four major vehicle sizes with varying mass, range and battery capacity were identified. These include mini (A segment), small (B segment), medium (C segment) and large (D, E, F, and, J segments) (Figure S2).[1] These four segments are included as a design parameter for the strategy downsizing (switch to one smaller segment, i.e. large to medium, medium to small, small to compact).

[1]The large (D), executive (E), and luxury (F) segments have been grouped into one, as vehicles in these segments have a comparable weight and size.

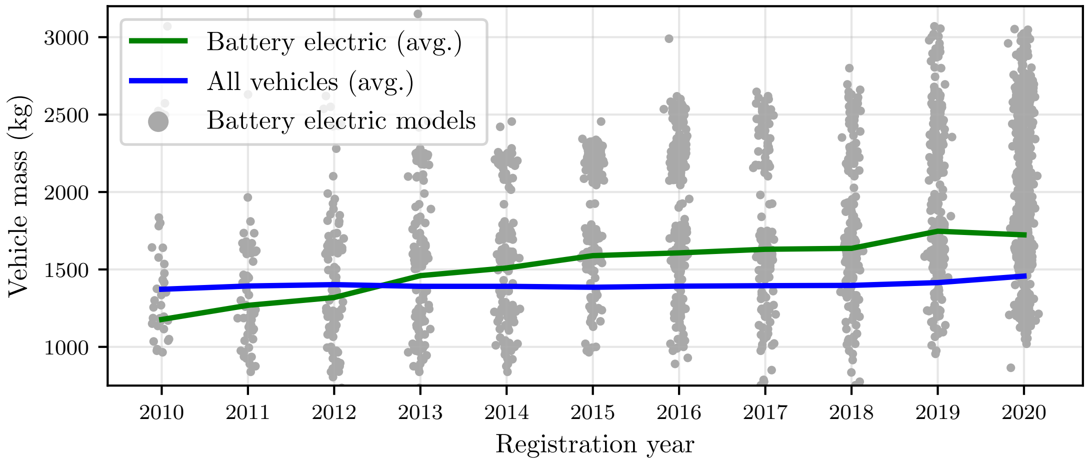

Figure S1: Evolution of European average (avg.) electric vehicle weight compared to the average vehicle fleet. Data is based on all vehicle models obtained from the EEA (2021).

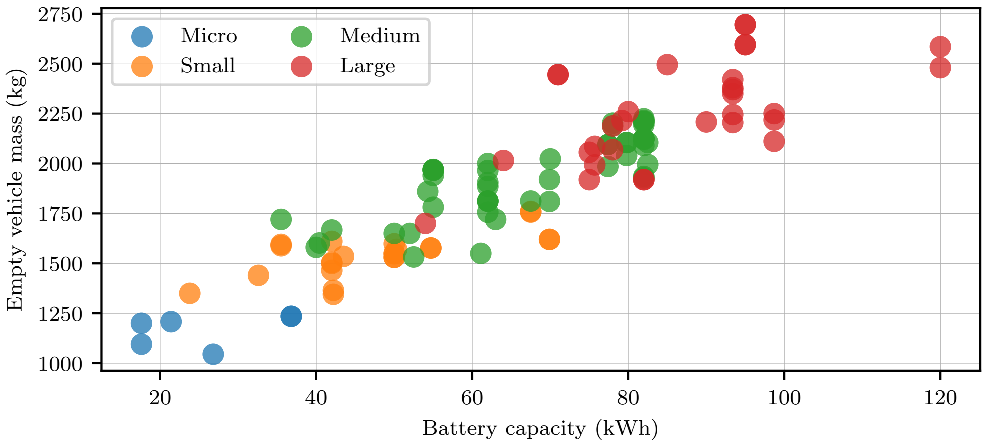

Figure S2: Overview of vehicle mass and battery capacity by segment of all current EV models. Data obtained from EV-Database (2021).

1.2.2 Strategy 2: Cathode material substitution.

The second strategy refers to cathode active material (CAM) substitution. A wide variety of active materials are suitable for EV applications, including $LiNi_xCo_yAl_zO_2$ (NCA), $LiFePO_4$ (LFP), $LiCoCO_2$ (LCO), $LiMn_2O_4$ (LMO) and $LiNi_xMn_yCo_zO_2$ (NMC) where x, y and z come in a variety of blends, most typically 3:3:3, 5:3:2, 6:2:2 and more recently 8:1:1 (Schmuch *et al.*, 2018). Data on CAMs in European EVs registered in 2017 illustrate that different materials are used for EVs (Figure S3).

Large differences exist between CAMs in terms of energy, cost, cyclability, power, safety (Li *et al.*, 2022). LMO, for instance, has a high cycle life, low environmental footprint and cost but a relatively low theoretical capacity (108 mAh g^{-1}) compared to layered structured materials (155-212 mAh g^{-1}). The CAM choice is therefore considered a key material design decisions that impacts the cost, performance (Schmuch *et al.*, 2018), material criticality (Helbig *et al.*, 2018) and carbon footprint (Peters *et al.*, 2017). Based on the underlying design model as will be discussed in Section 2, the following eight alternative CAM decision choices are included

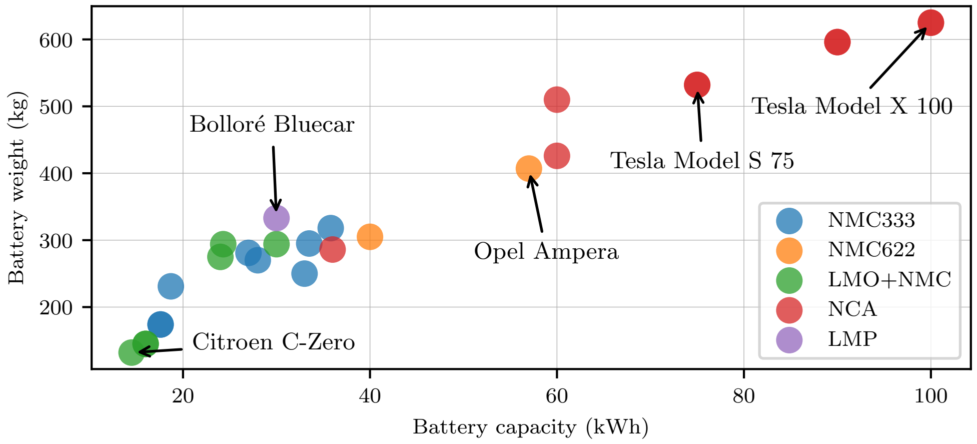

Figure S3: Cathode active materials used in European EV models registered in 2017. Data obtained from Baars *et al.* (2021).

in the case study: NMC-333, NMC-532, NMC-622, NMC-811, NCA, LMO, LMO+NMC532 (consisting 50% of each) and LFP.

1.2.3 Strategy 3: Anode active material substitution (graphite)

Graphite is the most favourable anode active material (AAM) for LIBS due to its relative high theoretical capacity (372 mAh g^{-1}), low cost, good electrical conductivity, and cycle life (Wells, 2018). Two types of graphite are used in EV LIBs: synthetic graphite (SG) and natural graphite (NG) (see Table S2). While SG have higher levels of purity and quality, the energy intensive production phase whereby carbonaceous material, typically pet coke and coal tar pitch, are transformed into graphite using a high-temperature treatment (temperatures above 2800 °C for several days) (Dühnen *et al.*, 2020), has a higher cost compared to NG (Asenbauer *et al.*, 2020; Schmuch *et al.*, 2018). The relative GHG emissions of the SG and NG components of total LIB life cycle emissions are relatively high (e.g. Dai *et al.* (2019*a*) estimate that 7% of the total cradle-to-gate emissions are attributed to SG). GHG emissions of SG and NG are comparable, but highly dependent on the production location and corresponding carbon intensity of the electricity mix (Dolega *et al.*, 2020; Manjong *et al.*, 2021). Synthetic and natural AAM are therefore included as two design choices in the model.

Table S2: Overview of anode active material choice and quantity used. Abbreviations: Natural graphite (NG); synthetic graphite (SG); polyvinylidene difluoride (PVDF); Carboxymethyl cellulose binder (CMC); Styrene-Butadiene Rubber (SBR); Styrene-acrylonitrile copolymer (SAC); polytetrafluoroethylene (PTFE).

**Source**	**Material**	**Quantity (wt%)**
Teardown 2 (A2Mac1, 2021)	NG/CMC/SBR	96.3/2.1/1.6%
Teardown 3 (A2Mac1, 2021)	SG	Not reported
Majeau-Bettez *et al.* (2011*a*)	NG/PTFE	95/5%
Ellingsen *et al.* (2014)	NG/CMC/PAA	96/2/2%
Wentker *et al.* (2019)	SG/PVDF/carbon black	91/5/%
Philippot *et al.* (2019)	NG/SI/SAC	94/2/4%
Dai *et al.* (2019*a*)	SG/PVDF	

1.2.4 Strategy 4: Anode active material substitution (silicon monoxide)

Increasingly, small amounts of Si oxide are added (up to 10 wt%) to the graphite AAM due to its substantially higher theoretical capacity (4200 mAh g^{-1}), appropriate voltage, abundance and non-toxic characteristics (Asenbauer *et al.*, 2020; Armand *et al.*, 2020*a*; Liu *et al.*, 2019). Including small amounts of Si can therefore improve the energy density of cells while lowering cost (Greenwood *et al.*, 2021) and improving the environmental footprint of batteries (Kallitsis *et al.*, 2020). The amount of Si in the anode is expected to increase beyond 10 wt% in the near term (Dühnen *et al.*, 2020), such as the 20 wt% expected by Volkswagen between 2020-2025 (Roskill, 2020). However, the large volume changes of Si during lithiation/de-lithiation (up to 280% versus 12% for graphite (Chen *et al.*, 2017)) results in a rapid deterioration of capacity and cycle life, constraining the further increase of Si content in the anode (Zhang *et al.*, 2020; Liu *et al.*, 2019). Silicon monoxide (SiO_x) is instead used by battery manufacturers due to its lower volume expansion (roughly 200%) and ease of manufacturing (Greenwood *et al.*, 2021). Nanostructured Si could further reduce the mechanical fractures during de-lithiation (Zhang *et al.*, 2020; Ball *et al.*, 2020), but is not commercialised yet. Substitution of graphite for SiO_x in the AAM is therefore the fourth included strategy. The quantity choice of SiO_x is restricted to 10 wt% and can be both mixed with synthetic and natural graphite.

1.2.5 Strategy 5: current collector foil thickness.

Reducing the foil thickness of the current collectors is considered an important strategy to increase the volumetric (L kWh^{-1}) and gravimetric (Wh kg $^{-1}$) energy density, reduce costs (Chu & Tuan, 2017; Zhu *et al.*, 2021) and improve the carbon footprint (Chordia *et al.*, 2021).[2] Current collectors consist of thin metal foils, whereby Al is typically used on the cathode side and Cu on the anode side (see Table S3). The anode and cathode collectors contribute roughly to 13% and 5%, respectively, of the total cell weight (Zhu *et al.*, 2021), and result in a considerable

[2]Chordia *et al.* (2021) estimates that 14% (3.37 CO_2-eq for aluminium and 3.13 CO_2-eq for Cu) of the total GHG emissions of batteries are attributed to the current collectors.

amount of Cu and Al for a total EV pack (Dai *et al.*, 2019*a*).

Table S3: Overview of anode and cathode current collector thickness and material. Teardown refer to state-of-the-art batteries obtained from commercial benchmark reports of automotive batteries (A2Mac1, 2021).

	**Anode**		**Cathode**	
**Source**	Thickness (µm)	material	Thickness (µm)	material
Teardown 1	8	Cu	16	Al
Teardown 2	8	Cu	17	Al
Teardown 3	8	Cu	13	Al
Teardown 4	11	Cu	20	Al
Majeau-Bettez *et al.* (2011*a*)	15-20	Cu	15-20	Al
Ellingsen *et al.* (2014)	10-30	Cu	10-30	Al
Wentker *et al.* (2019)	10	Cu	20	Al
Wood *et al.* (2015)	9	Cu	15	Al
Schnell *et al.* (2020)	10	Cu	15	Al
Sony VTC6A (Lain *et al.*, 2019)	10	Cu	12	Al
Sony VTC5A (Lain *et al.*, 2019)	14	Cu	15	Al
A123 M1A (Lain *et al.*, 2019)	10	Cu	19	Al
LG HB2 (Lain *et al.*, 2019)	15	Cu	27	Al
LG HB4 (Lain *et al.*, 2019)	15	Cu	25	Al
LG HG2 (Lain *et al.*, 2019)	10	Cu	10	Al
Samsung 25R (Lain *et al.*, 2019)	10	Cu	14	Al
Samsung 30Q (Lain *et al.*, 2019)	10	Cu	14	Al
Samsung 48G (Lain *et al.*, 2019)	10	Cu	12	Al
VW ID.3 (Günter & Wassiliadis, 2022)	12	Cu	14	Al

Driven by progress in LIB manufacturing (Nelson *et al.*, 2019), thinner Al and Cu foils have been used over the past 25 years (Zhu *et al.*, 2021). In 1998, the Al cathode foil of an average LIB cells had a thickness of 25 µm and a Cu anode foil had a thickness of of 18 µm (Zhu *et al.*, 2021). This currently ranges from 13 to 20 m for Al and 8 to 11 µm for Cu foils (Table S3), although 6 µm Cu foil thickness used in EV LIBs have been reported (McKinsey, 2021). This, however, is considered the minimum thickness due to limitations in the current production processes (rolling annealing and electrode-position process) for LIB Cu foils (Chu & Tuan, 2017), as well as the lower power density as a result of the decrease in electrical conductivity and heat transfer properties of thinner current collectors (Zhu *et al.*, 2021). Using thin current collectors is therefore an important strategy to improve the performance of batteries, but comes at a higher financial cost. For example, the treatment charge[3], for a 6 µm LIB current collector is 7.28 $/kg, whereas a 8 µm LIB current collectors is 5.72 $/kg (SMM, 2022). Cu nanowire foils as thin as 1.5 µm are reported in the literature (Chu & Tuan, 2017), but currently not commercially available.

Based on the identified thickness for Al and Cu foils currently used in EV LIB cells, a parameter range of 10 to 18 µm for Al and 6 to 14 µm for Cu current collectors are included as design strategies.

[3] i.e. fees paid to convert copper material into foil.

1.2.6 Strategy 6: Separator film thickness

Within common LIB cells, separators account for roughly 13% of the total material costs (Duffner *et al.*, 2020*b*). The separator typically consists of a microporous membrane of polyolefins separating the cathode and anode from physical contact (Crenna *et al.*, 2021; Schmuch *et al.*, 2018; Zhu *et al.*, 2021). A ceramic coating (3-5 µm) can be applied on one or both sides of the separator to reduce the shrinkage of the polyolefin materials at higher temperatures (Yang *et al.*, 2017; Kwade *et al.*, 2018). Table S4 provides an overview of separator material, coating layers and thickness based on teardown reports and literature values. Separator materials typically consist of a single layer of PE or PP, or a combination of the two.

Table S4: Overview of separator film material, coating and thickness in teardowns and literature. Teardown refer to state-of-the-art batteries obtained from commercial teardown reports (A2Mac1, 2021).

**Source**	**Foil material**	**Thickness film (µm)**	**Coating**	**Thickness coating (µm)**
Teardown 1	PE	10	Al_20_3/pvdf	6
Teardown 2	PE/PP/PE	18 (7, 6, 5)	AlOOH	6
Teardown 3	PE	8	Ceramic	8
Teardown 4	PP	16	Ceramic	6
Dai *et al.* (2019*a*)	PE/PP (20:80)	-	-	-
Majeau-Bettez *et al.* (2011*a*)	PE/PP (50:50)	-	-	-
Ellingsen *et al.* (2014)	PP	-	-	-
Greenwood *et al.* (2021)	PE	9	Al_20_3/CMC/ acrylic	3
Wood *et al.* (2015)	PE	25	-	-
Schnell *et al.* (2020)	polyolefin	20	-	-
Zackrisson *et al.* (2010)	PE/PP(50:50)	-	-	-
Sun *et al.* (2020)	PE	-	-	-
Philippot *et al.* (2019)	PE	-	Ceramic	-
ecoinvent/Notter *et al.* (2010)	PE	-	Silica	-
Sony VTC6A (Lain *et al.*, 2019)	-	7	-	-
Sony VTC5A (Lain *et al.*, 2019)	-	8	-	-
A123 M1A (Lain *et al.*, 2019)	-	18	-	-
LG HB2 (Lain *et al.*, 2019)	-	10	-	-
LG HB4 (Lain *et al.*, 2019)	-	10	-	-
LG HG2 (Lain *et al.*, 2019)	-	10	-	-
Samsung 25R (Lain *et al.*, 2019)	-	10	-	-
Samsung 30Q (Lain *et al.*, 2019)	-	8	-	-
Samsung 48G (Lain *et al.*, 2019)	-	8	-	-
VW ID.3 (Günter & Wassiliadis, 2022)	-	18	-	-

As illustrated by Crenna *et al.* (2021), the choice of polyolefin material brings almost no changes in the environmental burden of all 16 impact categories of the Environmental Footprint method (Zampori & Pant, 2019). However, with varying prices for separators based on film thickness (see Table S5) and separators being a large cost factor for LIB cells (Wentker *et al.*, 2019), varying the separator thickness might impact the LIB cost. Different separator thickness choices are therefore included as design strategy. The separator thickness decision variable is based on the available thickness types from the Shanghai Metals Market (SMM) (SMM, 2022), including three uncoated and three coated separator thicknesses. The uncoated separators have

a thickness of 5, 7 and 9 µm. The coated separators have an additional 2 µm layer for the 5 and 7 µm separator, and 3 µm for the 9 µm separator.

Table S5: Prices of different separator types with varying thickness. Price for December 2021 as reported by the Shanghai Metals Markets (SMM, 2022)

	**5 µm**	**7 µm**	**9 µm**	**5+2 µm**	**7+2 µm**	**9+3 µm**
**USD/m^2**	0.45	0.31	0.21	0.53	0.37	0.33

1.2.7 Strategy 7: Cell form factor

Increasing the size of LIB cells can impact the cost and performance of packs and is a strategy increasingly adopted by car and battery producers. Tesla, for example, moved from a 18650 cell (18 mm diameter, 65 mm long) to a 21700 cell (21 mm diameter, 70 mm long) (Li *et al.*, 2022) and recently announced the adoption of a 4680 cell (46 mm diameter, 80 mm long) (Lambert, 2021). Another example is the recently announced blade battery by car producer BYD, resembling 96 cm long prismatic cells that crosscut the entire length of the pack, eliminating the need for module housing and reducing packaging material significantly (Li *et al.*, 2022).

Although pack-level energy density does not improve significantly by increasing the cell size, the reduction in cell housing requirement and production improvements (fewer jelly roll insertions/closings/tab welding for prismatic and cylindrical cells) reduces cost of cells (Quinn *et al.*, 2018). Due to the underlying product design model used as described below (Section 2), only the cell thickness can be changed, while cell length and width are determined by the module and pack configurations. The design variable included for this strategy is therefore an increase in cell thickness. Two design parameters are included: a 25 mm thick cell representing a default prismatic cell based on Epp *et al.* (2022) and a 50 mm thick cell representing the larger cell.

1.2.8 Overview of the technology system map

The technology system map with all included LIB design choices based on the discussion above is illustrated in Figure S4. The coding technology system map coding system as proposed by Stefanova *et al.* (2014) is adopted to represent the three different relations between technology-sub system. These include: 1) is-a, representing a technology system of a generic type (e.g. different cathode active material types); 2) part-of, relations between technology systems decomposing the technology system into different sub-systems (e.g. cathode active material and battery cell); and 3) mutual exclusion, referring to the fact that two technological options cannot be implemented together (e.g. the choice of a solid state battery excludes several conventional LIB production processes).

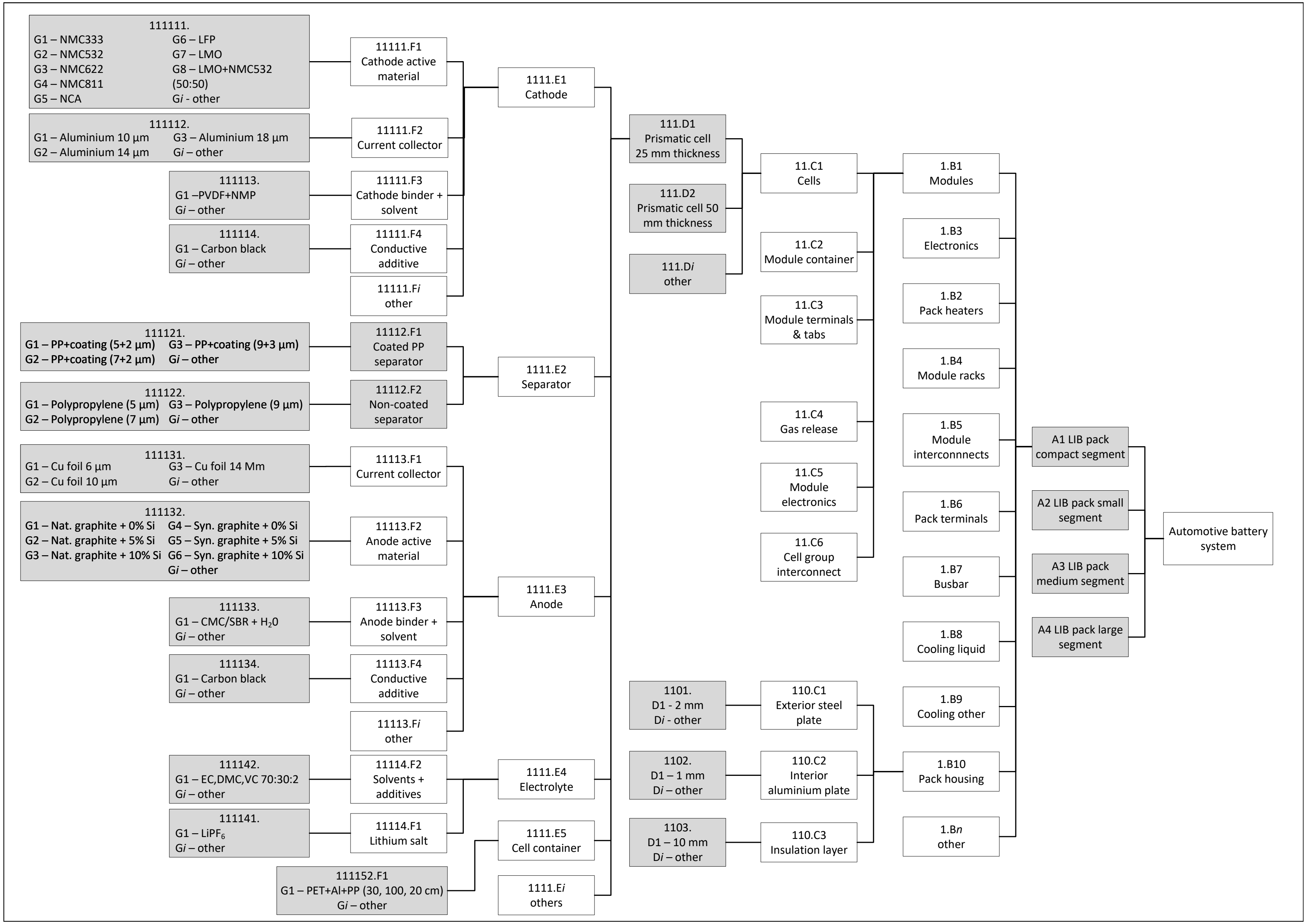

Figure S4: Technology system map of the lithium-ion battery design case study. The coding convention is based on Stefanova *et al.* (2014) to represent the relation between technology sub systems. Letters refer to the part-of relation, numbers to the is-a relation and the grey boxes to the mutual exclusion relation.

1.3 Context description

The main function of the context description is to limit the size of the technology map (i.e. only include the technologies relevant for the context) and link the goal and scope definition to the modelling phase. We propose to link the goal and scope definition to the modelling phase by identifying the relevant sustainability objectives in line with the macro-goal. Based on this, empirical models needed to calculate these indicators can be determined. For example, environmental life cycle emissions can be calculated with life cycle assessment models while material criticality can be calculated with substance flow analysis or criticality assessment models. This deviates from the approach by Stefanova *et al.* (2014) who suggest to identify several mechanisms (causal relations that connect two activities, e.g. environmental mechanisms as defined in ISO (2020)). However, most mechanisms remain vaguely described and structured approaches to identify these currently do not exist.

To illustrate the model, the perspective of a fictive European EV LIB producer is taken. The producer seeks to identify the optimal battery design strategies that can be implemented within a short timeframe (0 to 5 years). A cradle-to-gate system boundary is adopted with a temporal scope of one year. The use and recycling phase are therefore excluded as well as novel battery technologies still in the early development stage. The LIB factory location is based on the average values of the seven largest potential LIB-producing countries, including Germany, Sweden, Norway, UK, Poland, Hungary and France (CIC energiGUNE, 2021) and has an annual production volume of 500,000 packs (fully utilized).

In line with the macro-goal four indicators are considered: 1) battery cost; 2) carbon footprint; 3) material criticality; and 4) battery performance. The battery cost refers to the total cost of a battery pack to the consumer (EV producers) and is calculated using the life cycle cost (LCC) model formulation. The carbon footprint refers to the sum of the GHG emissions in the cradle-to-gate battery product system and is calculated with life cycle assessment (LCA). The material criticality indicator includes the following elements: Li, Co, Ni, Al, Cu, P, Fe, Si and C (natural graphite) and is calculated based on the factory substance flows using substance flow analysis. The technical performance indicator is based on the pack-level gravimetric energy density, one of the key performance indicators used in the European Strategic Research Agenda for Batteries (EC, 2020*a*), and is calculated based on an existing battery performance model.

2. Supporting information battery and vehicle design model

2.1 Battery and vehicle model

The most recent version of the BatPaC battery design model (version 5), developed by the Argonne National Laboratory (Knehr *et al.*, 2022), is used as the underlying product design model to obtain the bill of materials and performance of many battery designs. BatPaC is a bottom-up comprehensive battery design and cost model that is widely used in the research community to model battery costs (Duffner *et al.*, 2020*b*), and as input to LCA models[1] (Peters *et al.*, 2017). Based on one energy demand parameter (pack level energy (kWh) or capacity (Ah)), and several user-defined design parameters, the battery system is solved iteratively by varying the cell capacity and electrode thickness to match the desired battery energy. Based on the battery system solution, the bill of materials (BOM) and battery performance can be obtained.

A Python script was developed to automate the data extraction from Batpac, allowing for the fast calculation of multiple battery designs. As illustrated in Figure S1, user-defined parameters are sent to the BatPaC model in Excel, which iteratively solves the model, and the bill of materials ("3_MC_materials_pack_design") and battery pack performance parameters ("3_PAR_battery_parameters") are returned. A wide range of parameters on the cell, module, pack and vehicle level can be adjusted. An example notebook how to use the Python script can be found in the Github repository.

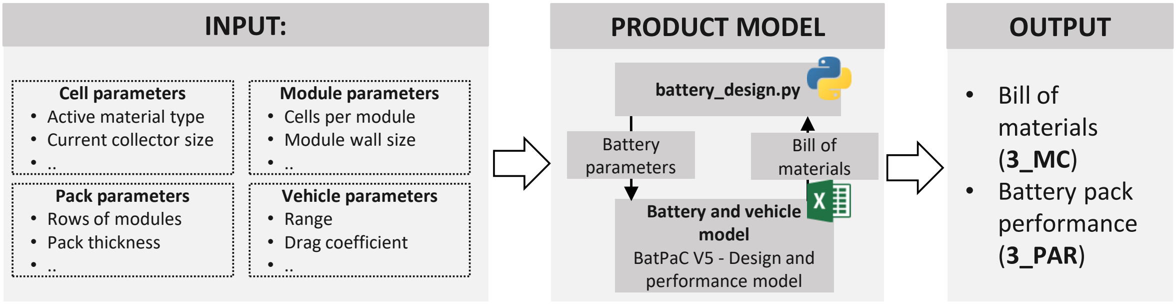

Figure S1: Workflow of the battery and vehicle design model.

Four changes to the BatPaC model were required to include several design parameters as defined in the technology map, but not present in the current model. These include

1. higher silicon percentage in the anode (10%)
2. change of default anode binder material
3. inclusion of coated separator
4. inclusion of a vehicle model to accommodate different vehicle segments

Following is a brief discussion of each of these.

[1]The Argonne LCA GREET model, for instance, uses BatPaC as input to calculate the inventories of battery systems (Dai *et al.*, 2018*a*; Winjobi *et al.*, 2020).

2.1.1 Silicon content in anode

In the most recent BatPaC version (Version 5), a 5 wt% Si content can be added to the anode active material, changing the anode active material capacity (mAh g^{-1}) accordingly. To allow for a larger amount of Si (up to 10 wt% as highlighted in the technology map), the practical anode active material capacity (cap_{neg_am}) and material density, g cm^3 (ρ_{neg_am}) parameters in BatPaC are adjusted based on Equation 2.1:

$$i_{neg\_am} = i_{gr} \times (1 - wt\_pct_{Si}) + i_{Si} \times wt\_pct_{Si} \tag{2.1}$$

where i refers to the material capacity, (cap) and density, (ρ) of the graphite (gr) and silicon (Si). The default BatPaC values of 360 and 2000 mAh g^{-1} were used for graphite and silicon capacity respectively and 2.24 g cm^3 for graphite density.[2] The density of Si, not included in the default calculation of BatPac, is 2.13 g cm^3 based on Greenwood *et al.* (2021). The wt_pct_{Si} in Equation 2.1 refers to the weight percentage of Si and is constrained to a value ranging between 0 and 10 wt%.

2.1.2 CMC binder

The value for the binder density in BatPaC is based on the density of polyvinylidene fluoride (PVDF). In this case study, however, a mixture of carboxymethyl cellulose (CMC) and styrene-Butadiene Rubber (SBR) is used for the anode binder as commonly found in current EVs (Kwade *et al.*, 2018; Li *et al.*, 2022). To include the density of the CMC-SBR anode, the BatPaC default value is updated accordingly. A default mixture of CMC-SBR is assumed to be 60:40 and has a final density of 1.336 g/cm^3 (Crenna *et al.*, 2021).[3] The ratio of CMC-SBR, however, can be changed within the model with parameter `perc_cmc_anode_binder`.

2.1.3 Coated and non-coated separator

The weight of the separator (wt_{sep}) is calculated as the sum of the required separator area (A_{sep}), the density (ρ_{sep}) and the thickness (th_{sep}).

$$wt_{sep} = A_{sep} \times \rho_{sep} \times th_{sep} \tag{2.2}$$

The separator area is calculated in BatPaC based on the length (L) and width (W) of the cathode, the separator overhang (OH) and the bicell layers. The length and width and amount of bicell layers of the cathode are based on BatPaC values. The separator overhang are changeable parameters (`sep_overhang_width` and `sep_overhang_length`), whereby the default BatPaC values of 2mm for the width and 4mm for length of the cathode are used if left unchanged.

$$A_{sep} = (L_{cath} + OH_{l,sep}) \times (W_{cath} + OH_{w,sep}) \times bicell\_layers \tag{2.3}$$

To account for the possibility of a coated separator, the default value for ρ_{sep} in BatPaC was

[2]For NMC811, the default values of 345 mAh g^{-1} and 2.2 g cm^3 for graphite were used.
[3]Based on a density of 1.59 g/cm^3 and 0.94 (g/cm^3) for CMC and SBR, respectively.

updated based on Equation 2.4.

$$\rho_{sep} = \frac{th_{sep\_film} \times \rho_{sep\_film} + th_{sep\_coating} \times \rho_{sep\_coating}}{th_{sep\_film}} \times V_{sep} \tag{2.4}$$

Thereby V_{sep} is the void fraction of porosity of the separator. The value for V_{sep} is based on the standard BatPaC chemistries, which is 50%. For the ρ_{sep_film}, the BatPaC default value (0.9 g cm^3) is used, representing the ρ of PP. The density of the silica-based coating layer is 1.996 g cm^3, assuming the coating consists of PVDF (26 wt.%), hexafluorpropylene (4 wt.%), dibutyl phthalate (40 wt.%) and silica (30 wt.%), as reported by Notter *et al.* (2010) and used in the ecoinvent 3.7.1 database.

2.1.4 Vehicle model

Vehicle characteristics such as size and energy consumption influence the battery energy requirement and the corresponding battery design. To account for these aspects, a vehicle model was added to BatPaC to calculate the energy storage requirement based on specific vehicle design parameters. The calculation is based on the model developed by Deng *et al.* (2017) and the underlying physics based model to estimate the energy-vehicle mass relation by Kim & Wallington (2016). As the total vehicle mass is dependent on the battery mass, Deng *et al.* (2017) established a linkage between the vehicle model with BatPaC. As illustrated in Figure S2, the vehicle and battery model interact by iteratively calculating the battery capacity and corresponding energy requirement for the vehicle. The following section is a description of the model and the parameters used.

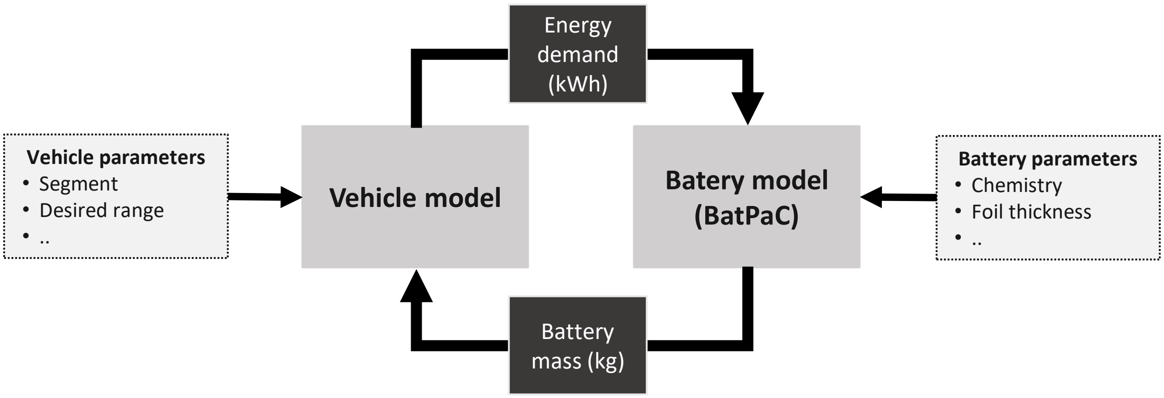

Figure S2: Vehicle model and BatPaC interaction. This interaction is based on the developed model by Deng *et al.* (2017).

The first step is to calculate the initial battery mass (wt^0_{batt}) of a pre-determined nominal battery capacity ($E^0_{nominal}$):

$$wt^0_{batt} = \frac{E^0_{nominal}}{\rho_{batt}} \tag{2.5}$$

where ρ_{batt} is the gravimetric density (Wh kg^{-1}) of the specific battery design, calculated with BatPaC. Based on the initial battery mass, the total vehicle mass (wt_{veh}) can be calculated with

Equation 2.6

$$wt_{veh} = wt_{batt} + wt_{glider} + wt_{motor} + wt_{transmission} \tag{2.6}$$

where the glider weight (wt_{glider}) is based on the mass compounding coefficient of 0.57 kg/wt_{veh} and the weight of the transmission ($wt_{transmission}$) and motor (wt_{motor}) are calculated by multiplying the mass-power coefficients of 0.585 kg kW^{-1} for the transmission and 0.9 kg kW^{-1} for the motor with the power of the battery ($P_{battery}$) (Deng *et al.*, 2017). The $P_{battery}$ was calculated from reported motor power (P_{motor}) of specific vehicles similar to Safoutin *et al.* (2018) and thereby accounting for a motor power loss, η_{motor}, of 8% (Deng *et al.*, 2017) and an EOL power fade, EOL_{power}, of 20% (Safoutin *et al.*, 2018):

$$P_{battery} = P_{motor} \times \eta_{motor} \times EOL_{power} \tag{2.7}$$

Following the mass of the vehicle, the desired mass-induced fuel consumption, here referred to as traction energy ($E_{traction}$), can be calculated with the physics-based model by Kim & Wallington (2016). Equation 2.8 calculates the $E_{traction}$ (kWh) needed for a specific driving cycle (i), where i includes the US Environmental Protection Agency's (EPA) Urban Dynamometer Driving Schedule (UDDS) and the Highway Fuel Economy Test (HWFET):

$$E_{traction,i} = \int (Av_i + Bv_i^2 + Cv_i^3 + a_i \times v_i \times (1 - \theta \times \mu \times \eta_{charging}) \times wt_{veh})dt \tag{2.8}$$

v_i and a_i are the driving speed (m/s) and acceleration (m/s^2) respectively for each driving cycle i. The values for these are obtained from Kim & Wallington (2016) and Deng *et al.* (2017). A, B and C are the rolling (N), rotating (N/(m s)) and aerodynamic resistance (N/(m/s)^2) coefficient respectively. These coefficients, also referred to as the target coefficients, are used by the EPA to calibrate the resistance of the dynamometer rollers' for fuel economy testing and are publicly available for all registered vehicles in the US (EPA, 2021*a*).

The last terms in Equation 2.8 were used to calculate the kinetic energy captured via the regenerative braking system. This was obtained by reducing the mass of the vehicle by the sum of the braking to kinetic energy ratio (θ), the regenerative braking efficiency (μ) and the charging efficiency ($\eta_{charging}$). The average values for μ (5%) and θ (0.74 UDDS and 0.41 HWFET) as recommend by Kim & Wallington (2016) were used as default parameters. The charger efficiency, ($\eta_{charging}$) is set to 90% for all battery designs as commonly applied in LIB LCA studies (Peters *et al.*, 2017).

Based on the required traction energy requirement, the total nominal battery energy ($E_{nominal}^{required}$) was calculated with Equation 2.9 to 2.11 based on Deng *et al.* (2017):

$$E_{discharged} = \sum_i \frac{a_i \times D}{\int v_i^{dt}} \times \frac{E_{traction,i}}{\eta_{motor} \times \eta_{transmission}} + D \times P_{aux} \times (a\frac{\int dt_i}{\int v_i}) \tag{2.9}$$

$$E_{stored} = \frac{E_{discharged}}{\eta_{discharging}} \tag{2.10}$$

$$E_{nominal}^{required} = \frac{E_{stored}}{UR} \tag{2.11}$$

Equation 2.9, calculates the total discharged energy for a required range (D) as the sum of the energy demand from auxiliary devices (heating, cooling, etc.), P_{aux}, and the traction energy demand ($E_{traction}$) for both the urban and highway driving cycle (i) as calculated in Equation 2.8. The total nominal energy of the battery is calculated with the total stored energy and the available energy (UR). An overview of all non vehicle specific parameter is presented in Table S1.

Furthermore, the traction energy demand ($E_{traction}$) in Equation 2.9, is adjusted to account for the efficiency of the motor (η_{motor}, 0.89) and transmission ($\eta_{transmission}$, 0.93) (Deng *et al.*, 2017). a in Equation 2.9 refers to the share of city or highway driving for distance D. The standard EPA fuel economy testing ratio of 55% city and 45% highway driving were used. A discharge efficiency ($\eta_{discharging}$) is 90% based on the charging efficiency.

Table S1: Non vehicle specific parameters used for the vehicle model. Acronyms: Urban Dynamometer Driving Schedule (UDDS); Highway Fuel Economy Test Cycle (HWFET)

Parameter	Notation	Value	Unit	Source
Motor efficiency	η_{motor}	92	%	[c]
Transmission efficiency	$\eta_{transmission}$	93	%	[c]
Charging efficiency	η_{charge}	90	%	[d]
Discharging efficiency	$\eta_{discharged}$	90	%	[d]
Highway/urban ratio	a	%		
Transmission mass-power	$wt_{transmission}^{coef}$	0.585	kg/kW	
Motor mass-power	wt_{motor}^{coef}	0.9	kg/kW	
Glider compounding mass	wt_{glider}^{coef}	0.57	kg/kg	
End-of-life power fade	-	20	%	[e]
Share of UDDS driving	a	55	%	[c]
Share of HWFET driving	b	45	%	[c]
Braking to kinetic energy ratio	θ	0.74[a] 0.41[b]	ratio	[f]
Regenerative breaking efficiency	μ	5	%	[c]
-	$\int avdt$	2,098[a] 1,165[b]	m^2/s^2	[f]
-	$\int vdt$	11,990[a] 16,507[b]	m	[f]
-	$\int v^3dt$	2,630,301[a] 8,540,124[b]	m^3/s^{-2}	[f]
-	$\int v^3dt$	164,025[a] 371,739[b]	m^2/s	[f]
-	$\int dt$	1 369[a] 765[b]	m^2/s	[f]

[a] For the UDDS
[b] For the HWFET
[c] Deng *et al.* (2017)
[d] Peters *et al.* (2017)
[e] Safoutin *et al.* (2018)
[f] Kim & Wallington (2016)

2.2 Case study parameters

Following is a brief overview of the default battery and vehicle design parameter inputs used for the case study.

2.2.1 Default battery model inputs

For all battery design options, several key battery design parameters were kept the same (Table S2). BatPaC allows for three different electric drive train systems (HEV, PHEV and fully EV) but only the EV type is used. Fast charge is included for all battery designs and a charging time of 60 min (15-95% state of charge (SOC)) is assumed based on the default values in BatPaC. The maximum positive electrode thickness was constrained to 74 µm based on Ahmed *et al.* (2021). The usable battery energy (UR in the vehicle model) was set to 94%

for all designs, based on the technical specifications of the Volkswagen ID.4 (Volkswagen, 2021). Finally, the BatPaC default ratio for cathode and anode active material, carbon and binder were left unchanged.

Table S2: Default battery design parameters left unchanged for each battery design in each model run.

**Parameter**	**Value**	**Unit**
Vehicle type	EV	-
Calculate fast charge	Yes	-
Recharge time (15-95% SOC)	60	minutes
Maximum charge power	150	kW
Maximum positive electrode thickness	74	µm
Available battery energy	94	%
Cathode ratio - active material:carbon:binder	96:2:2	-
Anode ratio - active material:carbon:binder	98:0:2	-

2.2.2 Default vehicle model inputs

The default input parameters for the vehicle model are based on the four vehicle segments included in the case study (mini, small, medium and large). The default input parameters include the rolling, rotation and aerodynamic resistance coefficients, the required driving distance, auxiliary power requirement and motor power requirement (Table S3).

Table S3: Segment specific parameters for the vehicle model.

**Parameter**	**Notation**	**Unit**	**Segment**[a]			
			Micro	Small	Medium	Large
Rolling resistance[d]	A	N	101.86	110.59	136	209.4
Rotation resistance[d]	B	$N/(m/s)$	3.42	-1.99	3.73	2.59
Aerodynamic resistance[d]	C	$N/(m/s)^2$	0.37	0.46	0.41	0.47
Distance[b]	D	km	200	322	411	460
Auxiliary power[c]	P_{aux}	W	475	575	715	775
Motor power[d]	P_{motor}	kW	55	150	150	209

[a] The A, B and C coefficients are based on the following vehicle models: Smart Fortwo Electric Drive 2019 model (micro); Hyundai Kona Electric 2019 model (small); Volkswagen ID. 4 82 kWh (medium); Ford Mustang Mach-e (large).
[b] Based on the average WLTP range for all vehicles of each segment as stated on EV-Database.org.
[c] Values are based on the base case of Cox *et al.* (2020).
[d] Values are based on vehicle model specific testing reports from the EPA obtained through EPA (2021*b*).

To obtain vehicle specific parameters for each segment, we used the segment classification by the EV-Database (2021) for all available EV models. The rolling, rotation and aerodynamic resistance coefficients and motor power were obtained from the EPA fuel economy database and corresponding testing reports (EPA, 2021*a*,*b*). However, EPA fuel economy reports are only available for a few EV models as reported on ev-database.org (Figure S3). We therefore

used four representative vehicle models for each segment with available EPA data: Smart Fortwo Electric Drive 2019 model (micro segment); Hyundai Kona Electric 2019 model (small segment); Volkswagen ID. 4 82 kWh (medium segment); Ford Mustang Mach-e (large segment). To include the differences in P_{aux} for different vehicle sizes (e.g. heating demand is higher for larger vehicles), the base case power demand for mini, small, medium and large vehicles as reported by Cox *et al.* (2020) were used. This includes a power demand for base electrical equipment (75W) and a segment-specific heat and cooling demand.

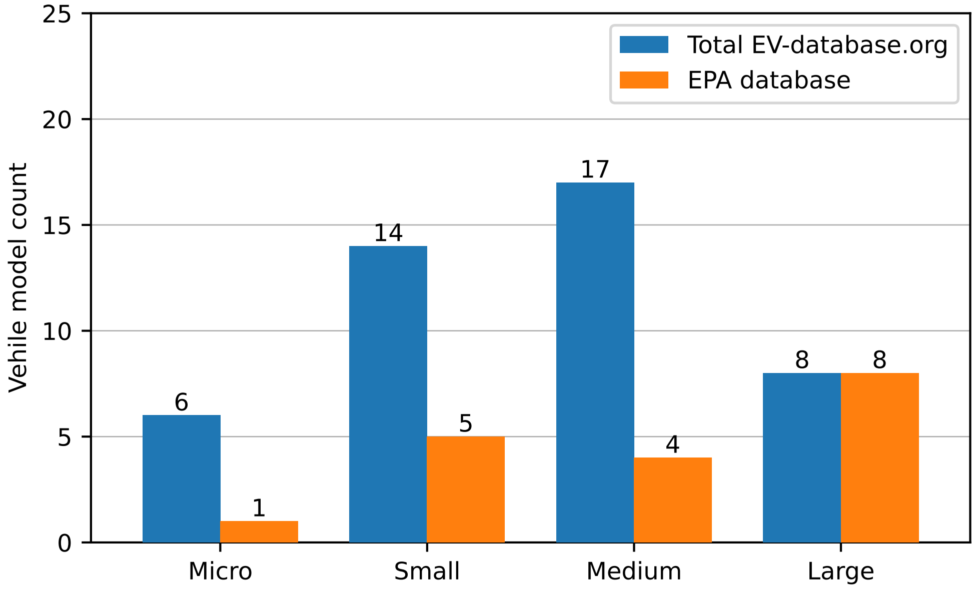

Figure S3: All electric vehicle models by segment reported on EV-Database (2021) and their availability in the EPA fuel economy testing database (EPA, 2021*a*). Vehicle model types are aggregated as one type (e.g. Kia Soul 39 kWh and 64 kWh version as Kia Soul).

The required distance for each vehicle segment is based on the average range of all currently available BEV models for each segment, as reported on the EV-Database (2021). The Worldwide harmonized Light vehicles Test Procedure (WLTP) values for each available BEV model for all segments were used. This resulted in a desired range of 200, 322, 411 and 460 km for micro, small, medium and large vehicles respectively (Figure S4).

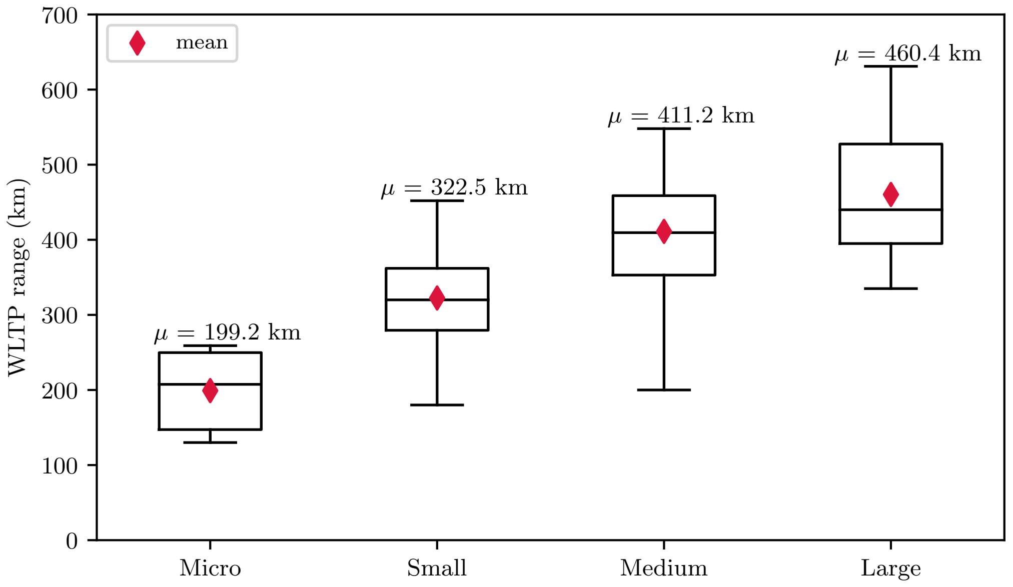

Figure S4: Electric vehicle range based on the Worldwide Harmonised Light Vehicle Test Procedure (WLTP) for all vehicle models and types as reported on the EV-Database (2021).

2.2.3 Default battery geometry configurations

To make sure the designed pack in BatPaC fits the vehicle size, battery pack geometry parameters were defined for all vehicle types. In BatPaC, the battery length, width and height cannot be directly changed, but instead are determined by four main parameters on the cell, module and pack level, see Figure S5. These include the total modules per row, the rows of modules, total cells per module, and the thickness of the cell.

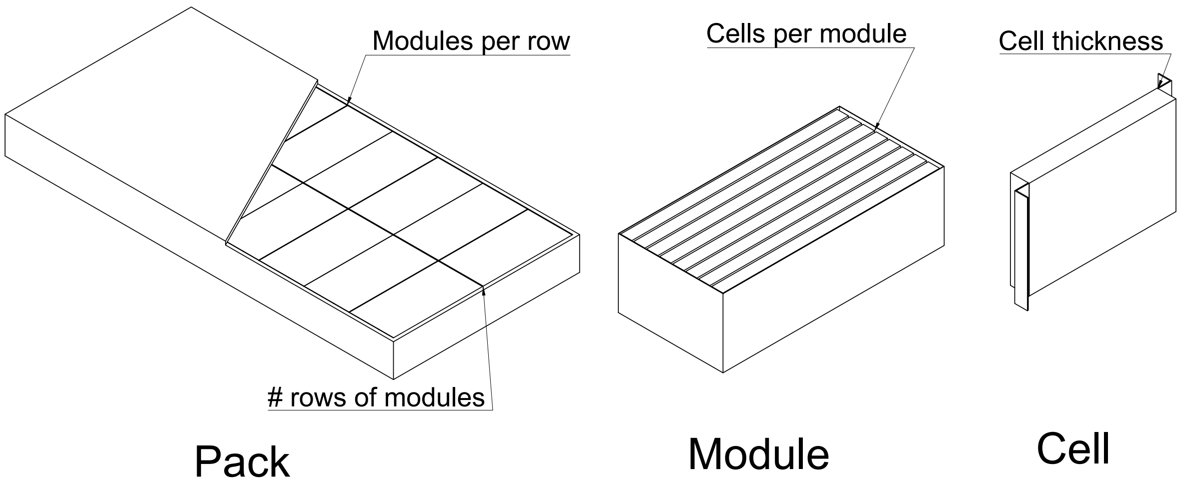

Figure S5: BatPaC pack, module and cell format parameters.

For the battery height, a standard value of 160 mm was used based on the reviewed vehicle models by Sankaran & Venkatesan (2021). The width of the battery pack for all segments is fixed while the length changes based on the desired battery capacity. The width for all segment was fixed to 1,100 mm. This is within the total vehicle width for all four representative

vehicle models (see Table S4) while accommodating an additional crash intrusion space of 300 mm per side measured from the outer vehicle as commonly found in EV models (Sankaran & Venkatesan, 2021). The width of the battery pack can be fixed in BatPaC with the cell thickness, cells per module and total rows of modules parameters. The configurations are thereby based on optimised pack configuration by Epp *et al.* (2022), which includes a 25 mm thick prismatic cell, 20 cells per module and 2 modules per row. For packs with a 50 mm thick cell, the total cells per module is 10.

As a battery is packed within the front and rear axles of the vehicle, the total battery length can be determined by the vehicle wheelbase and the battery to wheelbase ratio (BL/WL) (Sankaran & Venkatesan, 2021). The maximum pack length for each vehicle segment is therefore based on the wheelbase of the four representative vehicles and a BL/WL of 0.92, the largest BL/WL found in different EV models as reported by Sankaran & Venkatesan (2021). The total length of the battery pack was roughly estimated by changing the total rows of module parameter in BatPaC for a base battery with a NMC333 chemistry for each segment to find the best fit. The total modules per segment are 3, 4, 5 and 6 for the compact, small, medium and large based on the length and height constraints (Figure S6) and a relatively equal cell capacity (Figure S7). To regulate the pack voltage, two cells are connected in parallel for packs with a 25 mm thick cell while all cells are connected in series for packs with a 50 mm thick cell (Figure S8). A complete overview of all battery configurations can be found in Table S4.

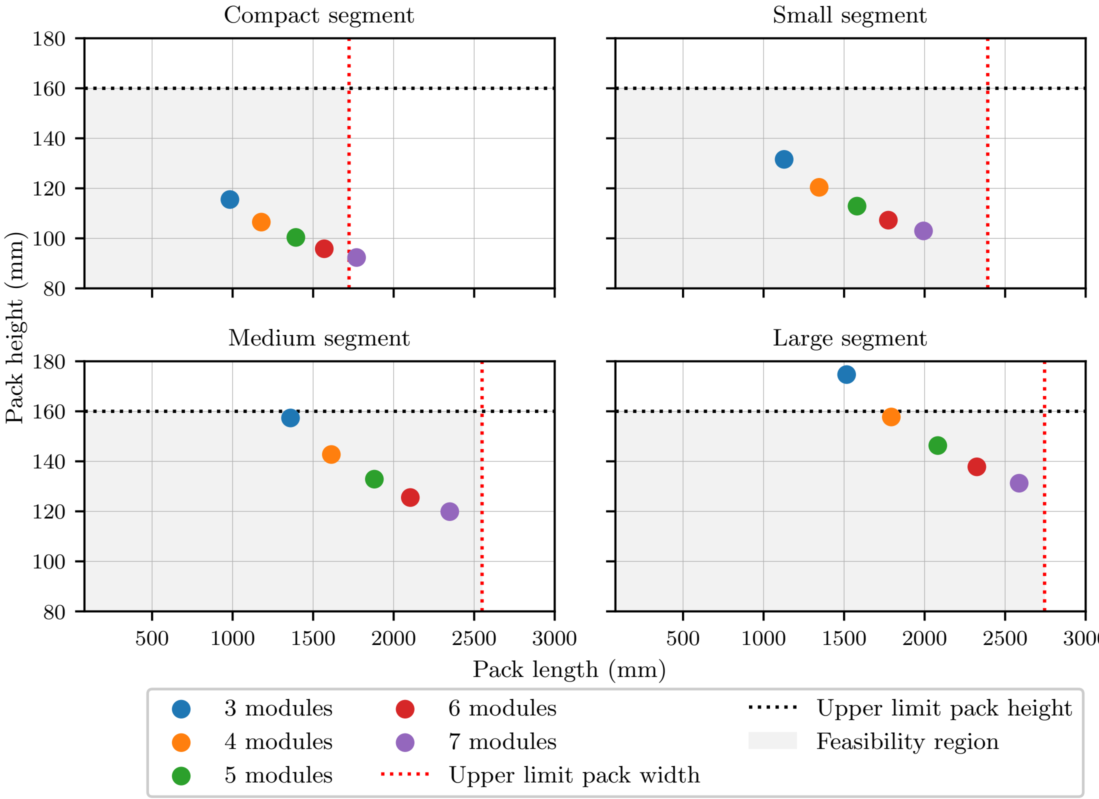

Figure S6: Battery height and length results by changing the rows of modoules for each vehicle segment for a base case battery (NMC333).

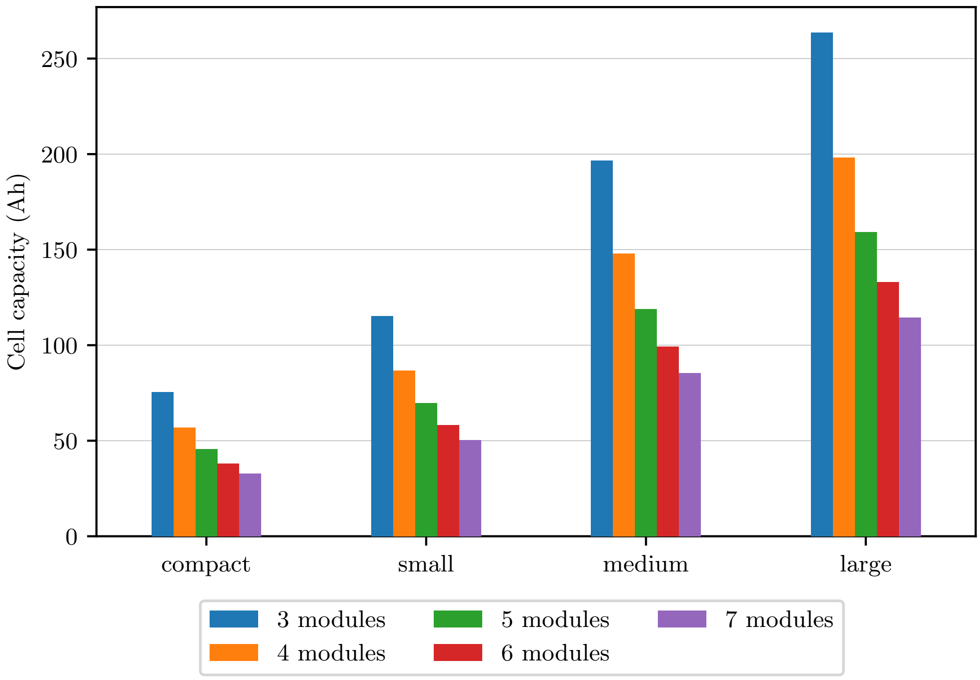

Figure S7: Cell capacity results by changing the rows of modules for each vehicle segment for a base case battery (NMC333).

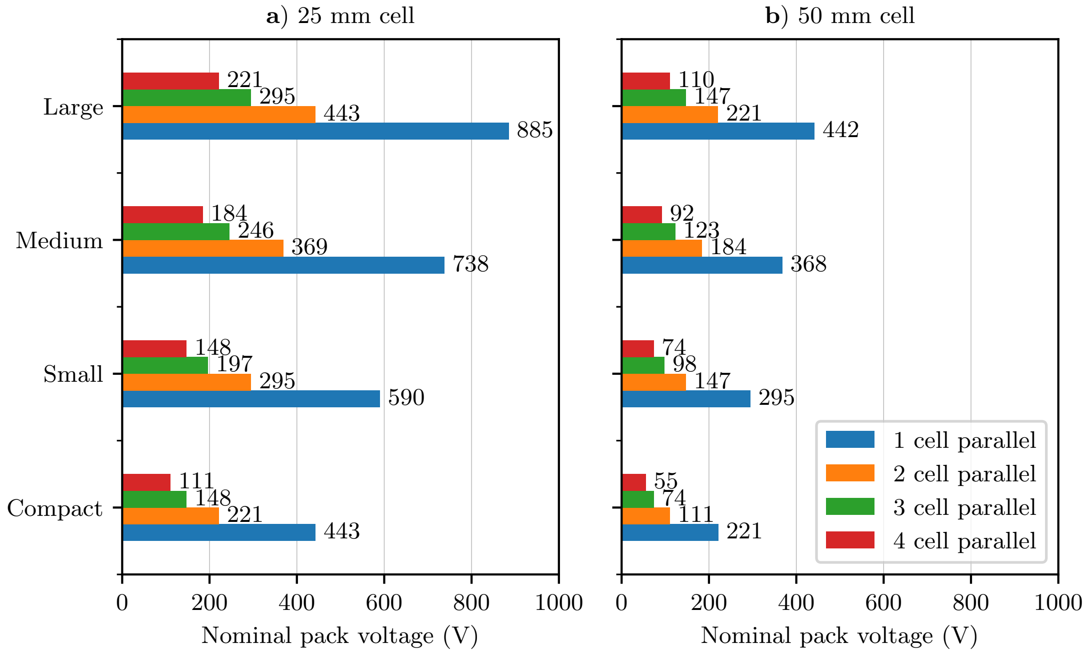

Figure S8: Pack voltage results by changing the cells in parallel for each vehicle segment for a base case battery (NMC333).

Table S4: Default battery configurations for each vehicle segment. The wheelbase are based on the four representative vehicles, including: Smart Fortwo Electric Drive 2019 model (micro segment); Hyundai Kona Electric 2019 model (small segment); Volkswagen ID. 4 82 kWh (medium segment); Ford Mustang Mach-e (large segment).

	**Micro**	**Small**	**Medium**	**Large**
Vehicle width (mm)	1663	1800	1.852	1.881
Vehicle wheelbase (mm)	1,873	2,600	2,771	2,984
Battery to wheelbase ratio	0.92	0.92	0.92	0.92
Max pack length (mm)	1,723	2,392	2,549	2,745
Max pack height (mm)	160	160	160	160
Pack width (mm)	1,100	1,100	1,100	1,100
Cells per module (25 mm cell)	20	20	20	20
Cells per module (50 mm cell)	10	10	10	10
Modules per row	2	2	2	2
Rows of modules	3	4	5	6
Total cells (25 mm cell)	120	160	200	240
Total cells (50 mm cell)	60	80	100	120
cells in parallel (25 mm cell)	2	2	2	2
cells in parallel (50 mm cell)	1	1	1	1
modules in parallel	1	1	1	1

3. Supporting information foreground system

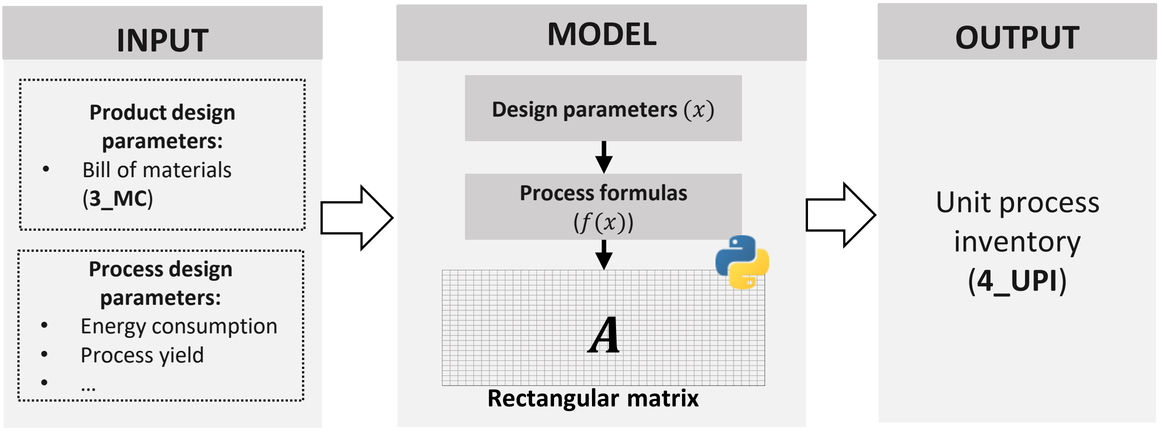

Figure S1: Workflow of the foreground material and energy flow model.

3.1 Energy consumption battery production

Following is a discussion on the energy consumption for battery production and the values used in this research.

3.1.1 Overview of literature values

Early LCA studies of battery production such as Ellingsen *et al.* (2014), Notter *et al.* (2010) or Dunn *et al.* (2015) were based on pilot-scale facilities or modelled estimates. More recently, several studies published data on energy consumption based on industry sources, see Table S1. However, large discrepancies in energy consumption can be observed between studies. Production capacity in general has a large impact on the energy consumption (Davidsson Kurland, 2019; Jinasena *et al.*, 2021; Dai *et al.*, 2019*a*), as can be observed in Table S1.

Table S1: Industry based reported energy consumption data, including heating, electricity and cooling for battery cell production steps. Unit is Wh per 1 Wh of cell energy. Data updated from Jinasena *et al.* (2021).

	**Yuan *et al.* (2017)**	**Kim *et al.* (2016)**	**Pettinger & Dong (2016)**	**Dai *et al.* (2017*b*)**	**Degen & Schütte (2022)**	**Chordia *et al.* (2021)**	**Sun *et al.* (2020)**
**Mixing**	0.88	-	2.64	-	0.13	-	1
**Coating**	51.2	-	15.42	15.6-19.7	11.02	-	-
**Calendering**	3.04	-	5.97	-	0.53	-	-
**Stacking**	6.16	-	5.97	-	0.41	-	-
**Final drying**	-	-	5.97	-	1.61	39.42[a]	12.7
**Filling**	4.72	-	1.53	-	1.59	2.76[b]	-
**Formation**	0.56	-	2.92	1.11	13.54	14.77	3
**Dry room**	31.2	-	-	17.5-26.9	10.68	-	11.7
**Pack assembly**	-	-	-	-	-	-	1
**Other**	8.48	-	5.56	-	1.98	34.81	-
**Total**	106	147[c]	46	34-47	41.49	92[c]	28
**Annual production capacity (GWh)**	0.02	0.06[b]	0.08	2	8	16	30

[a] Quantity refers to total cathode and anode production energy.

[b] Includes electrolyte mixing, feeding and cell assembly.

[b] Calculated based on capacity of 1 million cells per year. Pack is 23 kWh and includes 430 cells.

[c] Excluded energy consumption from cathode active material production.

[d] Includes module and battery assembly which only represent a small proportion of energy consumption (Sun *et al.*, 2020). Calculated based on the reported primary energy of 120 MJ kg−1 battery, battery weight and capacity of 303 kg and 24 kWh and a primary to electric energy conversion factor of 0.35 as used by the authors.

Studies that used giga-scale facilities energy consumption (Dai *et al.* (2017*a*); Degen & Schütte (2022); Chordia *et al.* (2021); Sun *et al.* (2020)) are all in a similar range. Sun *et al.* (2020) reports the lowest energy consumption levels (28 Wh^{-1} per Wh^{-1} of cell energy) for a 30 GWh operating plant in China. A relatively high-energy consumption, 92 Wh^{-1} per Wh^{-1} of cell energy, can be observed from the study by Chordia *et al.* (2021). The underlying data, obtained from the technical reports from a facility to be build in Sweden, is based on a simulation model. Further process optimisation of an actual operating plant is expected to improve energy efficiency (e.g. the authors cite the example of cell formation, which could be improved by up to 40%). Furthermore, the authors include several additional processes such as cell container manufacturing (2.76 Wh Wh^{-1}), waste water treatment (6.99 Wh Wh^{-1}) and facility operations

and utilities (27.15 Wh Wh^{-1}) that none of the other studies report.

3.1.2 Overview of energy consumption calculation by Degen & Schütte (2022)

Due to the detail of the process steps, calculation and availability of the data, the energy consumption calculation and data by Degen & Schütte (2022) is used. The authors base their data on the factory plans of a 7 GWh cylindrical manufacturing plant in German and calculate the energy consumption from machine specifications of different manufacturers, which include statements about energy consumption.[1] Degen & Schütte (2022) calculate the total energy input (E), for both electricity and gas (n) for each battery production process (bp) based on the process machine power (P_{bp}) and the annual machine hours ($prod\_time$) to produce the annual cell capacity ($prod\_volume_{yr}$):

$$E_{n,bp} = -\frac{P_{bp} \times prod\_time_{yr}}{prod\_volume_{yr}} \tag{3.1}$$

The annual production time used by the authors is 8,760 h yr^{-1}. The used production volume is 883.01 MWh yr^{-1}, equivalent to 1,75 million 21700 cylindric cells.

The hourly machine power ($P_{bp,n}$) for each production step is based on the nominal machine power ($P_{nom,bp}$), the total machines required (M_{bp}) for each process, the hourly producible cell capacity of each process at nominal power ($prod\_volume_{hr,bp}$)) and total cell output per hour ($prod\_volume_{hr}$)):

$$P_{bp,n} = \frac{P_{nom,bp} \times M_{bp}}{prod\_volume_{hr,bp} \times prod\_volume_{hr}} \tag{3.2}$$

Degen & Schütte (2022) obtained the nominal machine power for each process steps from three different machine manufacturers with slightly different reported power requirements. The authors use the average of this to calculate the total energy requirements (Table S3).

[1]The cell form format in the study by Degen & Schütte (2022) is cylindrical while the cell used in the product model and BatPaC is prismatic. However, as pointed out by the authors, the cell form factor has little influence on the energy consumption as the resulting changes occur during cell assembly, which has only a small impact on the overall energy consumption.

Table S3: The nominal machine power (kW) requirement for each battery production step in a low, medium and high scenario. Data obtained from Degen & Schütte (2022) and based on three manufacturers. The scenarios highlight the minimum, average or maximum value for each production process across the three manufacturers.

	Nominal machine power (kW)					
**Process**	***Electricity***			***Natural gas***		
	Low	*Average*	*High*	*Low*	*Average*	*High*
Mixing	15	19	30	0	0	0
Coating and drying	60	74	95	750	823	875
Calendaring	55	60	70	0	0	0
Slitting	40	45	50	0	0	0
Vacuum drying	5	7	10	200	210	230
Winding	15	24	30	0	0	0
Assembly	150	160	175	0	0	0
Washing	160	198	225	0	0	0
Formation	850	994	1100	20	25	30
Ageing	0	0	0	30	39	55
Testing	100	100	100	0	0	0
Material handling	180	170	250	0	0	0
Drying rooms	175	150	164	872	912	950

3.1.3 Application to this research

The above calculations and data were used to estimate the battery energy consumption for each production process in this research. The final energy consumption by Degen & Schütte (2022) however is based on the process energy consumption per kWh of cell. A multiplication of this by the capacity of cells with different characteristics (i.e. lower or higher capacity) would result in accurate results. Instead, cell weight is used here to calculate the energy consumption per process. To convert the energy consumption from Wh/Wh to Wh/kg cell, we use the gravimetric energy density of the cells. The density of the cell used by Degen & Schütte (2022) is estimated to be around 124 Wh/kg based on a nominal energy of 8.38 Wh and assuming a cell weight of 67 gr (reported weight ranges from 63 to 70 gr (Quinn *et al.*, 2018; Lain *et al.*, 2019)). This is comparatively low density can be explained by the fact that the cells are power optimised Degen & Krätzig (2021).

The resulting energy consumption used in this study are provided in Table S4. As illustrated in the table, all energy consumption units are based on cell mass, except for the formation step and the module and pack assembly. During the formation step, the cell is charged and discharged (see also Section 3.2.9), and the cell capacity a better estimate to calculate the energy consumption. The module and pack assembly consumption are not included in the data by Degen & Schütte (2022) but instead taken from Sun *et al.* (2020). This data is converted from kWh/kWh to Wh/kg of battery pack based on the reported pack density of 0.115 kWh kg^{-1}

in the study of Degen & Schütte (2022). Furthermore, although scrap rates are not disclosed by Degen & Schütte (2022), it is assumed that this data includes a 5% cell scrap rate during cell testing (Duffner *et al.*, 2021; Nelson *et al.*, 2019). The total energy consumption is adjusted accordingly. For the case study, the average energy consumption scenario is used.

Table S4: Electricity and gas consumption for each cell production process based on Degen & Schütte (2022) and module and pack assembly based on Sun *et al.* (2020). Original data units converted from kWh to kg based on a cell specific energy of 0.124 kWh kg^{-1} and 0.115 kWh kg^{-1} for module and pack assembly. It is assumed that the data by Degen & Schütte (2022) includes a 5% cell scrap rate after the testing stage. The presented data excludes this scrap rate from the mixing to testing phase.

**Process**	**Unit**	**Electricity**			**Natural gas**		
		Low	*Avg.*	*High*	*Low*	*Avg.*	*High*
Mixing	kWh kg^{-1} cell	0.012	0.015	0.024	-	-	-
Coat & dry	kWh kg^{-1} cell	0.086	0.106	0.137	1.079	1.184	1.259
Calendaring	kWh kg^{-1} cell	0.062	0.068	0.079	-	-	-
Slitting	kWh kg^{-1} cell	0.018	0.020	0.023	-	-	-
Vacuum drying	kWh kg^{-1} cell	0.005	0.007	0.010	0.193	0.202	0.222
Winding	kWh kg^{-1} cell	0.017	0.028	0.035	-	-	-
Cell assembly	kWh kg^{-1} cell	0.175	0.186	0.204	-	-	-
Washing	kWh kg^{-1} cell	0.186	0.230	0.262	-	-	-
Formation	kWh kWh^{-1} cell	8.011	9.425	10.367	0.188	0.236	0.283
Ageing	kWh kg^{-1} cell	-	-	-	0.035	0.045	0.064
Testing	kWh kg^{-1} cell	0.117	0.117	0.117	-	-	-
Building support systems	kWh kg^{-1} cell	0.221	0.209	0.307	-	-	-
Drying rooms	kWh kg^{-1} cell	0.215	0.184	0.201	1.070	1.119	1.165
Pack assembly	kWh kWh $^{-1}$ pack	0.115	0.115	0.115	-	-	-

3.2 Foreground inventories

The foreground inventories refer here to all material, waste and energy flows within the battery manufacturing system, see Figure S2 below. Following is a description of the inventory calculation of each process.

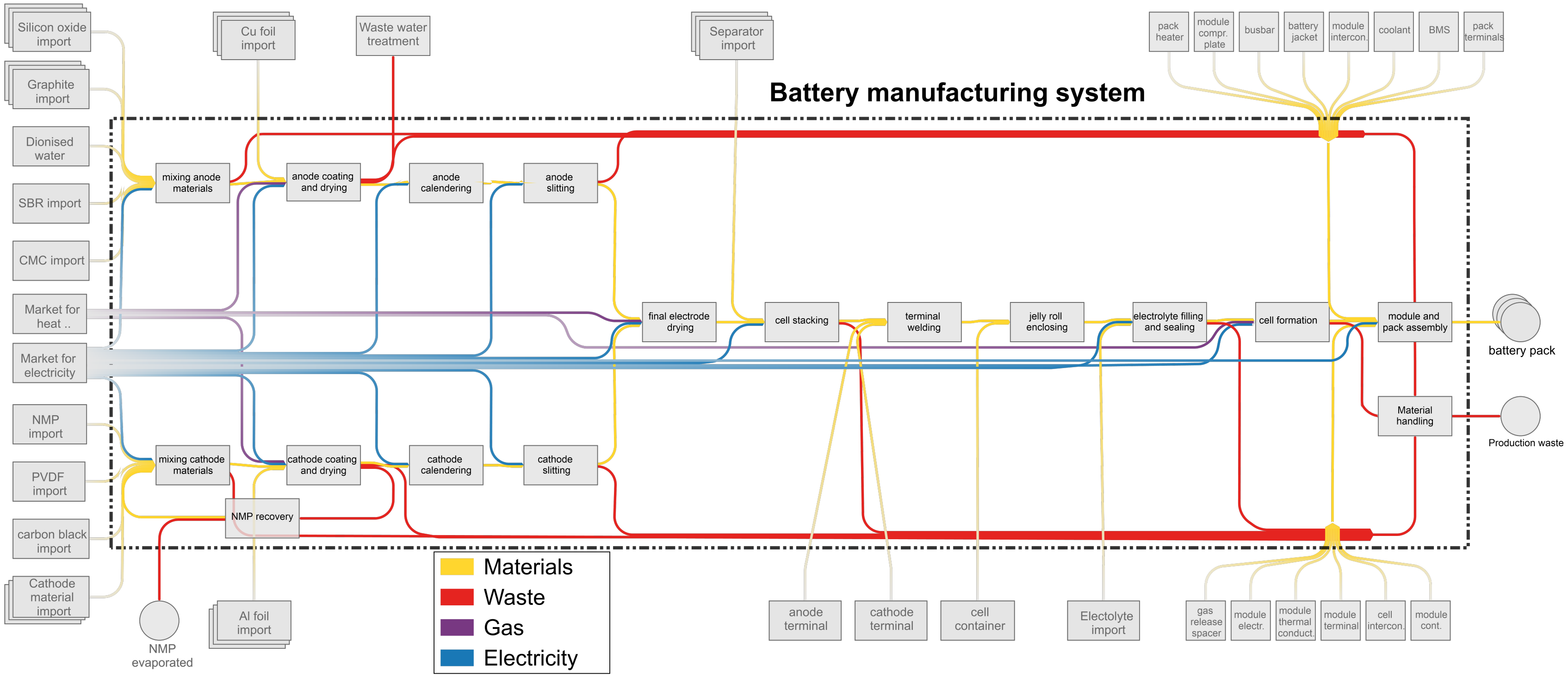

Figure S2: Material and energy process flow structure of a lithium-ion battery manufacturing plant (foreground system). Stacked processes refer to technology choices.

3.2.1 Inventory active material mixing

In the first production step, the anode and cathode active materials are mixed with the binder, a conductive agent (carbon black) and binder solvent to obtain a wet slurry. The mixture ratio are based on the default values for energy optimisation chemistry's in BatPaC Version 5. This include active material, binder and carbon black ratios of 96:2:2 and 98:0:2 for the cathode and anode respectively. The inventory for the active material mixing process is highlighted in table S11.

For the CMC-SBR anode binders, water is used as a solvent, while for the cathode slurry and PVDF binder, N-methyl-2-pyrrolidone (NMP) is used (Kwade *et al.*, 2018; Peters & Weil, 2018; Dai *et al.*, 2019*a*). The total binder solvent requirement is based on the binder:solvent ratio of 1:24 as used in BatPaC. The solvents are evaporated during the coating and drying process. It is assumed that all water is lost to the environment and most NMP is recovered and recycled (Nelson *et al.*, 2019; Bryntesen *et al.*, 2021). Only the evaporated NMP that needs to be replaced is accounted for, a similar assumption to Notter *et al.* (2010), Crenna *et al.* (2021) and the BatPaC cost model. An NMP recovery rate of 99.5% is used (Crenna *et al.*, 2021; Nelson *et al.*, 2019).

All material inputs are based on the material content of the specific battery design determined in the product model and the process yield parameters. The process yield parameters for materials m in the anode and cathode mixing process is based on the product of the electrode yield parameters ($py_{i,electrode}$) across all relevant processes (i) including: material mixing (py_{mix}), slurry coating (py_{coat}), electrode slitting (py_{slit}), cell stacking (py_{stack}) and cell formation and ageing (py_{age}) (see also table S5).

Table S5: Overview of the default process scrap rates used in the case study based on BatPaC (Knehr *et al.*, 2022)

Process	Material	Scrap rate
Mixing	Active material	1%
Coating and drying	Slurry	5%
Coating and drying	Foil	1%
Calendering and slitting	Slurry	1%
Calendering and slitting	Foil	8%
Stacking	Electrode	1%
Stacking	Separator	2%
Filling & sealing	Electrolyte	6%
Aging	Cell	5%

Energy consumption for the mixing step ($electricity_{mixing}$) consist of electricity required for the mixer. In the energy consumption data obtained from Degen & Schütte (2022), the

authors do not differentiate between cathode and anode mixing. However, different electricity consumption rate can be expected due to the material properties and mixing requirements. Here a 65:35 ratio of electricity consumption for the anode and cathode material mixing based on a model of the mixing time and material properties of anode and cathode powders (NMC333 and graphite) by Jinasena *et al.* (2021). The electricity consumption is adjusted to account for the cell yield rate. See Table S11 and **??** for an overview of the parameterised mixing process inventories.

Table S6: Process inventory for anode active material mixing for 1 battery pack

**Description**	**Quantity/function**	**Unit**	**Inventory**
**Input: materials**			
Active material	$anode\_am_m/\prod_i py_{i,electrode}$	kg	Section 5.1.1
Binder (CMC)	$anode\_binder_{cmc}/\prod_i py_{i,electrode}$	kg	Section 5.3.1
Binder (SBR)	$anode\_binder_{sbr}/\prod_i py_{i,electrode}$	kg	Section 5.3.1
Binder solvent	$24^{a} * \sum_{sbr,cmc} anode\_binder/\prod_i py_{i,electrode}$	m^3	Section 5.3.1
Carbon black	$anode\_cb/\prod_i py_{i,electrode}$	kg	Section 5.3.1
**Input: process**			
Electricity	$0.35 * wt\_cell * electricity_{mixing}/py_{cell}$	kWh	Market for electricity, medium voltage
**Output: reference product**			
Anode slurry	$\sum anode_{am,binder,cb,solvent} \prod_i py_{i,electrode}$	kg	
**Output: waste**			
Waste active material	$anode\_am_m * py_{mix}$	kg	
Waste binder (CMC)	$anode\_binder_cmc * py_{mix}$	kg	
Waste binder (SBR)	$anode\_binder_sbr * py_{mix}$	kg	
waste carbon black	$anode\_cb * py_{mix}$	kg	

[a] 1:24 binder-solvent ratio based on Nelson *et al.* (2019).

Table S7: Process inventory for cathode active material mixing for 1 battery pack

Description	Quantity/function	Unit	Inventory
**Input: materials**			
Active material	$cathode\_am_m/\prod_i py_{i,electrode}$	kg	Section 5
Binder (PVDF)	$cathode\_binder/\prod_i py_{i,electrode}$	kg	Section 5.3.1
Binder solvent (NMP) new	$24^a * cathode\_binder/\prod_i py_{i,electrode} * (1 - py_{nmp})^b$	kg	Section 5.3.1
Binder solvent (NMP) recycled	$24^a * cathode\_binder/\prod_i py_{i,electrode} * py_{nmp}{}^b$	kg	Section 5.3.1
Carbon black	$cathode\_cb/\prod_i py_{i,electrode}$	kg	Section 5.3.1
**Input: process**			
Electricity	$0.65 * wt\_cell * electricity_{mixing}/py_{cell}$	kWh	Market for electricity, medium voltage
**Output: reference product**			
Cathode slurry	$\sum cathode_{am,binder,cb,solvent} \prod_i py_{i,electrode}$	kg	
**Output: waste**			
Waste active material	$cathode\_am_m * py_{mix}$	kg	
Waste binder (PVDF)	$cathode\_binder_c mc * py_{mix}$	kg	
waste carbon black	$cathode\_cb * py_{mix}$	kg	

[a] 1:24 binder-solvent ratio based on Nelson *et al.* (2019).
[b] 99.5% recovery rate of NMP.

3.2.2 Inventory electrode coating and drying

After active material mixing, the electrode current collectors are coated with the slurry and dried to fix the coating to the surface of the current collector. During the drying process, the binder solvent is evaporated, whereby only the high value NMP is internally recovered and reused. The assumed NMP recovery rate is 99.5% (Crenna *et al.*, 2021; Nelson *et al.*, 2019). The small fraction of non-recovered NMP is evaporated to the air and modelled as non-methane volatile organic compounds (NMVOC) (Kallitsis *et al.*, 2020; Crenna *et al.*, 2021). Evaporated water is treated as waste flow, similar to Crenna *et al.* (2021). The requirement for the electrode current collector is dependent on the foil thickness and therefore based on the $anode/cathode\_foil$ parameter obtained from the product model.

The energy consumption of cathode drying is typically higher due to the lower drying rate and higher solvent mass (NMP as compared to water) (Jinasena *et al.*, 2021). The modelled values

for the coating process by Jinasena *et al.* (2021) of an NMC333 cathode with an NMP solvent and graphite anode with a water solvent are here used as proxy to determine the ratio between the anode-cathode coating and drying energy consumption. The original energy consumption (both electricity and gas) by Degen & Schütte (2022) are therefore multiplied by this ratio (24% for the anode and 76% for the cathode).

Table S8: Process inventory for anode coating and drying of 1 battery pack

Description	Quantity/function	Unit	Inventory
**Input: materials**			
Anode slurry	wt_anode_slurry	kg	Section 3.2.1
Current collector	wt_anode_foil	kg	Section 5.1.2
**Input: process**			
Electricity	$0.24 * wt\_cell * electricity_{coat}/py_{cell}$	kWh	Market for electricity, medium voltage
Heat	$0.24 * wt\_cell * heat_{coat}/py_{cell}$	MJ	Market group for heat, district or industrial, natural gas[RER]
**Output: reference product**			
Coated anode	$anode\_slurry + anode\_foil - waste$	kg	
**Output: waste**			
Waste water	$anode\_solvent * (1 - py\_am\_mixing)$[a]	m^3	Wastewater, average [Europe w/o CH]
Waste foil	$\mathrm{py}_{foil,coat} * anode\_foil$	kg	Waste handling
Waste slurry	$\mathrm{py}_{slurry,coat} * anode\_slurry$	kg	Waste handling

[a] Accounts for binder yield loss during mixing.

Table S9: Process inventory for cathode coating and drying of 1 battery pack

Description	Quantity/function	Unit	Inventory
**Input: materials**			
Slurry	$wt\_cathode\_slurry$	kg	Section 3.2.1
Current collector	$wt\_cathode\_foil$	kg	Section 5.1.2
**Input: process**			
Electricity	$0.76 * wt\_cell * electricity_{coat}/py_{cell}$	kWh	Market for electricity, medium voltage
Heat	$0.76 * wt\_cell * heat_{coat}/py_{cell}$	MJ	Market group for heat, district or industrial, natural gas [RER]
**Output: reference product**			
Coated cathode	$cathode\_slury + cathode\_foil - waste$	kg	
**Output: waste**			
NMP waste	$cathode\_binder * (py\_am\_mixing)^{a}$	kg	NMP recovery
Waste foil	$\text{py}_{foil,coat} * cathode\_foil$	kg	Waste handling
Waste slurry	$\text{py}_{slurry,coat} * cathode\_slurry$	kg	Waste handling

[a] Accounts for binder lost during mixing.

3.2.3 Inventory calendering and electrode slitting

The dried electrodes are compressed between two calender rollers and slit to the desired size. The calendering process does not require any material inputs or produce waste. In the slitting process, the coated electrodes are slit into strips and into individual electrodes. As the total foil area is larger than the coated area, foil scrap (around 8%) during the process is higher than the coating layer (around 1%) (Nelson *et al.*, 2019). The electricity consumption for the calendering and slitting process are equally divdied between the cathode and anode and only adjusted for cell process yield.

Table S10: Process inventory for cathode/anode electrode slitting

Description	Quantity/function	Unit	Inventory
**Input: materials**			
Coated electrode	$coated\_anode, cathode$	kg	Section 3.2.2
**Input: process**			
Electricity	$0.5 * wt\_cell * electricity_{slit}/py_{cell}$	kWh	Market for electricity, medium voltage
**Output: reference product**			
Slitted electrodes	$coated\_anode, cathode - waste$		
**Output: waste**			
Waste current collector	$cathode, anode\_foil * py_{foil,slitting}$	kg	Waste handling
Waste coated electrode	$coated\_anode, cathode * py_{slurry,slitting}$	kg	Waste handling

3.2.4 Inventory final electrode drying

After slitting, the electrodes are vacuum dried to remove any moisture in the electrodes (Nelson *et al.*, 2019). Energy consumption includes electricity and gas for heating. The final drying process for both anode and cathode is modelled as a single process.

Table S11: Process inventory for final electrode drying

Description	Quantity/function	Unit	Inventory
**Input: materials**			
Slitted anode	$slitted\_anode$	kg	Section 3.2.3
Slitted cathode	$slitted\_cathode$	kg	Section 3.2.3
**Input: process**			
Electricity	$wt\_cell * electricity_{drying}/py\_cell$	kWh	Market for electricity, medium voltage
Heat	$wt\_cell * heat_{drying}/py\_cell$	MJ	Market group for heat, district or industrial, natural gas
**Output: reference product**			
Dried electrode	$slitted\_anode + slitted\_cathode$	kg	

3.2.5 Inventory cell stacking

Cell stacking is the first of four steps in assembling the cell. The required amount of separator is based on the specific separator thickness, optional coating thickness requirements, and the

process yields in cell stacking and the final cell formation. Manufacturing scrap for both the electrodes and separator are included.

Table S12: Process inventory for cell stacking

Description	Quantity/function	Unit	Inventory
**Input: materials**			
Electrode	$wt\_dried\_electrode$	kg	Section 3.2.4
Separator	$wt\_separator/\prod_i py_{i,separator}$	kg	Section 5.3.3
**Input: process**			
Electricity	$wt\_cell * electricity_{winding}/py\_cell$	kWh	Market for electricity, medium voltage
**Output: reference product**			
Jelly roll	$wt\_separator + wt\_electrode - waste$	kg	
**Output: waste**			
Separator waste	$wt\_separator * (1 - py_{separator,stack})$	kg	Waste handling
Anode electrode waste	$wt\_separator * (1 - py_{separator,stack})$	kg	Waste handling
Cathode electrode waste	$wt\_separator * (1 - py_{separator,stack})$	kg	Waste handling

3.2.6 Inventory terminal welding

After stacking, the current collector and tabs are welded together. The tab materials are the same as the current collectors. The weight of the tabs are based on the specific cell design and the final cell yield rate (py_{cell}). No scrap rates during the process are assumed. Energy requirement are also not included due to a lack of data.

Table S13: Process inventory for cell terminal welding

Description	Quantity/function	Unit	Inventory
**Materials**			
Jelly roll	$jelly\_roll$	kg	Section 3.2.5
cell terminal anode	$cell\_terminal\_anode/py_{cell}$	kg	Section 5.3.7
cell terminal cathode	$cell\_terminal\_cathode/py_{cell}$	kg	Section 5.3.7
**Output**			
Welded jelly roll	$jelly\_roll + terminals$	kg	

3.2.7 Inventory enclosing jelly roll

The jelly rolls are enclosed in the cell container.The cell container is assumed to be purchased as finished components and the inventory is described below. Energy requirements and scrap during the enclosing process are not included.

Table S14: Process inventory for cell enclosing

Description	Quantity/function	Unit	Inventory
**Materials**			
Welded jelly rol	$welded\_jelly\_roll$	kg	Section 3.2.6
cell container	$cell\_container/py_{cell}$	kg	Section 5.3.6
**Output**			
Enclosed cell	$welded\_jelly\_roll + cell\_container$	kg	

3.2.8 Inventory electrolyte filling and cell sealing

Electrolyte is added to the enclosed cell and sealed based on crimping, beading or welding methods (for prismatic and cylindrical cells) (Jinasena *et al.*, 2021). The quantity of electrolyte is based on the specific battery design and the process yield during filling and cell formation. Energy consumption for the electrolyte filling and cell sealing are based on the data for cell assembly and washing from Degen & Schütte (2022). In addition, as this is the final process occuring in a dry room environment (dry room processes include part of vacuum drying, cell stacking, welding, cell enclosing and washing), electricity and gas to operate the dehumication unit are allocated to this process.

Table S15: Process inventory for electrolyte filling and cell enclosing

Description	Quantity/function	Unit	Inventory
**Materials**			
Enclosed cell	$enclosed\_cell$	kg	Section 3.2.7
Electrolyte	$electrolyte\ py_{electrolyte,filling} * py_{cell}$	kg	Section 5.3.5
**Input: process**			
Electricity assembly,	$wt\_cell * electricity_{assembly}/py\_cell$	kWh	Market for electricity, medium voltage
Electricity dry room	$wt\_cell * electricity_{dryroom}/py\_cell$	kWh	Market for electricity, medium voltage
Heat dry room	$wt\_cell * heat_{dryroom}/py\_cell$	kWh	Market for electricity, medium voltage
**Output**			
Electrolyte waste	$electrolyte * py_{electrolyte,filling}$	kg	
Unformatted cell	$electrolyte + enclosed\_cell - waste$	kg	

3.2.9 Inventory cell formation

During the formation phase, the final step in the cell assembly, the cell is charged to produce the interface layer (solid electrolyte interface (SEI)) and the functioning of the cell is tested through formation cycling and charge-retentions tests (Pettinger *et al.*, 2018; Nelson *et al.*, 2019). Around 5% of the cells typically fail the quality testing (Duffner *et al.*, 2021; Nelson *et al.*, 2019). Energy consumption is based on the reported values for formation, ageing and testing by Degen & Schütte (2022). Electricity consumption for cell formation and testing is based on the pack capacity rather than the cell weight, assuming all electricity is used for charge and discharge cycling of the cell.

Table S16: Process inventory for cell formation

Description	Quantity/function	Unit	Inventory
**Materials**			
Enclosed cell	$enclosed\_cell$	kg	Section 3.2.7
**Processes**			
Electricity	$cell\_capacity * electricity_{formation}/py_{cell}$	kWh	Market for electricity, medium voltage
Heat	$wt\_cell * heat_{formation}/py_{cell}$	MJ	Market group for heat, district or industrial, natural gas
**Output**			
Waste cell		kg	
Cell	$enclosed\_cell * py_{cell,formation}$	kg	

3.2.10 Inventory module and battery assembly

In the final process step, the accepted cells from the formation phase are assembled in modules and packs (modelled as a single process). An aluminium conduction channel is added to each side of the cell for heat rejection. The cells are then placed into the module packaging, consisting of an air-tight aluminium housing. The size and weight of the module housing is dependent on the specific geometric design features and the thickness of the housing. The module electronics, module terminals, conductors and spacers for gas release are added. Finally, the finished modules are placed into the battery pack jacket, including the battery management system (BMS), the coolant, busbars, module compression plates, pack heaters, interconnects and pack terminals. All these components are assumed to be manufactured outside the battery production factory, and more details for each component are discussed below.

The total electricity consumption for module and battery assembly is 1 kWh/kWh battery (0.115 kWh/kg) based on Sun *et al.* (2020). Electricity requirement for inter-process materials handling as stated by Degen & Schütte (2022) is also allocated to this process to include the overall electricity consumption for the building support systems. To account for all pack

materials (not just the cell), the electricity consumption stated by Degen & Schütte (2022) is multiplied by the total pack weight. No further scrap is assumed to occur at this stage. To account for the construction of the battery factory, an infrastructure process is added in the module and battery assembly process similar to Crenna *et al.* (2021) and Ellingsen *et al.* (2014).

Table S17: Process inventory for module and battery assembly

Description	Quantity/function	Unit	Inventory
**Materials**			
Cell	*cell*	kg	Section 3.2.9
Cell interconnect	*cell_interconnect*	kg	Section 5.4.1
Module container	*module_container*	kg	Section 5.4.2
Module terminals	*module_terminal*	kg	Section 5.4.3
Module tabs	*module_tabs*	kg	Section 5.4.4
Module panels	*module_panels*	kg	Section 5.4.5
Module elastomer pads	*module_elastomer_pads*	kg	Section 5.4.6
Module row rack	*module_rack*	kg	Section 5.4.7
Thermal conductor	*module_thermal_conductor*	kg	Section 5.4.8
Gas release	*gas_release*	kg	Section 5.4.9
Module electronics	*module_electronics*	kg	Section 5.4.10
Pack housing	*battery_jacket*	kg	Section 5.5.1
Management system[a]	*battery_management_system*	kg	Section 5.5.2
Busbar	*busbar*	kg	Section 5.5.3
Module interconnects	*module_interconnects*	kg	Section 5.5.4
Coolant	*coolant*	kg	Section 5.5.5
Cooling tubes	*cooling_mains*	kg	Section 5.5.6
Cooling connectors	*cooling_connectors*	kg	Section 5.5.7
Cooling panels	*cooling_panels*	kg	Section 5.5.8
Pack heating	*pack_heater*	kg	Section 5.5.9
Pack terminal	*pack_terminals*	kg	Section 5.5.10
**Processes**			
Electricity assembly	$battery\_weight * electricity_{assembly}$	kWh	Market for electricity, medium voltage
Electricity Building support systems	$battery\_weight * electricity_{handling}$	kWh	Market for electricity, medium voltage
**Infrastructure**			
Infrastructure	1.41×10^{-9}	unit	Metal working factory construction[RER]
**Output**			
Battery pack	*battery_pack*	unit	

[a] Excludes the module electronics.

4. Supporting information substance flow analysis

Table S1: Chemical elements per battery component. Values are based on reported element quantities in BatPaC or on stoichiometric calculations.

	**Al**	**C**	**Co**	**Cu**	**Fe**	**Li**	**Mn**	**Ni**	**P**	**Si**
SiO	-	-	-	-	-	-	-	-	-	0.467
natural graphite	-	1.000	-	-	-	-	-	-	-	-
anode current collector Cu	-	-	-	1.000	-	-	-	-	-	-
battery jacket Al	1.000	-	-	-	-	-	-	-	-	-
battery jacket Fe	-	-	-	-	1.000	-	-	-	-	-
busbar	-	-	-	1.000	-	-	-	-	-	-
NMC532-LMO	-	-	0.061	-	-	0.058	0.382	0.152	-	-
LFP	-	-	-	-	0.354	0.044	-	-	0.196	-
LMO	-	-	-	-	-	0.043	0.593	-	-	-
NCA	0.014	-	0.092	-	-	0.072	-	0.489	-	-
NMC333	-	-	0.204	-	-	0.072	0.190	0.203	-	-
NMC532-LMO	-	-	0.122	-	-	0.072	0.171	0.304	-	-
NMC622	-	-	0.122	-	-	0.072	0.113	0.363	-	-
NMC811	-	-	0.061	-	-	0.071	0.056	0.483	-	-
cathode current collector Al	1.000	-	-	-	-	-	-	-	-	-
cell container	0.820	-	-	-	-	-	-	-	-	-
cell group interconnect	-	-	-	1.000	-	-	-	-	-	-
cell terminal anode	-	-	-	1.000	-	-	-	-	-	-
cell terminal cathode	1.000	-	-	-	-	-	-	-	-	-
cooling mains Fe	-	-	-	-	1.000	-	-	-	-	-
cooling panels	-	-	-	-	1.000	-	-	-	-	-
electrolyte	-	-	-	-	-	0.006	-	-	-	-
module container	-	-	-	-	1.000	-	-	-	-	-
module interconnects	-	-	-	1.000	-	-	-	-	-	-
module row rack	-	-	-	-	1.000	-	-	-	-	-
module tabs	-	-	-	1.000	-	-	-	-	-	-
module terminal	-	-	-	0.800	-	-	-	-	-	-
module thermal conductor	1.000	-	-	-	-	-	-	-	-	-
pack heater	1.000	-	-	-	-	-	-	-	-	-
pack terminals	-	-	-	0.900	-	-	-	-	-	-

5. Supporting information life cycle assessment

Section 5.1.1 to 5.5.2 provides an overview of the different life cycle inventories used of the LCA modules. The background database is ecoinvent 3.7.1. Transport requirement for processes located in Europe are based on the average European freight transport values as modelled in the ecoinvent 3.7.1. ReCiPe Midpoint (H) V1.13 no LT impact categories are used.

All modules are available in Brightway2 format from the GitHub repository.

5.1 Anode active materials

5.1.1 Inventory anode active materials

Anode active materials include SiO_x (maximum of 10 wt%), synthetic graphite and natural graphite. The natural graphite inventory is based on primary industrial data of battery grade natural graphite production as reported by Engels *et al.* (2022). Matching ecoinvent background inventories are partly based on the carculator model (Sacchi *et al.*, 2022). The inventory is not repeated here.

Synthetic graphite made from pet coke and coal tar based on the GREET inventory as described by Dunn, James, et al. (2015). ecoinvent background inventories are matched to the GREET material inputs and provided in Table S1. The default location for synthetic production is assumed to be in China (Crenna *et al.*, 2021). An ecoinvent process for silicon monoxide is currently not available. Metallurgy grade silicon is instead used as a proxy, similar to Philippot *et al.* (2019).

Table S1: Process inventory for synthetic graphite. Adoption of GREET inventory as described by Dunn *et al.* (2015) with matching ecoinvent inventories similar to Crenna *et al.* (2021)

Description	Quantity	Unit	ecoinvent process
**Materials**			
Petroleum coke	0.96	kg	Market for petroleum coke [GLO]
Coal tar	0.24	kg	Market for coal tar [GLO]
**Processes**			
Heat	5.43	MJ	Market for heat, district or industrial, natural gas [RoW]
Electricity	4.08	kWh	market group for electricity, medium voltage[CN]
**Infrastructure**			
Facility	7.41E-10	unit	Market for chemical factory, organics [GLO]
**Transport**			
Oceanic ship	22	tkm	transport, freight, sea, container ship [GLO]
**Output**			
Synthetic graphite	1	kg	

5.1.2 Inventory anode current collectors

The inventory for the production of 1kg of copper foil is based on Crenna *et al.* (2021). This inventory is based on the general current collector LCI as modelled in most LCA but includes a pre-treament process using sulphuric acid (H_2SO_4) and sodium hydroxide (NaOH) to remove impurities and guarantee resistance to corrosion (Dai *et al.*, 2019*a*). Current collectors are assumed to be produced in Europe. The inventory for a 1 kg current collector is assumed to the same irrespective of foil thickness.

Table S2: Process inventory for anode current collector copper for all thicknesses

Description	Quantity	Unit	ecoinvent process
**Materials**			
Cu	0.72	kg	Market copper, cathode [GLO]
H_2SO_4	0.205	kg	Market for sulfuric acid [RER]
NaOH	0.331	kg	Market for sodium hydroxide, without water, in 50% solution state [GLO]
**Processes**			
Sheet rolling	1	kg	Sheet rolling, copper [RER]
**Infrastructure**			
Facility	4.58E-10	unit	Metal working factory [RER]
**Transport**			
Freight train	0.0438	tkm	Market group for transport, freight train [RER]
Freight lorry	0.1713	tkm	Market for transport, lorry [RER]
Barge	0.0219	tkm	Market for transport, waterways, barge [RER]
**Output**			
Waste[a]	0.536	kg	Market for spend solvent mixture [RER]
Anode current collector (n µm)[b]	1	kg	

[a] Waste of pretreatment (H_2SO_4 and NaOH) based on mass balance (Crenna *et al.*, 2021).
[b] Where n refers the foil thickness including 6-12 µm

5.2 Cathode materials

5.2.1 Inventory layered oxides (NMC & NCA)

Layered oxide active materials include $LiNi_xCo_yMn_zO_2$ and $LiNi_{0.8}Co_{0.15}Al_{0.05}O_2$ (hereafter NMC and NCA oxide). The production of both materials is similar and consists of two main steps: co-precipitation and calcination (Ahmed *et al.*, 2017). In the first steps, metal salts ($CoSO_4$, $NiSO_4$ and $MnSO_4$ for NMC and $NiSO_4$, $CoSO_4$ and $Al_2(SO_4)_3$ for NCA) are reacted with either sodium carbonate (Na_2CO_3) or hydroxide (NaOH) and ammonium hydroxide (NH_4OH) to produce a precursor material. In the second step, the precursor is mixed and calcinated with lithium carbonate (Li_2CO_3) or Li hydroxide (LiOH) in the case of NMC811 to produce the oxide.

In the LCA literature, the most commonly used inventory for the production of NMC oxide is derived from Majeau-Bettez *et al.* (2011*a*). However, this LCI is based on a lab scale production process (Liu *et al.*, 1999; Ngala *et al.*, 2004) which was combined with additional calculations and

assumptions. Instead the updated GREET inventory for NMC materials is used here (Dai *et al.*, 2018*a*), which is based on primary data from a Chinese NMC material producer. Material inputs and quantities for NCNM 333, 622 and 811 are obtained from Dai *et al.* (2019*b*) and NMC532 from Winjobi *et al.* (2020), which is an stoichiometrically calculated inventory based on Dai *et al.* (2018*b*). Matching ecoinvent background inventories are all based on Crenna *et al.* (2021), who previously linked the GREET inventories to ecoinvent proxies.

In the co-precipitation process, NAOH and NH_4OH are added to the sulfate solution. Excess ammonia is removed from the waste water in a stripping tower and reused within the precursor production (Dai *et al.*, 2018*a*). To account for the closed-loop recycling of ammonia, only the losses of NH_3 after the stripping process are accounted (10% loss and 0.486 kg NH_3 required per kg of NH_4OH) for based on Crenna *et al.* (2021). The co-precipitation and calcination process takes place in two different facilities (Dai *et al.*, 2018*a*), and modelled as two separate inventories. Transport between the two facilities is not accounted for. The required infrastructure is based on a 27,000 ton production output and assuming a 50yr lifetime of the chemical factory. All input requirements for the precursor production of all NMC types are the same with the exception of the Ni, Co, and Mn sulfates as highlighted in table S3. In addition, LiOH is used for NMC811 instead of Li_2CO_3 in the oxide production step.

Table S3: Process inventory for NMC333/532/622/811 precursor production

Description	Quantity	Unit	ecoinvent process
**Materials**			
$NiSO_4$	0.56/0.87/ 1.01/1.34	kg	Market for nickel sulfate [GLO]
$CoSO_4$	0.56/0.35/ 0.34/0.17	kg	See Section 5.2.4
$MnSO_4$	/0.55/0.49/ 0.33/0.16	kg	Market for manganese sulfate [GLO]
NH_4	6.05×10^{-3}	kg	Market for ammonia, anhydrous, liquid [GLO]
NaOH	0.88	kg	Market for sodium hydroxide, without water, in 50% solution state [GLO]
Cooling water	6.38×10^{-4}	m^3	Water, cooling, unspecified natural origin
**Processes**			
Heat[a]	40.74	MJ	Market for heat, district or industrial, natural gas [RoW]
**Infrastructure**			
Facility	7.41E-10	unit	Market for chemical factory, organics [GLO]
**Output**			
Waste[b]	1.57/1.6/ 1.56/1.52	kg	Market for sodium sulfate, anhydrite [RoW]
Waste water	6.38×10^{-4}	m^3	Wastewater average
Ammonia	6.05×10^{-2}	kg	Ammonia, to air
Waste water	6×10^{-4}	m^3	Market for wastewater, average
NMC333/532/622	1	kg	

[a] 38.618 mmBTU/tonne of NMC precursor

[b] Waste sodium sulfate, mass balance based on Crenna *et al.* (2021).

Table S4: Process inventory for NMC333/532/622/811 active material production and import

Description	Quantity	Unit	ecoinvent process
**Materials**			
LiCO	0.38	kg	Market for lithium carbonate [GLO]
LiOH[a]	0.25	kg	Market for lithium hydroxide [GLO]
NMC precursor[b]	0.95	kg	Table S3
**Processes**			
Electricity	6.87	kWh	Market group for electricity, medium voltage [CN]
**Infrastructure**			
Facility	7.4×10^{-10}	unit	Market for chemical factory, organics [GLO]
**Transport**			
Oceanic ship	22	tkm	transport, freight, sea, container ship [GLO]
**Output**			
Carbon dioxide [c]	0.21	kg	Carbon dioxide, fossil, to air
Water evaporation[d]	0.13/ 1.96×10^{-4}	m^3	Water to air
Active material (NMC_{xyz})	1	kg	

[a] Lithium hydroxide only for NMC811.
[b] NMC333/532/622 or $811(OH)_2$ respectively.
[c] CO_2 emissions from thermal decomposition of LiCO. Therefore only applicable for NMC333, 532 and 622.
[d] Based on mass balance Crenna *et al.* (2021). 0.13 m^3 for NMC333, 532 and 622, 1.96×10^{-4} for NMC811.

The NCA inventory is based on the GREET2 as described by Benavides *et al.* (2015) with an updated energy and water requirement as discussed in Dai *et al.* (2018*a*). Similar to NMC production, NCA is produced in two main steps. First, metal salts (Al, Co and Ni) are mixed and precipitated to obtain a NCA hydroxide ($NCA(OH)_2$) intermediate product. The molar ratio of the NCA active material is 0.80:0.15:0.05 based on Benavides *et al.* (2015) and in line with the NCA material in BatPaC. In the second process step, the $NCA(OH)_2$ is mixed with a lithium source (assuming LiOH as discussed above), roasted, crushed, washed and dried to obtain the NCA active material. Similar to the NMC production inventory, it is assumed that these two production processes (precursor production and active material production) take place in two different facilities. Matching background ecoinvent inventories are based on (Crenna *et al.*, 2021) as well as the facility requirements and waste products (Table S5 and S6). Production takes place in China and is shipped to Europe.

Table S5: Process inventory for NCA precursor production

Description	Quantity	Unit	ecoinvent process
**Materials**			
$NiSO_4$	1.36	kg	Market for nickel sulfate [GLO]
$CoSO_4$	0.26	kg	See Section 5.2.4
$AlSO_4$	0.09	kg	Market for aluminium sulfate, powder [RoW]
NH_4	1.81×10^{-2}	kg	Market for ammonia, anhydrous, liquid [CN]
NaOH	0.89	kg	Market for sodium hydroxide, without water, in 50% solution state [GLO]
Cooling water	6.38×10^{-4}	m^3	Water, cooling, unspecified natural origin
**Processes**			
Heat	40.74	MJ	Market for heat, district or industrial, natural gas [RoW]
**Infrastructure**			
Facility	7.41E-10	unit	Market for chemical factory, organics [GLO]
**Output**			
Ammonia	1.81×10^{-2}	kg	Ammonia, to air
Waster water	6.38×10^{-4}	m^3	Market for wastewater, average
Waste[a]	1.59	kg	Market for sodium sulfate, anhydrite [RoW]
NCA	1	kg	

[a] Waste sodium sulfate, mass balance based on Crenna *et al.* (2021).

Table S6: Process inventory for NCA material production and import

Description	Quantity	Unit	ecoinvent process
**Materials**			
LiOH	0.25	kg	Market for lithium hydroxide [GLO]
NCA precursor	0.95	kg	Table S5
**Processes**			
Electricity	7.26	kWh	Market group for electricity, medium voltage [CN]
**Infrastructure**			
Facility	7.4×10^{-10}	unit	Market for chemical factory, organics [GLO]
**Transport**			
Oceanic ship	22	tkm	transport, freight, sea, container ship [GLO]
**Output**			
Water evaporation	2.4×10^{-4}/ 1.96×10^{-4}	m^3	Water to air
Active material (NCA)	1	kg	
Oxygen	0.04	kg	Oxygen, natural resource, in air

5.2.2 Inventory spinel oxide (LMO)

Different LMO production routes exist which can be classified in four main categories: solid state, sol-gel, hydro thermal and combustion method. Large scale production of LMO through the hydro thermal and combustion routes are challenging due the difficulty of controlling the production process and the low yields through the hydro thermal route (Susarla & Ahmed, 2020).

In the ecoinvent database, an inventory for an LMO production process based oon the solid state method is available. This dataset is based on the inventory of LMO production obtained by (Notter *et al.*, 2010) and is also used in GREET (Dunn *et al.*, 2014). Not much information about the process is available in the original publication from Notter *et al.* (2010) but the origin of this dataset is primarily based on a LMO production patent from Germany (EPA No. EP1204601) (Heil *et al.*, 2003). Here LMO is made from Mn and Li compounds, (preferably Mn_2O_3 and Li_2CO_3) through several roasting stages.

A more recent inventory, including cost and energy estimations, for LMO production is presented by Susarla & Ahmed (2020) where both the solid state and sol-gel method are presented. The solid state process inventory by Susarla & Ahmed (2020) is comparable to Notter *et al.* (2010) but provides more details and different assumptions (see Table S7 for an overview).

First, the Mn compound in the process by Susarla & Ahmed (2020) is based on electrolytic Mn dioxide (EMD) compared to an Mn oxide (Mn2O3) by Notter *et al.* (2010), whereby the former is more widely used as starting material (Biswal *et al.*, 2015; Lee *et al.*, 2012). Second, the

process inventory by Susarla & Ahmed (2020) includes a significantly higher water consumption. This can be explained by the need for washing of the EMD to remove any trace amounts of metals, which also requires sulfuric acid (H_2SO_4). The higher amount of water requirements also translates into the higher electricity requirement for pumping power. In the process inventory by Notter *et al.* (2010), electricity is only required for the mechanical drive of the rotary kiln, resulting in a lower overall electricity demand. Finally, Susarla & Ahmed (2020) has a higher heat demand. Although the obtained values of heat demand by Notter *et al.* (2010) are not clear, the higher heat demand could be partly by the longer calcination time that is assumed.

Table S7: Comparison of inventories for the production of 1kg $LiMn_2O_2$.

**Input**	**unit**	**Susarla & Ahmed (2020)[a]**	**Notter *et al.* (2010)**
Mn_2O_3	kg	-	0.918
MnO_2	kg	1.017	-
LiCO	kg	0.217	0.215
H_2SO_4	kg	0.017	
Water	kg	24.88	3.4
Electricity	kWh	0.079	0.005
Heat	MJ	22.2	15.3
N_2	kg	-	0.786
O_2	kg	-	0.715
**Output**			
CO_2	kg	-	0.128
LMO	kg	1	1

[a] Inventory is based on the solid state process.

To include the LMO production process route based on EMD and the higher energy demand, the inventory by Susarla & Ahmed (2020) is used here. Most matching ecoinvent background inventories for the required materials are based on Notter *et al.* (2010) with updated values from Susarla & Ahmed (2020). For the Mn_2O_3, the ecoinvent for manganese dioxide production is chosen as proxy. It should be noted that this inventory is based on the chemical route of Mn_2O_3 production (chemical manganese dioxide (CMD)) rather than the electrochemical route (EMD).

98% of the H_2SO_4, used to remove any metals present in the EMD, is recovered and assumed to be reused for a second purpose off-site (Susarla & Ahmed, 2020). Non-fuel carbon dioxide emissions as a result of the LiCO+MnO_2 calcination are based on stoichiometric calculation (1 mol CO2 per 2 mol LMO) following a similar procedure by Dunn *et al.* (2014) for the GREET LMO inventory. Similar to the spinel oxide active materials, production is assumed to take place in China. The final inventory can be found in Table S8.

Table S8: Process inventory for LMO production

Description	Quantity	Unit	ecoinvent process
**Materials**			
Mn_2O_3	1.02	kg	Market for manganese dioxide[GLO]
LiCO	0.217	kg	Market for lithium carbonate [GLO]
H_2SO_4	0.017	kg	Market for sulfuric acid [RoW]
Water	24.88	kg	Market for water, deionised [ROW]
**Processes**			
Electricity	0.079	kWh	Market group for electricity, medium voltage [CN]
Heat	22	MJ	Market for heat, district or industrial, natural gas [RoW]
**Infrastructure**			
Facility	7.4×10^{-10}	unit	Market for chemical factory, organics [GLO]
**Transport**			
Oceanic ship	22	tkm	transport, freight, sea, container ship [GLO]
**Output**			
CO_2	.122	kg	Carbon dioxide, fossil
H_2SO_4	0.0166	kg	Market for sulfuric acid [RoW]
Active material (LMO)	1	kg	

5.2.3 Inventory polyanion oxide (LFP)

LiFeP4 (LFP) active material powder production methods can be categorised in solid state and solution based methods, and several synthesis routes exist (Satyavani *et al.*, 2016). The LFP production inventory by Majeau-Bettez *et al.* (2011*b*) is adopted here as recommend by Peters & Weil (2018). This inventory is based on the solution based method (a hydro thermal process route) as described by Chen & Whittingham (2006). Here iron sulfate ($FeSO_4$) (a byproduct from the iron industry) is reacted with phosphoric acid (H_3PO_4) and LiOH. Similar to the spinel oxide active materials, production is assumed to take place in China. The inventory is highlighted in Table S9.

Table S9: Process inventory for LFP production

**Description**	**Quantity**	**Unit**	**ecoinvent process**
**Materials**			
$FeSO_4$	1	kg	Market for iron sulfate [RoW]
LiOH	0.46	kg	Market for lithium hydroxide [GLO]
H_3PO_4	0.65	kg	Market for phosphoric acid, industrial grade [GLO]
Water	46	kg	Market for water, deionised [ROW]
**Processes**			
Heat	15	MJ	Market for heat, district or industrial, natural gas [RoW]
**Infrastructure**			
Facility	7.4×10^{-10}	unit	Market for chemical factory, organics [GLO]
**Transport**			
Oceanic ship	22	tkm	transport, freight, sea, container ship [GLO]
**Output**			
Iron, ion	0.019	kg	Iron ion, to water
Lithium, ion	0.1	kg	Lithium ion, to water
Waste heat	1.5	MJ	Heat waste, to air
Active material (LFP)	1	kg	

5.2.4 Inventory cobalt sulfate

$CoSO_4$ production consist of three major steps, including mining, processing of ore into Co hydroxide ($Co(OH)_2$) and conversion of hydroxide into $CoSO_4$ (Dai *et al.*, 2018*b*). Currently, no ecoinvent background inventory for $CoSO_4$ is present, but an inventory for $Co(OH)_2$ based on inventory data from the Cobalt Institute does exist. The $CoSO_4$ inventory from GREET is therefore used (Dai *et al.*, 2018*b*). The matching ecoinvent background processes and quantities based on economic allocation are obtained from Crenna *et al.* (2021) who, indirectly, use

this same inventory to model the production of $CoSO_4$. Similar to Dai *et al.* (2018*b*), $CoSO_4$ production is assumed to take place in China, the main $Co(OH)_2$ and $CoSO_4$ producer in the world (Baars *et al.*, 2021).

5.2.5 Inventory cathode current collector

This inventory is based on Crenna *et al.* (2021). Current collectors are assumed to be produced in Europe. The inventory for a 1 kg current collector is assumed to the same irrespective of foil thickness.

Table S10: Process inventory for cathode current collector aluminium for all thicknesses

Description	Quantity	Unit	ecoinvent process
**Materials**			
Al	0.72	kg	Market for aluminium, wrought alloy [GLO]
H_2SO_4	0.205	kg	Market for sulfuric acid [RER]
NaOH	0.331	kg	Market for sodium hydroxide, without water, in 50% solution state [GLO]
**Processes**			
Sheet rolling	1	kg	Sheet rolling, aluminium [RER]
**Infrastructure**			
Facility	1.5×10^{-10}	unit	Aluminium casting facility construction [RER]
**Transport**			
Freight train	0.0438	tkm	Market group for transport, freight train [RER]
Freight lorry	0.1713	tkm	Market for transport, lorry [RER]
Barge	0.0219	tkm	Market for transport, waterways, barge [RER]
**Output**			
Waste[a]	0.536	kg	Market for spend solvent mixture [RER]
Cathode current collector Al (*n* µm)[b]	1	kg	

[a] Waste of pretreatment (H_2SO_4 and NaOH) based on mass balance (Crenna *et al.*, 2021).
[b] Where *n* refers the foil thickness including 10-16 µm

5.3 Cell materials other

5.3.1 Inventory electrode binders, additives and solvents

Currently, a process inventory for PVDF, the cathode binder, is not present in the ecoinvent database or literature Peters & Weil (2018). The ecoinvent polyvinylfluoride (PVF) inventory is therefore used as a proxy, commonly applied in the literature (e.g. Ellingsen *et al.* (2016); Notter *et al.* (2010)). N-methyl-2-pyrrolidone (NMP) is used as solvent for PVDF binders, as commonly assumed in the LCA literature. A corresponding inventory for NMP is present in ecoinvent. For the water based binder used in the anode, CMC, the ecoinvent inventory carboxymethyl cellulose,

is used, similar to Peters *et al.* (2016); Crenna *et al.* (2021). No ecoinvent inventory for SBR, the CMC additive, is currently present. The SBR inventory by Peters *et al.* (2016) is therefore used to model the production of SBR (see Table S11).

Table S11: Process inventory of styrene-butadiene-rubber (SBR) based on (Peters *et al.*, 2016)

Description	Quantity	Unit	ecoinvent process
**Materials**			
Butadiene	0.72	kg	Market for butadiene [RER]
Styrene	0.25	kg	Market for styrene [GLO]
Emulsifier	0.03	kg	Market for soap [GLO]
Water	1.8	kg	Market for water, deionised [Europe w/o CH]
Solvent	0.01	kg	Cyclohexane production [RER]
Initiator	5.04×10^{-3}	kg	Market for sodium persulfate [GLO]
**Processes**			
Electricity	0.55	kWh	Market for electricity, medium voltage [RER]
Heat	13.75	MJ	Market for heat, central or small-scale, natural gas[Europe w/o CH]
**Infrastructure**			
Facility	4×10^{-10}	unit	Market for chemical factory, organics [GLO]
**Transport**			
Freight train	0.0438	tkm	Market group for transport, freight train [RER]
Freight lorry	0.1713	tkm	Market for transport, lorry [RER]
Barge	0.0219	tkm	Market for transport, waterways, barge [RER]
**Output**			
NMVOC	0.014	kg	NMOVC, unspecified origin
Waste heat	15.73	MJ	Heat, waste, to air
Wastewater	1.8	L	wastewater, unpolluted
SBR	1	kg	

[a] NMC/NCA, LMO and LFP electrolyte are assumed to be produced in a similar way due to the lack of data.

5.3.2 Inventory carbon black

The ecoinvent inventory 'Market for carbon black [GLO]' is used for both the anode (if present) and cathode carbon black.

5.3.3 Inventory separator

All teardown report highlight the presence of a coating layer on the separator material such as Al_20_3. Coating layers on the foil materials are not commonly included in LCA despite the presence of a coated separator ecoinvent background inventory based on the study by Notter *et al.* (2010). The inclusion of a ceramic coating rather than a plain polyolefin material results in higher environmental emissions (Peters & Weil, 2018; Crenna *et al.*, 2021).

The inventory for non-coated separators is based on Ellingsen *et al.* (2014), where granulated PP is used as the input material and injection moulding as the production process (Table S12). The production process for the different separator thicknesses is assumed to be the same.

Table S12: Process inventory of non-coated PP separator based on Ellingsen *et al.* (2014)

Description	Quantity	Unit	ecoinvent process
**Materials**			
Polypropylene	1	kg	Market for polypropylene, granulate [GLO]
**Processes**			
Production	1	kg	Injection moulding [RER]
**Infrastructure**			
Facility	7.4×10^{-10}	unit	Plastic processing factory construction [RER]
**Transport**			
Freight train	0.0438	tkm	Market group for transport, freight train [RER]
Freight lorry	0.1713	tkm	Market for transport, lorry [RER]
Barge	0.0219	tkm	Market for transport, waterways, barge [RER]
**Output**			
Separator (5/7/9 µm) [a]	1	kg	

[a] Production processes for 5, 7 and 9 µm non-coated separators are assumed to be the same.

5.3.4 Inventory coated separator

The inventory of the coated separator is based on Notter *et al.* (2010) with two modification. First, the PE material (originally modelled as a PE fleece) is substituted for a PP membrane by including polypropylene as material and injection moulding as an additional process, similar to Crenna *et al.* (2021). Second, the PP:coating ratio is adjusted according to the three different separator thicknesses included in the model. The weight of the PP and coating material (wt_{sep_m}) required for the production of 1 kg coated separator is calculated with Equation 5.1.

$$wt_{sep\_m} = \frac{th_{sep\_m}\rho_{sep\_m}V sep}{th_{sep}\rho_{sep}} \tag{5.1}$$

As discussed in Section 2, the densities (ρ) are 1.996 g/cm3 and 0.9 for the coating and PP layer, respectively. ρ_{sep} refer to the total thickness and weight of the separator as calculated with Equation 2.4 and th_{sep} to the total thickness. V_{sep} refers to the porosity of the separator, which is 50% based on BatPaC.

Based on this, the inventory for coated separators is presented in Table S13. The quantities of PVDF (26 wt.%), hexafluorpropylene (4 wt.%),dibutyl phthalate (DBP,40 wt.%) and silica (30 wt.%) in the original inventory by Notter *et al.* (2010) are adjusted based on the weight of the coating. Process energy (heat for solvent evaporation and electricity for mechanical drive

processes) and the scrap rate (5%) is assumed to remain the same.

Table S13: Process inventory for three different coated separators thickness types (5/7/9 µm 2/2/3µm)

Description	Quantity	Unit	ecoinvent process
**Materials**			
Polypropylene	0.56/0.64/0.6	kg	Market for polypropylene, granulate [GLO]
Acetone	0.014	kg	Market for aceton, liquid [GLO]
Waste	0.05	kg	Market for residue from shredder fraction from manual dismantling [CH]
Hexaflouroethane	0.02/0.01/0.02	kg	Market for hexafluoroethane [GLO]
PVDF	0.13/0.11/0.12	kg	Market for polyvinylfluoride [GLO] (proxy for PVDF)
Silica	0.15/0.12/0.13	kg	Market for silica sand [GLO]
DBP	0.2/0.16/0.18	kg	Market for phtalic anhydride (proxy for DBP)
**Processes**			
Production	0.56/0.64/0.6	kg	Injection moulding [RER]
**Infrastructure**			
Facility	4×10^{-10}	unit	Market for chemical factory, organics [GLO]
**Transport**			
Freight train	0.0438	tkm	Market group for transport, freight train [RER]
Freight lorry	0.1713	tkm	Market for transport, lorry [RER]
Barge	0.0219	tkm	Market for transport, waterways, barge [RER]
**Output**			
Coated separator (5/7/9µm+2/2/3µm)	1	kg	

5.3.5 Inventory electrolyte

Liquid electrolyte consists of an organic solution consisting of conducting lithium salts, carbonates as solvents and additives (Armand *et al.*, 2020*b*). Lithium hexafluorophosphate (LiPF6) as salts and ethylene carbonate (EC) and dimethyl carbonate (DMC) as solvents are most commonly used according to teardown reports and the literature (see table S14). Small amounts of additives (¡5wt%) are used to reduce the performance (cycleability and cycle life) and safety of LiB (Zhang, 2006). The most common additive is vinylene carbonate (VC) (Kwade *et al.*, 2018; Armand *et al.*, 2020*a*).

The electrolyte is assumed to purchased by battery producers as a pre-mixed product consisting of $LiPF_6$ as Li salt, EC and DMC as solvents and VC as additive. 1.2 mole of $LiPF_6$ is assumed to be solved in EC:DMC with a ratio of 70:30 and 2% VC additive as common practise (Sun *et al.*, 2020; Majeau-Bettez *et al.*, 2011*a*; Peters & Weil, 2018; Greenwood *et al.*, 2021; Crenna *et al.*, 2021).

Different process inventory background data proxies are used to model primary $LiPF_6$ pro-

duction (Peters & Weil, 2018). The $LiPF_6$ inventory in ecoinvent is used as is recommended by Peters & Weil (2018). This inventory is based on Notter *et al.* (2010) and described in more detail there. Here it is assumed that the electrolyte is produced in China and transported to Europe. The rest of the inventory is primarily based on Crenna *et al.* (2021) to obtain the matching ecoinvent material, electricity and process background inventories but with a different EC:DMC ratio (30:70). The VC additive is not available in ecoinvent but modelled according to Crenna *et al.* (2021). The inventory is not repeated here.

Table S14: Process inventory of elecrolyte production

**Description**	**Quantity**	**Unit**	**ecoinvent process**
**Materials**			
$LiPF_6$%	0.126	kg	Market for lithium hexafluorophosphate [GLO]
EC	0.258	kg	Market for ethylene carbonate [GLO]
DMC	0.602	kg	Market for dimethyl carbonate [GLO]
VC	0.024	kg	See Crenna *et al.* (2021)
**Processes**			
Electricity	0.416	kWh	Market for electricity, medium voltage [CN]
**Infrastructure**			
Facility	4×10^{-10}	unit	Market for chemical factory, organics [GLO]
**Transport**			
Oceanic ship	22	tkm	transport, freight, sea, container ship [GLO]
**Output**			
Electrolyte[a]	1	kg	

[a] NMC/NCA, LMO and LFP electrolyte are assumed to be produced similarly due to the lack of data.

5.3.6 Inventory cell container

The inventory used for the production of 1 kg of cell container is described in Table S15. The ecoinvent background inventories for materials and processes is based on Crenna *et al.* (2021), who match ecoinvent inventories to the description of container production by Dai *et al.* (2019a) which again is based on BatPaC. The infrastructure input is based on Ellingsen *et al.* (2014), and transportation service based on European averages. The quantities of each material (PET, Al, PP) is determined by the battery design model.

Table S15: Process inventory of cell container production

Description	Quantity/ function	Unit	ecoinvent process
**Materials**			
PET	*cell_cont_pet/cell_cont*	kg	Market for polyethylene terephthalate [GLO]
PP	*cell_cont_pp/cell_cont*	kg	Market for polypropylene, granulate [GLO]
Al	*cell_cont_al/cell_cont*	kg	Market for aluminium, wrought alloy [GLO]
**Processes**			
Extrusion	*cell_cont_pet + pp/cell_cont*	kg	Extrusion, plastic film [RER]
Sheet rolling	*cell_cont_al/cell_cont*	kg	Sheet rolling, aluminium [RER]
**Infrastructure**			
Facility plastics		unit	Plastic processing factory [RER]
Facility Al	7.7×10^{-11}	unit	Aluminium casting plant [RER]
**Transport**			
Freight train	0.0438	tkm	Market group for transport, freight train [RER]
Freight lorry	0.1713	tkm	Market for transport, lorry [RER]
Barge	0.0219	tkm	Market for transport, inland waterways, barge [RER]
**Output**			
Cell container	1	kg	

5.3.7 Inventory cell terminal

The cell terminals are made out of a thin Cu (anode) and Al (cathode) layer on both sides of the cell and cover almost the entire width of the cell (Nelson *et al.*, 2019). The required weight of each terminal is calculated in BatPaC and based on the cell geometry and thickness of the terminal material (default value is 1mm). The inventory for Al and Cu terminals are based on Ellingsen *et al.* (2014) and Crenna *et al.* (2021), assuming production takes place in Europe.

Table S16: Process inventory of cell terminal (cathode)

Description	Quantity/ function	Unit	ecoinvent process
**Materials**			
Al	1	kg	Market for aluminium, wrought alloy [GLO]
**Processes**			
Sheet rolling	1	kg	Sheet rolling, aluminium [RER]
**Infrastructure**			
Facility	1.5×10^{-10}	unit	Aluminium casting plant [RER]
**Transport**			
Freight train	0.0438	tkm	Market group for transport, freight train [RER]
Freight lorry	0.1713	tkm	Market for transport, lorry [RER]
Barge	0.0219	tkm	Market for transport, inland waterways, barge [RER]
**Output**			
Cell terminal cathode	1	kg	

Table S17: Process inventory of cell terminal (anode)

Description	Quantity/ function	Unit	ecoinvent process
**Materials**			
Cu	1	kg	Market copper, cathode [GLO]
**Processes**			
Sheet rolling	1	kg	Sheet rolling, copper [RER]
**Infrastructure**			
Facility	4.6×10^{-10}	unit	Metal working factory [RER]
**Transport**			
Freight train	0.0438	tkm	Market group for transport, freight train [RER]
Freight lorry	0.1713	tkm	Market for transport, lorry [RER]
Barge	0.0219	tkm	Market for transport, inland waterways, barge [RER]
**Output**			
Cell terminal anode	1	kg	

5.4 Module materials

5.4.1 Inventory cell group interconnect

If the module consists of more than one row of cells, a Cu cell group interconnect is included. The weight of the cell group interconnect is based on BatPaC. In BatPaC, the interconnect material is the same as the positive cell terminal. The inventory for the cell group interconnect is therefore the same as the negative cell terminal, see Table S17.

5.4.2 Inventory module container

The module container in BatPaC consist of a thin (default 0.3 mm) steel sheet. Steel steel rolling is therefore used a proxy to produces this product. Similar to the battery pack jacket, a deep drawing process is also included (see Section 5.5.1).

Table S18: Process inventory of module container production for liquid cooled system

Description	Quantity/ function	Unit	ecoinvent process
**Materials**			
Steel	1	kg	Market for steel, low-alloyed [GLO]
**Processes**			
Sheet rolling	1	kg	Sheet rolling, steel [RER]
Steel shaping	1	kg	Deep drawing, steel, 10000 kN press, single stroke [RER]
**Transport**			
Freight train	0.0438	tkm	Market group for transport, freight train [RER]
Freight lorry	0.1713	tkm	Market for transport, lorry [RER]
Barge	0.0219	tkm	Market for transport, inland waterways, barge [RER]
**Output**			
Module container	1	kg	

5.4.3 Inventory module terminals

The module terminal connectors in BatPaC are modelled as two 35 mm long conductors made out of 80% copper and 20% insulation and fasteners. The ecoinvent background inventories are based on Crenna *et al.* (2021) for the plastic insulation and infrastructure and the Cu conductor is based on Ellingsen *et al.* (2014), with an update for the Cu material based on ecoinvent 3.7.1. Production is assumed to be in Europe and an average transportation services for Europe is included.

Table S19: Process inventory of module terminals

Description	Quantity	Unit	ecoinvent process
**Materials**			
Cu	0.8	kg	Market for copper, cathode [GLO]
PE	0.2	kg	glass fibre reinforced plastic production [RER]
**Processes**			
Cu conductor	0.8	unit	Metal working, average copper product [RER]
**Infrastructure**			
Facility	4.85E-10	unit	Metal working factory construction [RER]
**Transport**			
Freight train	0.0438	tkm	Market group for transport, freight train [RER]
Freight lorry	0.1713	tkm	Market for transport, lorry [RER]
Barge	0.0219	tkm	Market for transport, inland waterways, barge [RER]
**Output**			
Module terminal	1	kg	

5.4.4 Inventory module tabs

Tabs to module terminals consisting of 100% copper as stated in BatPaC. The same inventory as the cell anode terminal is used for this process. See also Table S17.

5.4.5 Inventory module interconnect panels

As stated in BatPaC, the module interconnect panels (front and back) are made out of PP with a default thickness of 2mm. The ecoinvent background inventory “Market for polypropylen [GLO]” is used as a proxy for this process.

5.4.6 Inventory module elastomer pads

Polymer pads between modules in BatPaC have a default thickness of 2 mm and are made out of elastomer. The ecoinvent inventory “market for synthetic rubber [GLO]” is used as a proxy based on the battery retention foams as used by Ellingsen *et al.* (2014).

5.4.7 Inventory module row rack

The module row rack in BatPaC consist of three elements: the lower channel, upper and vertical rack and module restrain plates. All elements are made out of steel. The size is fully customisable in BatPaC and depends on several parameters such as thickness of channels or thickness of module restrain plates. The ecoinvent inventory proxies are based on the steel battery retention system as modelled by Ellingsen *et al.* (2014) with production located in Europe.

Table S20: Process inventory for module row rack

Description	Quantity	Unit	ecoinvent process
**Materials**			
Steel	1	kg	market for steel, low-alloyed [GLO]
**Processes**			
Production	1	kg	Sheet rolling, steel [RER]
**Infrastructure**			
Facility	4.4E-10	unit	Metal working factory construction [RER]
**Transport**			
Freight train	0.044	tkm	Market group for transport, freight train [RER]
Freight lorry	0.171	tkm	Market for transport, lorry [RER]
Barge	0.022	tkm	Market for transport, inland waterways, barge [RER]
**Output**			
Cooling tubes	1	kg	

5.4.8 Inventory module thermal conductors

An aluminium heat conduction channel in the module transfers the cell heat to the cooled walls of the module (Nelson *et al.*, 2019). The weight of heat conductor is based on the cell and module geometry and determined by the specific battery design in BatPaC. The ecoinvent background inventories are based on Crenna *et al.* (2021). Production is assumed to be in Europe.

Table S21: Process inventory of module thermal conductors

Description	Quantity/ function	Unit	ecoinvent process
**Materials**			
Al	1	kg	Aluminium, wrought alloy [RER]
**Processes**			
Production	1	kg	Sheet rolling, aluminium [RER]
**Infrastructure**			
Facility	4.85E-10	unit	Metal working factory construction [RER]
**Transport**			
Freight train	0.0438	tkm	Market group for transport, freight train [RER]
Freight lorry	0.1713	tkm	Market for transport, lorry [RER]
Barge	0.0219	tkm	Market for transport, inland waterways, barge [RER]
**Output**			
Module thermal conductor	1	kg	

5.4.9 Inventory polymer spacers gas release

The gas release system in the module allows for the release of excessive gas through pressure release disks at the back of the module (Nelson *et al.*, 2019). Polymer spacers with a default lenght of 6mm are added to allow for the gas passage between the cell terminals and back of the module. The ecoinvent background inventories are based on Crenna *et al.* (2021). Production is assumed to be in Europe and an average transportation services for Europe is included.

Table S22: Process inventory of module polymer spacers gas release

Description	Quantity	Unit	ecoinvent process
**Materials**			
PE	1	kg	Market for polyethylene, granulate [RER]
**Processes**			
Production	1	kg	Extrusion, plastic film [RER]
**Infrastructure**			
Facility	7.4E-10	unit	Plastic processing factory construction [RER]
**Transport**			
Freight train	0.044	tkm	Market group for transport, freight train [RER]
Freight lorry	0.171	tkm	Market for transport, lorry [RER]
Barge	0.022	tkm	Market for transport, inland waterways [RER]
**Output**			
Module gas release	1	kg	

5.4.10 Inventory module electronics

The electronics in the module includes a state-of-charge (SOC) regulator and cell monitoring system for malfunctions (Nelson *et al.*, 2019). The weight of the module electronics system is calculated within the BatPaC model based on user defined parameters including number of cells and number of cells in parallel. The inventory of the module electronics is based on a single ecoinvent market process for printed wiring boards based on Ellingsen *et al.* (2014): Market for printed wiring board, through-hole mounted, unspecified, Pb free [GLO].

5.5 Battery pack materials

5.5.1 Inventory battery jacket

The battery pack jacket as modelled in BatPaC contains Al, Fe and insulation materials. The mass of each material is based on their respective thickness, which can be changed in BatPaC. ecoinvent background inventory for Fe, Al, sheet rolling and extrusion are based on Crenna *et al.* (2021) and Ellingsen *et al.* (2014). The material used for insulation is not disclosed in BatPaC. A polypropylene layer is instead used as a proxy based on commonly used EV battery insulation materials, as highlighted by MARIAN (2019). Crenna *et al.* (2021) suggests that the best proxy for the process of shaping battery jacket is sheet rolling and deep drawing. As a deep drawing process in ecoinvent is only available for Fe, an impact extrusion process is used for Al as suggested by Crenna *et al.* (2021).

Table S23: Process inventory of battery jacket

Description	Quantity/ function	Unit	ecoinvent process
**Materials**			
Aluminium	*pack_cont_al/pack_cont*	kg	Market for aluminium, wrought alloy [GLO]
Steel	*pack_cont_fe/pack_cont*	kg	Market for steel, low-alloyed [GLO]
Insulation	*pack_cont_pp/pack_cont*	kg	Market for polypropylene, granulate [GLO]
**Processes**			
Al shaping	*pack_cont_al/pack_cont*	kg	Impact extrusion of aluminium, 1 stroke [RER]
Sheet rolling Fe	*pack_cont_fe/pack_cont*	kg	Sheet rolling, steel [RER]
Fe shaping	*pack_cont_fe/pack_cont*	kg	Deep drawing, steel, 10000 kN press, single stroke [RER]
**Transport**			
Freight train	0.044	tkm	Market group for transport, freight train [RER]
Freight lorry	0.171	tkm	Market for transport, lorry [RER]
Barge	0.022	tkm	Market for transport, inland waterways, barge [RER]
**Output**			
Battery jacket	1	kg	

5.5.2 Inventory battery management system

Different levels of detail for the battery management system (BMS) are used in existing LCA studies, whereby Ellingsen *et al.* (2014) provides the most detailed inventory (Peters & Weil, 2018). This inventory, which is based on primary data, is therefore used to model the BMS and includes the integrated battery interface system (IBIS), high voltage system, low voltage system and fasteners. The module electronic parts are excluded from the original inventory as they are already present in the module electronics inventory. Table S24 provides the inventory for the BMS. The LCI for the indivdiual components are not replicated here as they are identical to Ellingsen *et al.* (2014) but includes a general European transport service.

Table S24: Process inventory for battery management system

Description	Quantity	Unit	ecoinvent process
**Materials**			
IBIS	0.528	kg	
IBIS fasteners	3.0E-03	kg	
High voltage system	0.329	kg	
Low voltage system	0.142	kg	
**Transport**			
Freight train	0.044	tkm	Market group for transport, freight train [RER]
Freight lorry	0.171	tkm	Market for transport, lorry [RER]
Barge	0.022	tkm	Market for transport, inland waterways, barge [RER]
**Output**			
Battery management system	1	kg	

5.5.3 Inventory pack busbar

A copper busbar is added to packs with a single row to carry the current to the front of the pack (Nelson *et al.*, 2019). The busbar is modelled as a general Cu product based on Ellingsen *et al.* (2014) and identical to the inventory of the module interconnect as described in Table S25.

5.5.4 Inventory module interconnect

Modules are interconnected (negative to positive terminals) in BatPaC by a Cu connector with a default length of 6 mm (Nelson *et al.*, 2019). The Cu interconnects are modelled as a general Cu product based on Ellingsen *et al.* (2014).

Table S25: Process inventory for module interconnect

Description	Quantity/ function	Unit	ecoinvent process
**Materials**			
Cu	1	kg	Market for copper, cathode [GLO]
**Processes**			
Production	1	kg	Copper product manufacturing, averagae metal working [RER]
**Infrastructure**			
Facility	4.4E-10	unit	Metal working factory construction [RER]
**Transport**			
Freight train	0.044	tkm	Market group for transport, freight train [RER]
Freight lorry	0.171	tkm	Market for transport, lorry [RER]
Barge	0.022	tkm	Market for transport, inland waterways, barge [RER]
**Output**			
Module interconnect	1	kg	

5.5.5 Inventory coolant

The default coolant in BatPaC consists of a 50/50 ethylene-glycol/deionised water solution, which is a low cost coolant that is commonly used in battery systems (Nelson *et al.*, 2019). The ecoinvent market processes for ethylene glycol (GLO) and dionised water (RER) are used as proxies.

5.5.6 Inventory cooling tubes

The coolant tubes in BatPaC are made out of steel, and for the production of these the steel product manufacturing, average metal working is used.

Table S26: Process inventory for cooling tubes

Description	Quantity	Unit	ecoinvent process
**Materials**			
Steel	1	kg	Market for steel, low-alloyed [GLO]
**Processes**			
Production	1	kg	metal working, average for steel product manufacturing [RER]
**Infrastructure**			
Facility	4.4E-10	unit	Metal working factory construction [RER]
**Transport**			
Freight train	0.044	tkm	Market group for transport, freight train [RER]
Freight lorry	0.171	tkm	Market for transport, lorry [RER]
Barge	0.022	tkm	Market for transport, inland waterways, barge [RER]
**Output**			
Cooling tubes	1	kg	

5.5.7 Inventory cooling connectors

The coolant connector material is not disclosed in BatPaC. The pipe fitting inventory by Ellingsen *et al.* (2014) is instead used as a proxy, where a mixture of rubber and polyvinylchloride material are used. The inventory is identical and not replicated here.

5.5.8 Inventory cooling panels

The cooling panels in BatPaC version 5 are made out of a default 0.3 mm thick iron sheets. Iron sheet rolling is therefore used as a proxy to produce the panels. The inventory is thereby identical to the cooling tubes as highlighted in Table S26.

5.5.9 Inventory pack heater

When operating in cold temperatures, battery pack heaters are used to deliver full power at the vehicle startup if the temperature if less than 5°C (Nelson *et al.*, 2019). In BatPaC an electric heater is included to start-up the battery in cold temperatures. The weight of the heater is based on the power requirement (0.2 kg/kW) which differs by size of the battery and is therefore calculated in BatPaC. Pack heaters are typically not included in battery LCA and no information about the material of the pack heater is available in BatPaC. The pack heater by Crenna *et al.* (2021) based on a aluminium heating plate is therefore used as a proxy for the heating system.

Table S27: Process inventory for pack heater

Description	Quantity	Unit	ecoinvent process
**Materials**			
Al plate	1	kg	Market for aluminium, wrought alloy [GLO]
**Processes**			
Production	1	kg	Sheet rolling, aluminium [RER]
**Infrastructure**			
Facility	4.58E-10	unit	Metal factory construction [RER]
**Transport**			
Freight train	0.044	tkm	Market group for transport, freight train [RER]
Freight lorry	0.171	tkm	Market for transport, lorry [RER]
Barge	0.022	tkm	Market for transport, inland waterways, barge [RER]
**Output**			
Pack heater	1	kg	

5.5.10 Inventory pack terminals

In BatPaC, the pack terminals are made of 90% Cu as conductive material and 10% seal material. The seal material is modelled as reinforced plastic based on Crenna *et al.* (2021). The copper process requirement is based on Ellingsen *et al.* (2014) and a general metal working factory used as proxy for infrastructure requirement. Production is assumed to take place in Europe.

Table S28: Process inventory for pack terminals

Description	Quantity	Unit	ecoinvent process
**Materials**			
Cu conductor	0.90	kg	Market for copper, cathode [GLO]
Seal material	0.10	kg	Glass fibre reinforced plastic production,injection moulded [RER]
**Processes**			
Production	0.90	kg	Copper product manufacturing, average metal working [RER]
**Infrastructure**			
Facility	4.4E-10	unit	Metal working factory construction [RER]
**Transport**			
Freight train	0.044	tkm	Market group for transport, freight train [RER]
Freight lorry	0.171	tkm	Market for transport, lorry [RER]
Barge	0.022	tkm	Market for transport, inland waterways, barge [RER]
**Output**			
Pack terminals	1	kg	

6. Supporting information life cycle costing

The cradle-to-gate life cycle cost of batteries is based on the the production factor cost and material and energy costs. Following is an overview of the life cycle costing (LCC) model calculations for battery production and underlying data used for the case study. An example notebook can be found in the GitHub repository.

6.1 Factor requirement and costs

The factor requirements refer to the primary production factors including capital, land and building cost and labour. The sum of the factor requirements per production process is thereby equal to the value added

As the factory size has a large impact on battery cost (Philippot *et al.*, 2019; Nelson *et al.*, 2019), the factor requirements are adjusted to account for different factory output volumes. The estimated cost was calculated based on the exponential method as used in BatPaC, a common method to estimate the equipment cost of chemical plants when data is not available for a particular plant capacity (Peters *et al.*, 2003). With this method, the cost of a piece of equipment b as a result of production capacity scaling is based on the known cost of the same equipment a with X times the capacity raised to scaling factor q:

$$b = aX^q \tag{6.1}$$

The value for q depends on specific production equipment, where a value of 0.6 is used as rule of thumb for most equipment when data is not available (Peters *et al.*, 2003). This method was applied by Nelson *et al.* (2019) to calculate the cost of requirement for labour (hr/yr), production area (m^2) and capital ($million) as a result of varying production volumes. The factor requirements for production process q and a given production scale was obtained with Equation 6.2:

$$\mathbf{F} = \mathbf{F}_b \left(\frac{P}{P_b}\right)^q \tag{6.2}$$

$\mathbf{F}_b$ refers to the baseline physical factor requirement matrix, which is based on the baseline production plant in BatPaC version 5, producing 500,000 packs per year of 100 kWh each and operating 320 days per year. P_b refers to the annual baseline processing rate, and P to the modelled production process rate. q refers to the process-specific scaling factors which were all obtained from BatPaC. The battery production processes from BatPaC version 5 where matched to the foreground system processes based on the mapping highlighted in Table S1.

Table S1: BatPaC and foreground system process mapping

**BatPaC process**	**Foreground process**
warehouse	building and supporting systems
mixing cathode materials	mixing cathode materials
mixing anode materials	mixing anode materials
cathode coating and drying	cathode coating and drying
anode coating and drying	anode coating and drying
solvent recovery	cathode binder solvent waste recovery
cathode calendering	cathode calendering
anode calendering	anode calendering
cathode notching	cathode slitting
anode notching	anode slitting
vacuum drying cathode	final electrode drying
vacuum drying anode	final electrode drying
anode slitting	anode slitting
cathode slitting	cathode slitting
cell stacking	cell stacking
terminal welding	terminal welding
x-ray inspection	jelly roll enclosing
jelly roll enclosing	jelly roll enclosing
electrolyte filling and sealing	electrolyte filling and sealing
dry room management	electrolyte filling and sealing
cell formation	cell formation
module assembly	module and pack assembly
pack assembly	module and pack assembly
building support systems	building and supporting systems
rejected cell and scrap recycling	building and supporting systems
control lab	building and supporting systems

To obtain the value added (v) per production process (bp), the mixed-unit factor requirement matrix ($\mathbf{F}$) is multiplied with the factor specific (k) cost vector, π (Duchin & Levine, 2011):

$$\mathbf{v_{bp}} = \pi_k \mathbf{F}_{k,bp} \tag{6.3}$$

Table S2 provides an overview of the factor prices of land, labour and capital for the considered European countries. European average in Table S2 refers to the average of the seven battery producing countries as used in the case study (Germany, Sweden, Norway, UK, Poland, Hungary and France). European labour cost are based on the average industry, construction and services labour costs obtained from Eurostat (2021*c*), which where previously used by both Philippot *et al.* (2019) and Duffner *et al.* (2020*a*) to estimate labour costs for a European bat-

tery cell production facility. Cost of land refers to building costs. Country specific building cost estimates are based on the two step approach by Duffner *et al.* (2020*a*). Here the detailed cost estimate for an industrial high tech factory/laboratory in the UK as reported by Townsend (2020) is used as a baseline, which are then multiplied by the percentage values of construction costs for the different countries as reported by the ECC (2021). It should be noted that, unlike BatPaC, energy costs are not included in the land and building costs but instead calculated based on the actual energy consumption and energy prices as highlighted below.

Table S2: Production factor cost in US Dollar. Labour cost obtained from Eurostat (2021*c*); land refers to building cost based which is calculated similar to Duffner *et al.* (2020*a*) based on data obtained from Townsend (2020) and ECC (2021).

**Country**	**land ($/m^2)**	**labour ($/hr)**	**capital ($/$)**
France	2380	43	1.00
Germany	2214	42	1.00
Hungary	1220	12	1.00
Norway	3683	59	1.00
Poland	1503	13	1.00
Sweden	3075	43	1.00
Great Britain	2291	34	1.00
European average	2338	35	1.00

6.2 Material and energy prices

Following is an overview of the material and energy prices used in the LCC model. The material prices are based on mass prices (i.e. $ per kg material) and an additional unit price for several materials (i.e. $ per unit)

6.3 Material prices - Mass

Material mass prices are presented in Table S3. Most material prices are obtained from the Shanghai Metals Market (SMM) and the BatPaC cost model. However, several assumptions and additional calculates were required.

- Six different battery separator types with a varying thickness and coating layer are available from the SMM. The price data for separator was converted from m^2 to kg based on Equation 5.1 in Section 5.3.4.
- Three types of electrolyte are available from the SMMM, including NMC, LMO and LFP chemistries. It is assumed that NCA chemistries require NMC electrolytes and LMO electrolyte is only for pure LMO (i.e. LMO-NMC blends use NMC electrolyte).
- Price data for Li-NMC333, Li-NCA and Li-NMC532/LMO were not available and instead calculated. A description of this can be found in Section 6.6.

- Prices for all different current collectors thicknesses included in the model are not available. While BatPaC uses a single price irrespective of the thickness, a small price difference between current collectors can be observed on the SMM. For LIB Al foils, the SMM reports a price of 2.9, 2.8 and 2.7 \$ kg^{-1} for 12, 13 and 15 µm, respectively while LIB Cu foil has a price range of 7.28 and 5.15 \$ kg^{-1} for 6 and 8 µm (foil prices refers to the processing fees of foils and excludes the cost of raw Cu and Al). To avoid the use of an average value for current collectors, the following assumption was made. For Al foil, 2.9 \$ kg^{-1} was used for 10, 11 and 12 µm, 2.7 for \$ kg^{-1} 13 and 14 µm and 2.4 for 15-18 µm. For Cu foil, it was assumed that 6 and 7 µm foils are sold with a treatment charges of 7.28 \$ kg^{-1} while 8 - 14 µm are fixed to 5.72 \$ kg^{-1}. An additional cost of 9.00 kg^{-1} is included to account for the current Cu price and 2.7 \$/kg to account for the Al price (see Table S7).

Table S3: Battery material mass prices. SMM price as of the 13th May 2022

Material	Price	Unit	Source
natural graphite	9.11	\$/kg	SMM (2022)
synthetic graphite	12.15	\$/kg	SMM (2022)
SiO	60.00	\$/kg	Greenwood *et al.* (2021)
LMO[a]	16.64	\$/kg	SMM (2022)
LFP	21.43	\$/kg	SMM (2022)
NCA	39.64	\$/kg	See Section 6.6
NMC333	42.31	\$/kg	See Section 6.6
NMC532	51.40	\$/kg	SMM (2022)
NMC622	54.49	\$/kg	SMM (2022)
NMC811	60.09	\$/kg	SMM (2022)
NMC532/50%/LMO	34.02	\$/kg	Calculated 50% NMC532-50%LMO
cathode binder (PVDF)	15.00	\$/kg	BatPaC version 5
anode binder (CMC)	5.00	\$/kg	Greenwood *et al.* (2021)
anode binder additive (SBR)	5.00	\$/kg	Greenwood *et al.* (2021)
carbon black	3.60	\$/kg	Alibaba
binder solvent (NMP)	2.00	\$/kg	Alibaba
binder solvent (deionised water)	0.00	\$/kg	Assumed free
Al foil (10-12 um)[a]	5.65	\$/kg	SMM (2022)
Al foil (13 &14 um)[b]	5.50	\$/kg	SMM (2022)
Al foil (15-18 um)[c]	5.35	\$/kg	SMM (2022)
Cu foil (6 um)[d]	15.77	\$/kg	SMM (2022)
Cu foil (8-14 um)[e]	14.15	\$/kg	SMM (2022)
electrolyte (NMC/NCA)	15.54	\$/kg	SMM (2022)
electrolyte (LFP)	13.03	\$/kg	SMM (2022)
electrolyte (LMO)	12.89	\$/kg	SMM (2022)
separator (5um)	195.56	\$/kg	SMM (2022)
separator (7um)	98.41	\$/kg	SMM (2022)
separator (9um)	51.85	\$/kg	SMM (2022)
coated separator (5um+2um)	83.31	\$/kg	SMM (2022)
coated separator (7um+2um)	51.81	\$/kg	SMM (2022)
coated separator (9um+3um)	32.88	\$/kg	SMM (2022)
cell terminal cathode	8.64	\$/kg	BatPaC version 5
cell terminal anode	2.41	\$/kg	BatPaC version 5

cell container	3.00	\$/kg	BatPaC version 5
module polymer panels	2.30	\$/kg	BatPaC version 5
module row rack	1.33	\$/kg	BatPaC version 5
module container	2.40	\$/kg	BatPaC version 5
module terminal	8.64	\$/kg	BatPaC version 5
module thermal conductor	2.41	\$/kg	BatPaC version 5
cooling panels	2.80	\$/kg	BatPaC version 5
cooling mains Fe	8.00	\$/kg	BatPaC version 5
cooling connectors	8.00	\$/kg	BatPaC version 5
pack terminals	8.88	\$/kg	BatPaC version 5
cell group interconnect	8.72	\$/kg	BatPaC version 5
battery jacket steel	1.40	\$/kg	BatPaC version 5
battery jacket aluminium	2.81	\$/kg	BatPaC version 5
battery jacket insulation[f]	9.38	\$/kg	BatPaC version 5
module interconnects	5.00	\$/kg	BatPaC version 5
busbar	8.68	\$/kg	BatPaC version 5

[a] Based on 12 um Al LIB foil price from SMM
[b] Based on 13 um Al LIB foil price from SMM
[c] Based on 15 um Al LIB foil price from SMM
[d] Based on a treatment charge for 6 um Cu LIB foil price from SMM and current Cu metal price
[e] Based on a treatment charge for 8 um Cu LIB foil price from SMM and current Cu metal price
[f] Based on a density of 0.32 g/cm^3 and a default thickness of 10 mm

6.4 Material prices - unit

Unit material prices are presented in Table S4. Unit prices refer to the additional unit price based on different pack specific parameters (unit column). All prices are based on BatPaC. The battery management system unit, pack thermal system and additional cost for the AC system are calculated with BatPaC for each battery model pack.

Table S4: Battery material prices per unit based on BatPaC version 5.

Good	Parameter	Value	Unit
cell container	cells_per_pack	0.20	$/unit
cell terminal cathode	cells_per_pack	0.08	$/unit
cell terminal anode	cells_per_pack	0.08	$/unit
module thermal conductor	cells_per_pack	0.15	$/unit
module electronics	module_capacity_ah	0.03	$/unit
	cell_series_in_module[a]	2.00	$/unit
cell group interconnect	total_cell_interconnects	0.04	$/unit
module polymer panels[b]	modules_per_pack	0.40	$/unit
module terminal[b]	modules_per_pack	0.36	$/unit
module container	modules_per_pack	0.50	$/unit
gas release	modules_per_pack	0.50	$/unit
module row rack	total_packs_vehicle	1.00	$/unit
module elastomer pads	total_elastomer_pads	0.20	$/unit
module interconnects	module_interconnect_total	0.40	$/unit
busbar	total_packs_vehicle	0.60	$/unit
cooling panels[b]	rows_of_modules	1.00	$/unit
cooling mains Fe	total_packs_vehicle	1.00	$/unit
pack terminals[b]	total_packs_vehicle	0.75	$/unit
battery jacket Fe	total_packs_vehicle	3.00	$/unit
battery jacket Al	total_packs_vehicle	3.00	$/unit
battery management system	battery_management_system_cost	1.00	$/unit
pack heater	cost_pack_heating_thermal	1.00	$/unit
	addition_cost_ac_system[c]	1.00	$/unit

[a] Based on cells per module divided by cells in parallel.

[b] Multiplied value by 2 based on "manufacturing costs" sheet in BatPaC V5.

[c] Allocated to pack heater.

6.5 Energy prices

Energy prices, including gas and electricity, for the seven considered battery producing locations are presented in Table S5.

Table S5: Electricity and gas prices for the top 7 battery producing countries in Europe. Electricity prices based on Eurostat (2021*a*) and gas based on (Eurostat, 2021*b*)

**Country**	**Electricity ($ kWh^{-1})**	**Natural gas ($ MJ^{-1})**
FR	0.113	0.012
DE	0.181	0.010
HU	0.110	0.009
NO	0.091	0.010
PL	0.104	0.011
SE	0.084	0.012
GB	0.176	0.009
European average	0.122	0.010

6.6 Calculation cathode active material

Prices for NMC532-LMO, NMC333 and NCA are not available from the Shanghai Metals Market (SMM). NMC532-LMO, consisting of 50% LMO and NMC532, is calculated based on the price of LMO and NMC532, similar to LMO-NMC$_{xyz}$ blend calculation in BatPaC (Nelson *et al.*, 2019). The price for NMC333 and NCA are calculated based on the difference between intermediate metal costs and available CAM sales prices. Greenwood *et al.* (2021) refers to this difference as the product costs and profit margins (PCPM) and calculates this based on available CAM prices and intermediate material prices obtained from the Shanghai Metals Market (SMM).

To obtain the price for NMC333 and NCA, the metal cost are calculated based on the current metal prices (see also Section 6.6.1) and the PCPM of a comparable CAM. The PCPM for NMC333 is based on NMC532, as both are relatively mature technologies and have a comparable PCPM (Greenwood *et al.*, 2021). NCA has a higher PCMP compared to low Ni NMC due to its slightly lower yield and additional raw materials required (Nelson *et al.*, 2012). The PCMP of NCA is therefore based on NMC811, similar to Greenwood *et al.* (2021).

The PCPM values are also used to account for a price sensitive analysis by varying metal prices. The PCPM estimations for all cathode active materials considered are presented in Table S6. The differentiation in PCPM estimates between LFP, NMC532, NMC622 and NMC811 can be explained due to the differences in technology maturity (Greenwood *et al.*, 2021). Based on the current metal and CAM prices, the PCPM of LMO is negative. While the process cost of LMO is lower than other CAM due to its ease of manufacturing (Nelson *et al.*, 2012), the negative CAM price can be explained due to recent price surge of LiCO to a record high (Reuters, 2021). To account for a positive PCPM, the values of the PCPM calculation by Susarla & Ahmed (2020) are instead used in the sensitivity analysis. The authors calculate the PCPM (overheads, labour, utility costs, depreciation and profit) for LMO based on a process model for a solid-state synthesis process route utilising MnO_2.

Table S6: Cathode active material process cost and profit margin calculation (PCPM) based on current metal cost (1-2022) and cathode active material (CAM) price as reported by the SMM (2022). The PCPM price for NMC333 is based on NMC532 estimate and NCA is based on NMC622 due to lack of CAM price data.

**Cathode**	**Unit**	**Metal cost**	**CAM price**	**Calculated PCPM**	**Used PCPM**
LFP	\$/kg	16.23	21.43	5.20	5.30
LMO	\$/kg	17.87	16.64	-1.22	3.11[a]
NMC333	\$/kg	47.62	54.34[b]	6.72	6.72
NMC532	\$/kg	44.69	51.40	6.72	6.72
NMC622	\$/kg	46.13	54.59	8.36	7.19
NMC811	\$/kg	45.04	60.09	15.05	15.05
NCA	\$/kg	47.65	62.70[c]	15.05	15.05
NMC532/LMO	\$/kg	31.27	34.02	2.54	4.92

[a] Based on reported value by Ahmed *et al.* (2021).

[b] Calculated based on the current metal price and the PCPM of NMC532.

[c] Calculated based on the current metal price and the PCPM of NMC811.

6.6.1 Current mineral prices

Current mineral prices are based on a variety of sources (see Table S7). For Ni, Co, Mn and Li, the current (13-05-2022) prices of intermediate cathode active materials (NiSO, CoSO, MnSO and LiCO, respectively) available from the SMM are used, and adjusted to account for the elemental prices. Al and Cu are based on 3-month contract prices obtained from the London Metal Exchange (). P and Fe are based on the phosphate rock (North Africa) and iron ore prices for December 2021 as reported by the World Bank. For Si, the current price for Si Metal Sichuan (Si 99%) as reported by the SMM is used, similar to the monthly price monitor from the German Federal Institute for Geosciences and Natural Resources (DERA, 2021*a*). For natural graphite, the average price for 2020 of small flake graphite, the type used to make battery anode materials (USGS, 2017), as reported by Statista (2022) is used.

Table S7: Current mineral prices in dollar per kg of pure element

Mineral	Price ($ kg^{-1})	Type	Source
Ni	24.39	NiSO	SMM (2022)
Al	2.90	LME Al 3 months contract	LME (2022)
P	1.26	Phosphate rock, North Africa[a]	World Bank (2022)
Fe	0.19	Iron ore[b]	World Bank (2022)
Cu	9.00	LME Cu 3 months contract	LME (2022)
C	0.49	Small flake	Statista (2022)
Li	225.48	LiCO	SMM (2022)
Si	3.22	Metal Yannan, Sichuan	DERA (2021*b*)
Mn	4.82	MnSO	SMM (2022)
Co	76.32	CoSO	SMM (2022)

[a] Based on phosphate rock price, North Africa, with 32% P_2O_5 content based on Moroccan phosphate rock (USGS, 2021*a*)

[b] Based on iron ore price with 62% Fe content (World Bank, 2022)

6.6.2 Historic mineral prices.

Historic mineral prices (2000-2021) are used to obtain a minimum and maximum price as input for the price sensitivity analysis. Real dollar values (2020) and sources used for all minerals are provided in Table S8.

Table S8: Historic low and high real 2020 prices per kg of pure element

	low	high	Type	Source
Ni	7.05	65.13	Cathode, LME	World Bank (2022)
Al	1.46	3.69	Ingot, LME	World Bank (2022)
P	2.81	27.81	Phosphate rock[a]	World Bank (2022)
Fe	0.11	0.63	Iron ore[b]	World Bank (2022)
Cu	2.01	11.35	>99%, LME	World Bank (2022)
C	0.57	2.05	Fine, -100 mesh[c]	USGS (2021*b*)
Li	22.48	217.92[d]	LiCO	USGS (2021*b*); DERA (2021*a*)
Si	1.17	4.30	Si metal	USGS (2021*b*); DERA (2021*a*)
Mn	3.31	14.60	Mn ore	USGS (2021*b*)
Co	8.90	114.22	US cathodes	IMF (2022)

[a] Based on phosphate rock price, North Africa, with 32% P_2O_5 content based on Moroccan phosphate rock (USGS, 2021*a*).

[b] Based on iron ore price with 62% Fe content (World Bank, 2022).

[c] The type used to make battery anode materials (USGS, 2017). Average of 95.5% carbon.

[d] Based on most recent price of LiCO as reported on the SMM.

For Cu, Ni, Al, Fe and P, prices are based on monthly prices obtained from the World Bank (2022) and the (IMF, 2022) for Co and adjusted for inflation based on the annual Consumer Price Index (OECD, 2022). A historic price overview is illustrated in Figure S1. Historic prices from 2000-2020 for Mn, Li, C and Si are based on annual nominal prices obtained from the annual USGS Mineral Commodity Summaries (USGS, 2021*a*). The following product types are used: Si metal, Mn ore, crystalline fine (-100 mesh, 94-97% and 90% prior to 2013) graphite flakes and LiCO. For Li prices from 2010-2020 are based on battery grade LiCO as reported by the USGS, prior to this data prices are based on average LiCO. For C, different flake size products are available (large, medium, fine) with varying prices, whereby the fine flake graphite is used to make battery anode materials (USGS, 2017). The average value of the reported range price of fine graphite is therefore used. Furthermore, average prices for Li and Si in 2021 (December 2020-November 2021) are obtained from DERA (2021*a*) and the current price from SMM (2022) to include the latest price peaks for both metals. Matching monthly price data for other minerals from this source were not available.

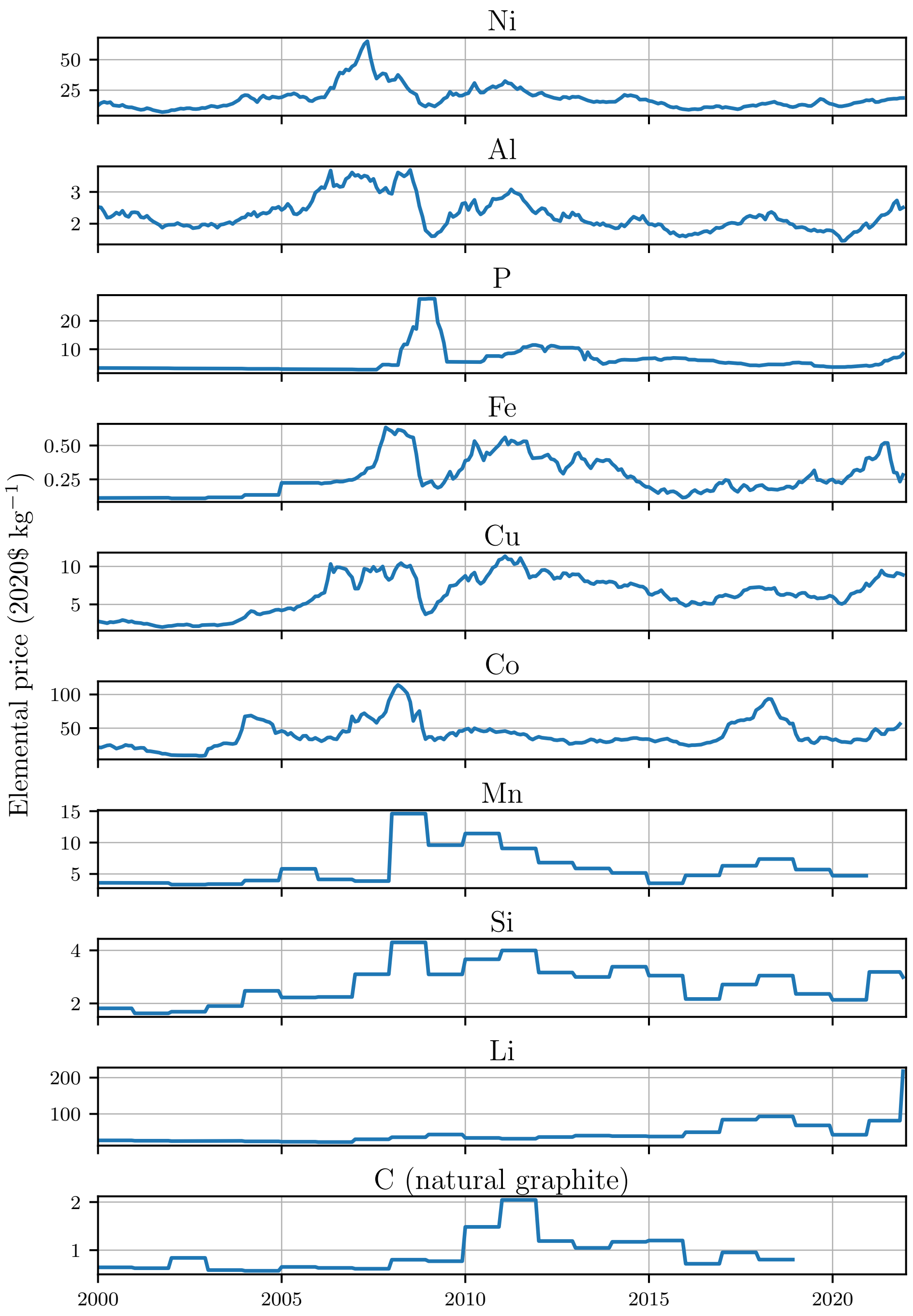

Figure S1: Monthly real price (2020) per kg of pure element between 2000 and 2020. Sources can be found in Table S8

7. Sensitivity analysis

7.1 Sensitivity analysis carbon footprint

A one-at-the-time sensitivity analysis was conducted to assess the impact of the carbon footprint by changing the parameter values for battery manufacturing energy consumption, battery production location and material production emissions (Table S1). For the battery manufacturing energy consumption, the default consumption values are comparatively low (ranging between 12.4 to 27 Wh/Wh). To obtain a higher value comparable to other studies, the energy consumption parameters (exception of electricity for cell formation) is multiplied by a factor of 5 (see also Figure S3). Multiplication by a factor of 5 results in a energy consumption ranging between 53.4 to 107.6 Wh of energy per Wh of battery pack, comparable with the higher energy consumption values found in the literature (e.g. 106 Wh Wh^{-1} reported by Yuan *et al.* (2017) and 96 Wh Wh^{-1} reported by Chordia *et al.* (2021)). Different material production are obtained from a variety of literature sources and presented in Table S1.

The results of the carbon footprint parameter sensitivity analysis are presented in Figure S1.

Table S1: Parameters and values considered for the sensitivity analysis for the battery carbon footprint. Acronyms: Poland (PL); Sweden (SE).

**Parameter**	**Unit**	**Low**	**Used**	**High**	**Sources**
Location	Country	SE	Average	PL	
Energy	factor	-	1	5	See Figure S4
Syn. graphite	kg CO_2-eq.	1.82	5.29	20.60	Low: Manjong *et al.* (2021)<br>High: Surovtseva *et al.* (2022)
Nat. graphite	kg CO_2-eq.	2.34	11.43	-	Manjong *et al.* (2021)
LiCO	kg CO_2-eq.	-	2.09	20.4	Kelly *et al.* (2021) - ore
LiHO	kg CO_2-eq.	-	5.71	15.7	Kelly *et al.* (2021) - ore
Al alloy	kg CO_2-eq.	5.55	13.34	36.56	Manjong *et al.* (2021)
Cu cathode	kg CO_2-eq.	3.16	6.50	13.39	Manjong *et al.* (2021)
Mn sulfate[a]	kg CO_2-eq.	-	0.78	3.51	Manjong *et al.* (2021)
Mn dioxide[b]	kg CO_2-eq.	-	2.62	4.54	Manjong *et al.* (2021)
Ni sulfate[c]	kg CO_2-eq.	1.75	4.46	12.91	Low: Terrafame (2020)<br>High: Manjong *et al.* (2021)
Co sulfate[d]	kg CO_2-eq.	1.58	4.75	-	Pell & Tijsseling (2020)
Co hydroxide	kg CO_2-eq.	2.56	29.19	-	Dai *et al.* (2018*b*)
Silicon	kg CO_2-eq.	-	10.55	48.5	ecoinvent - solar Si

[a] Based on the ecoinvent Mn sulfate inventory and the GWP values for Mn concentrate values from Manjong *et al.* (2021).

[b] Used for LMO production. Based on the ecoinvent Mn dioxide inventory and the high GWP value for Mn concentrate values from Manjong *et al.* (2021).

[c] High values based on the ecoinvent Ni sulfate inventory and the high GWP values for Ni metal values from Manjong *et al.* (2021).

[d] Refers to the gate-to-gate emissions (Co hydroxide to Co sulfate).

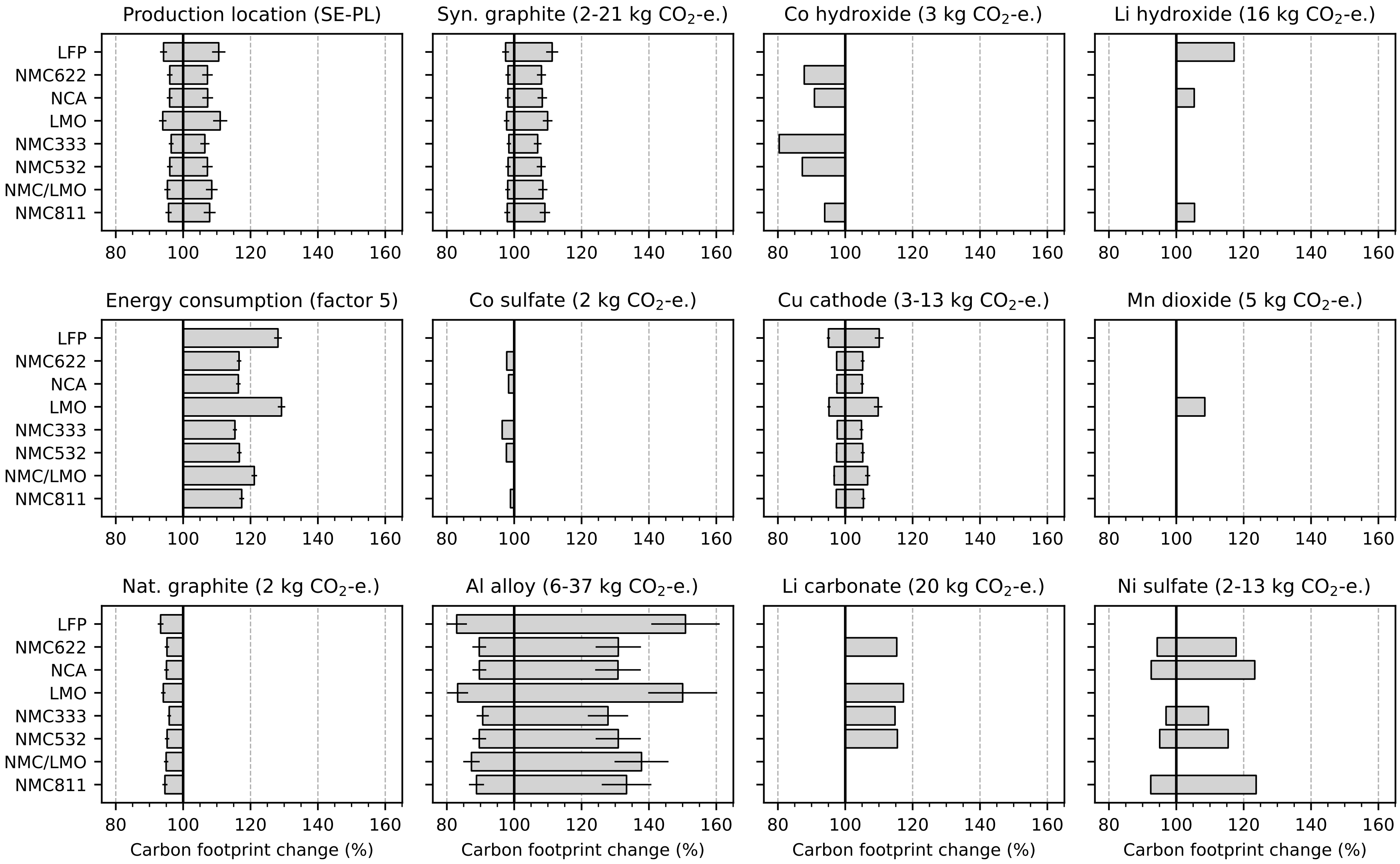

Figure S1: Sensitivity analysis for carbon footprint of all battery designs. The parameter values used can be found in Table S1

7.2 Sensitivity analysis cost

A sensitivity analysis was conducted to investigate the influence of different parameters on the total battery cost. Based on the factors explaining the different modelled cost results and those found in the literature, the following parameters are included: battery production locations based on key European battery manufacturing countries, annual pack manufacturing capacity and different mineral prices based the historical mineral prices between 2000 and 2022 (see Table S2).

Table S2: Parameters and values considered for the sensitivity analysis for the pack price. Mineral prices are based on historic prices, sources used can be found in Table S8 in Appendix B.

Parameter	Unit	Lower bound	Used in study	Upper bound
Location	Country	Norway	Average	Hungary
Capacity	packs/year	100,000	500,000	700,000
Nickel price	$/element	7.05	24.39	65.13
Aluminium price	$/element	1.146	2.81	3.69
Phosphorus price	$/element	2.81	2.81	27.81
Iron price	$/element	0.11	0.19	0.63
Lithium price	$/element	22.48	361	361
Manganese price	$/element	3.31	4.82	14.6
Cobalt price	$/element	8.9	76.32	114.22

Due the strong correlation between mineral prices and cathode chemistry, the results of the sensitivity analysis are presented in Figure S2 by cathode chemistry.

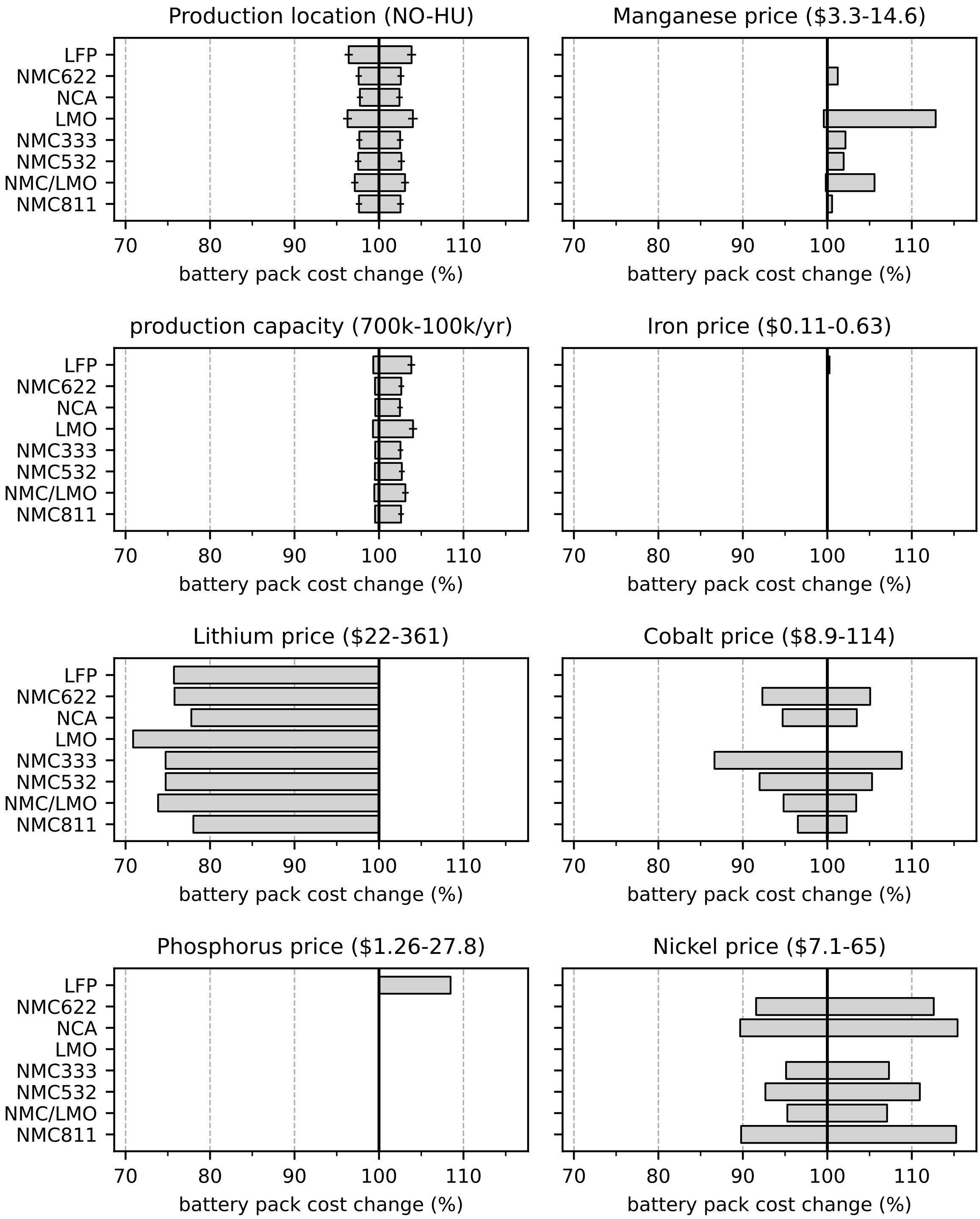

Figure S2: Sensitivity analysis of the battery cost for the most sensitive parameters. Baseline refer to the average cost of the chemistry for all designs. Mineral prices are based on pure elemental values.

8. Supporting results figures

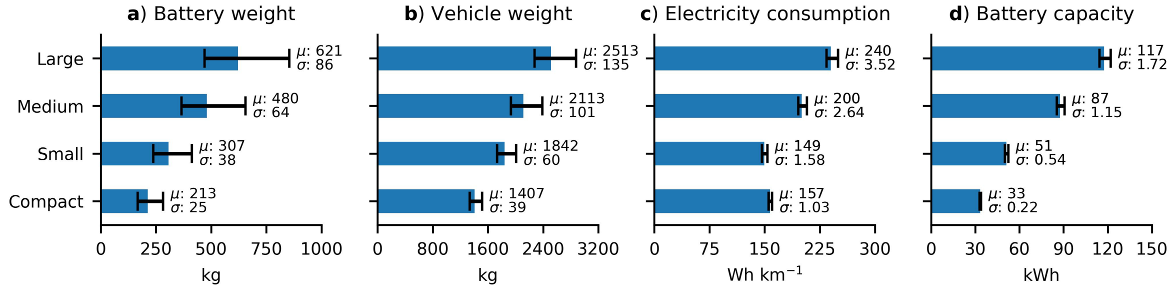

Figure S1: Overview of the modelled battery weight (a), vehicle weight (b), vehicle electricity consumption (c) and required battery capacity (d) for four different vehicle segments.

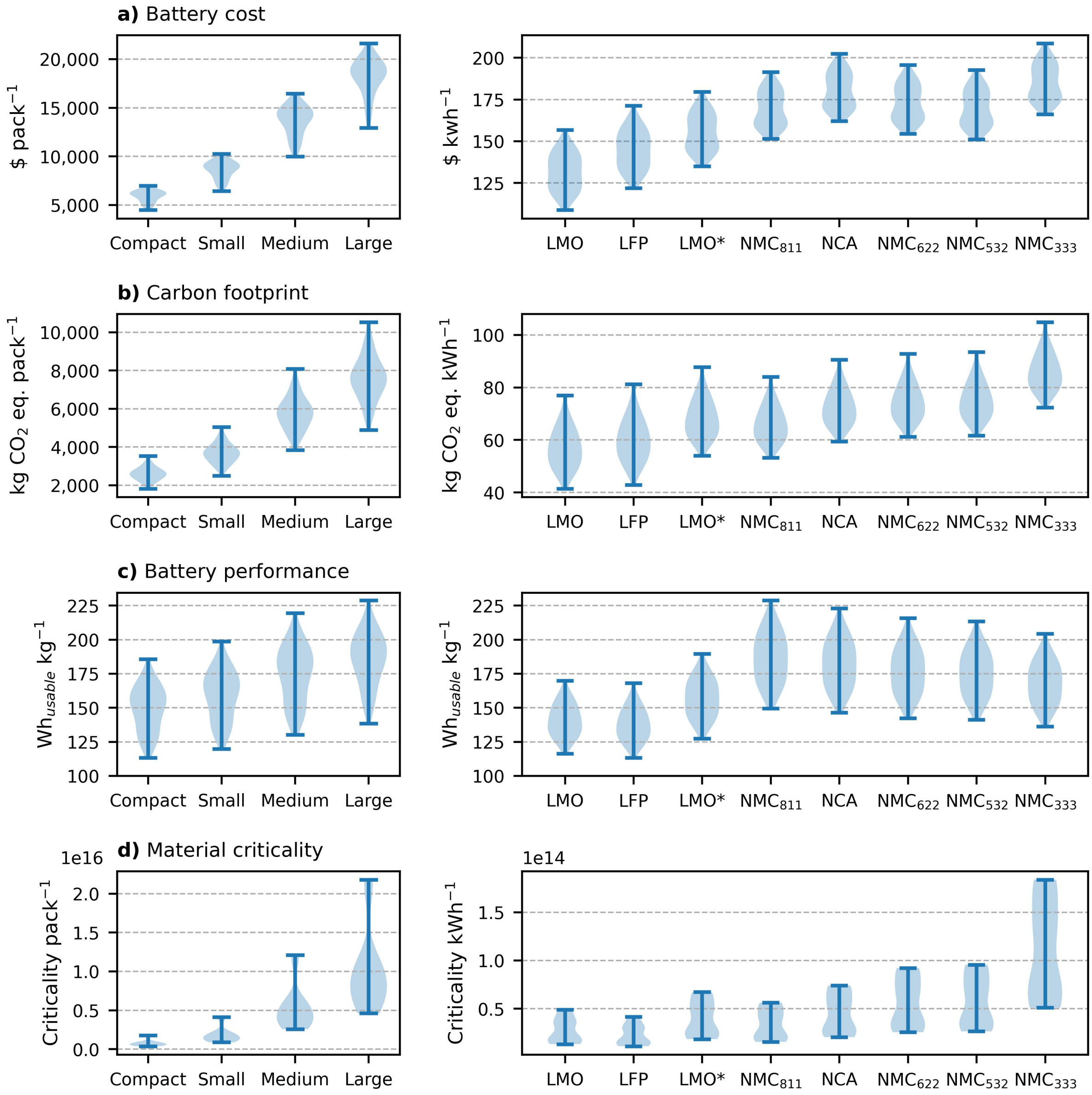

Figure S2: Overview of battery cost, carbon footprint, performance and criticality per pack and segment (left figures) and per kWh and cathode chemistry (right figures) for all 20,340 battery designs. Regional cost and emissions production parameters are based on average values for Germany, Sweden, Norway, UK, Poland, Hungary and France. Battery factory has a size of 500,000 packs per year and cathode material prices as of 13-04-2022 obtained from the Shanghai Metals Market (SMM, 2021). LMO* refers to LMO-NMC532 (50:50).

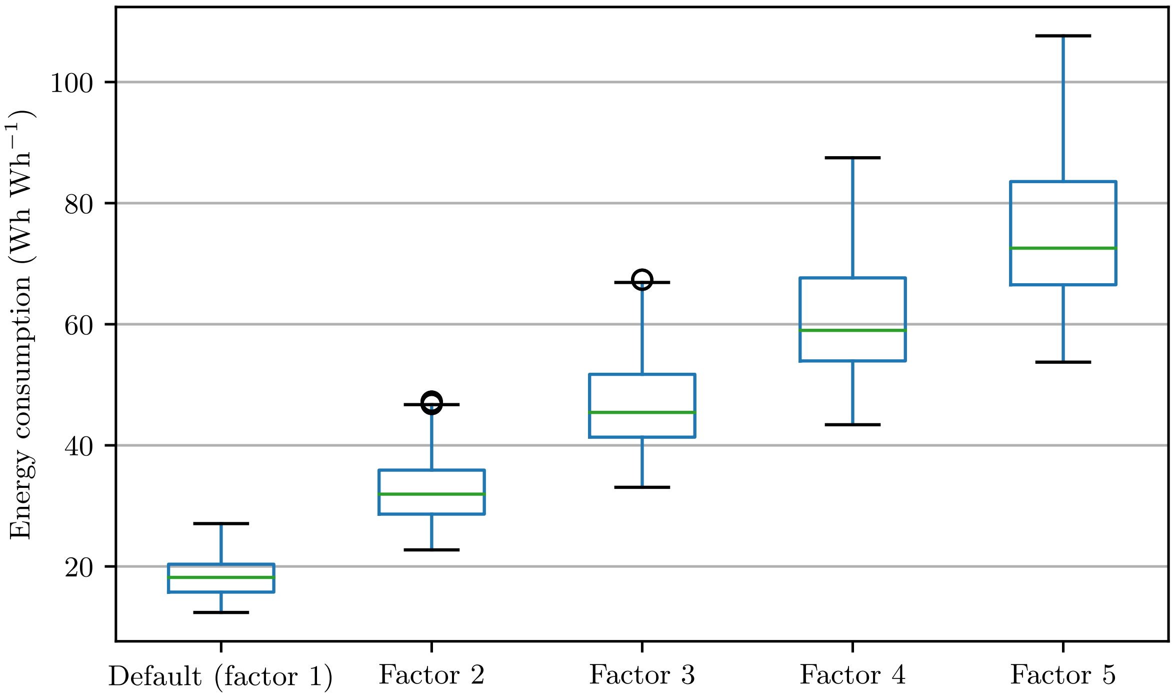

Figure S3: Battery manufacturing energy consumption scenarios. Scenarios refer to the multiplication factor of the energy consumption parameters (excluding electricity for formation).

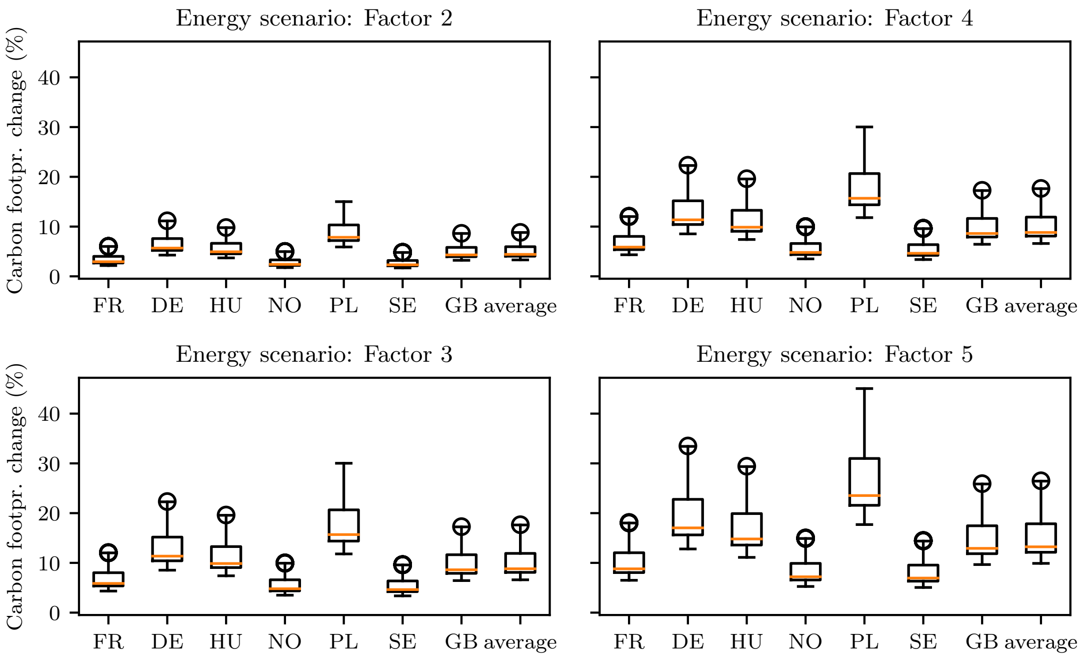

Figure S4: Percentage battery carbon footprint increase by changing battery production country and energy consumption. Energy scenarios refer to the multiplication factor of the energy consumption parameters (excluding electricity for formation).

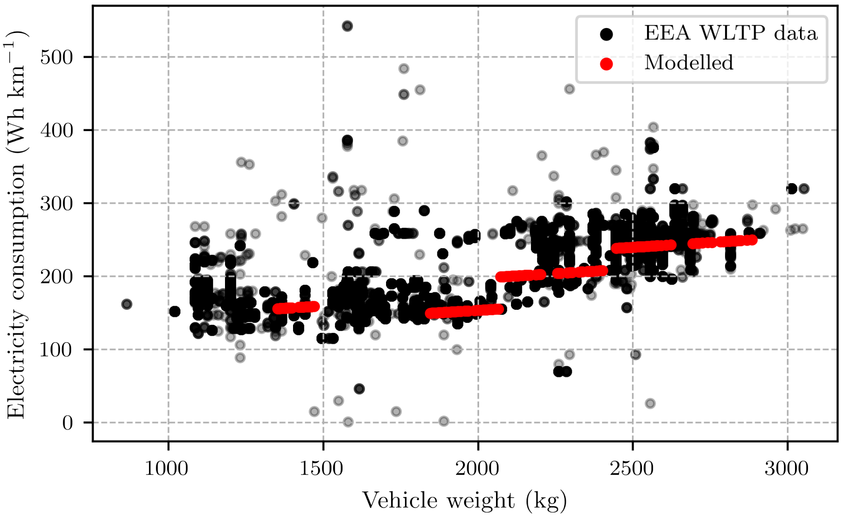

Figure S5: Comparison of the modelled vehicle electricity consumption with real values. Real values are obtained from the European Environmental Agency CO_2 database for all electric vehicles registered between 2012 and 2020 (EEA, 2021).

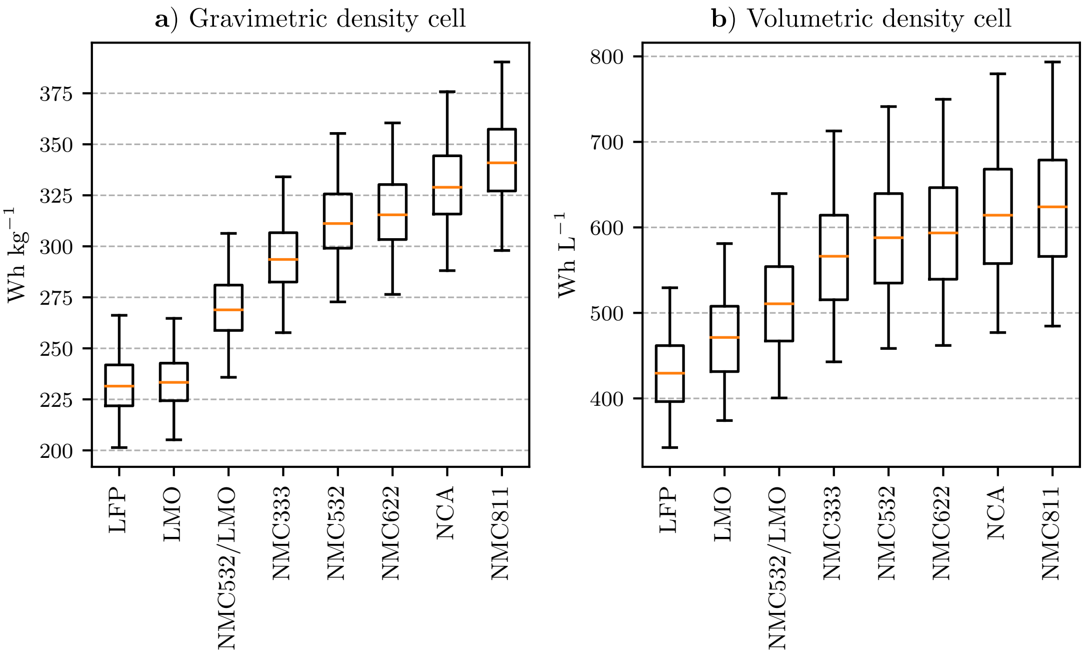

Figure S6: Comparison of gravimetric and volumetric cell energy density for all modelled pack designs

Bibliography

A2Mac1 2021 Automotive benchmarking. https://portal.a2mac1.com/, [Accessed January 1st, 2022].

Ahmed, S., Nelson, P., Kubal, J., Liu, Z., Knehr, K. & Dees, D. 2021 Estimated cost of ev batteries 2018-2021 - 2021 updates. https://www.anl.gov/cse/batpac-model-software, [Accessed January 1st, 2022].

Ahmed, S., Nelson, P. A., Gallagher, K. G., Susarla, N. & Dees, D. W. 2017 Cost and energy demand of producing nickel manganese cobalt cathode material for lithium ion batteries. *Journal of Power Sources* **342**, 733–740.

Aichberger, C. & Jungmeier, G. 2020 Environmental life cycle impacts of automotive batteries based on a literature review. *Energies* **13** (23), 1–27.

Armand, M., Axmann, P., Bresser, D., Copley, M., Edström, K., Ekberg, C., Guyomard, D., Lestriez, B., Novák, P., Petranikova, M., Porcher, W., Trabesinger, S., Wohlfahrt-Mehrens, M. & Zhang, H. 2020*a* Lithium-ion batteries – current state of the art and anticipated developments. *Journal of Power Sources* **479**, 228708.

Armand, M., Axmann, P., Bresser, D., Copley, M., Edström, K., Ekberg, C., Guyomard, D., Lestriez, B., Novák, P., Petranikova, M., Porcher, W., Trabesinger, S., Wohlfahrt-Mehrens, M. & Zhang, H. 2020*b* Lithium-ion batteries – current state of the art and anticipated developments. *Journal of Power Sources* **479**, 228708.

Asenbauer, J., Eisenmann, T., Kuenzel, M., Kazzazi, A., Chen, Z. & Bresser, D. 2020 The success story of graphite as a lithium-ion anode material – fundamentals, remaining challenges, and recent developments including silicon (oxide) composites. *Sustainable Energy Fuels* **4** (11), 5387–5416.

Baars, J., Domenech, T., Bleischwitz, R., Melin, H. E. & Heidrich, O. 2021 Circular economy strategies for electric vehicle batteries reduce reliance on raw materials. *Nature Sustainability* **4** (1), 71–79.

Ball, S., Clark, J. & Cookson, J. 2020 Battery materials technology trends and market drivers for automotive applications. *Johnson Matthey Technology Review* **64** (3), 287–297.

Benavides, P. T., Dai, Q., Sullivan, J. L., Kelly, J. C. & Dunn, J. B. 2015 Material and energy flows associated with select metals in greet 2. molybdenum, platinum, zinc, nickel, silicon. https://greet.es.anl.gov/publication-mo-pt-zn-ni-si, [Accessed February 3rd, 2022].

Biswal, A., Chandra Tripathy, B., Sanjay, K., Subbaiah, T. & Minakshi, M. 2015 Electrolytic manganese dioxide (emd): a perspective on worldwide production, reserves and its role in electrochemistry. *RSC Advances* **5** (72), 58255–58283.

Boer, L., Pescatori, A. & Stuermer, M. 2021 Energy transition metals. https://www.imf.org/en/Publications/WP/Issues/2021/10/12/Energy-Transition-Metals-465899, [Accessed January 1st, 2022].

Bryntesen, S. N., Strømman, A. H., Tolstorebrov, I., Shearing, P. R., Lamb, J. J. & Stokke Burheim, O. 2021 Opportunities for the state-of-the-art production of lib electrodes—a review. *Energies* **14** (5).

Chen, J. & Whittingham, M. S. 2006 Hydrothermal synthesis of lithium iron phosphate. *Electrochemistry Communications* **8** (5), 855–858.

Chen, T., Wu, J., Zhang, Q. & Su, X. 2017 Recent advancement of siox based anodes for lithium-ion batteries. *Journal of Power Sources* **363**, 126–144.

Chordia, M., Nordelöf, A. & Ellingsen, L. A.-W. 2021 Environmental life cycle implications of upscaling lithium-ion battery production. *The International Journal of Life Cycle Assessment* **26** (10), 2024–2039.

Chu, H.-C. & Tuan, H.-Y. 2017 High-performance lithium-ion batteries with 1.5 m thin copper nanowire foil as a current collector. *Journal of Power Sources* **346**, 40–48.

CIC energiGUNE 2021 World map of gigafactories. https://cicenergigune.com/en/blog/world-map-gigafactories, [Accessed January 1st, 2022].

Cox, B., Bauer, C., Mendoza Beltran, A., van Vuuren, D. P. & Mutel, C. L. 2020 Life cycle environmental and cost comparison of current and future passenger cars under different energy scenarios. *Applied Energy* **269**, 115021.

Crenna, E., Gauch, M., Widmer, R., Wäger, P. & Hischier, R. 2021 Towards more flexibility and transparency in life cycle inventories for lithium-ion batteries. *Resources, Conservation and Recycling* **170**, 105619.

Dai, Q., Dunn, J., Kelly, J. C. & Elgowainy, A. 2017*a* Upate of life cycle analysis of lithium-ion batteries in the greet model. Report. Argonne National Laboratory.

Dai, Q., Dunn, J. B., Kelly, J. C. & Elgowainy, A. 2017*b* Update of life cycle analysis of lithium-ion batteries in the greet model. https://greet.es.anl.gov/publication-Li$_{b}attery_{u}pdate_{2}$017, [$AccessedJanuary1st$, 2022].

Dai, Q., Kelly, J. C., Dunn, J. B. & Benavides, P. T. 2018*a* Update of bill-of-materials and cathode materials production for lithium-ion batteries in the greet model. https://greet.es.anl.gov/publication-update_bom_cm, [Accessed December 19, 2021].

Dai, Q., Kelly, J. C. & Elgowainy, A. 2018*b* Cobalt life cycle analysis update for the greet mode. https://greet.es.anl.gov/publication-update_cobalt, [Accessed December 19, 2021].

Dai, Q., Kelly, J. C., Gaines, L. & Wang, M. 2019*a* Life cycle analysis of lithium-ion batteries for automotive applications. *Batteries* **5** (2), 48.

Dai, Q., Spangenberger, J., Ahmed, S., Gaines, L., Kelly, J. C. & Wang, M. 2019*b* Everbatt: A closed-loop battery recycling cost and environmental impacts model. Report. Argonne National Lab.(ANL), Argonne, IL (United States).

Davidsson Kurland, S. 2019 Energy use for gwh-scale lithium-ion battery production. *Environmental Research Communications* **2** (1), 012001.

Degen, F. & Krätzig, O. 2021 Modelling large scale manufacturing of automotive battery cells – impact of new technologies on production economies. `http://dx.doi.org/10.2139/ssrn.4019171`, [Accessed June 1st, 2022].

Degen, F. & Schütte, M. 2022 Life cycle assessment of the energy consumption and ghg emissions of state-of-the-art automotive battery cell production. *Journal of Cleaner Production* **330**, 129798.

Deng, Y., Li, J., Li, T., Gao, X. & Yuan, C. 2017 Life cycle assessment of lithium sulfur battery for electric vehicles. *Journal of Power Sources* **343**, 284–295.

DERA 2021*a* Preismonitor november 2021. `https://www.deutsche-rohstoffagentur.de/DERA/DE/Produkte/Rohstoffpreise/Preismonitor/preismonitor_node.html`, [Accessed January 4th, 2022].

DERA 2021*b* Volatilitätsmonitor november 2021. `https://www.bgr.bund.de/EN/Themen/Min_rohstoffe/Produkte/produkte_node_en.html?tab=Commodity+prices`, [Accessed January 1st, 2022].

Dolega, P., Buchert, M. & Betz, J. 2020 Environmental and socio-economic challenges in battery supply chains: graphite and lithium. `https://www.oeko.de/fileadmin/oekodoc/Graphite-Lithium-Env-Soc-Eco-Challenges.pdf`, [Accessed January 2nd, 2022].

Duchin, F. & Levine, S. H. 2011 Sectors may use multiple technologies simultaneously: The rectangular choice-of-technology model with binding factor constraints. *Economic Systems Research* **23** (3), 281–302.

Duffner, F., Krätzig, O. & Leker, J. 2020*a* Battery plant location considering the balance between knowledge and cost: A comparative study of the eu-28 countries. *Journal of Cleaner Production* **264**, 121428.

Duffner, F., Mauler, L., Wentker, M., Leker, J. & Winter, M. 2021 Large-scale automotive battery cell manufacturing: Analyzing strategic and operational effects on manufacturing costs. *International Journal of Production Economics* **232**, 107982.

Duffner, F., Wentker, M., Greenwood, M. & Leker, J. 2020*b* Battery cost modeling: A review and directions for future research. *Renewable and Sustainable Energy Reviews* **127**, 109872.

Dunn, J. B., Gaines, L., Barnes, M., Sullivan, J. L. & Wang, M. 2014 Material and energy flows in the materials production, assembly, and end-of-life stages of the automotive

lithium-ion battery life cycle. https://greet.es.anl.gov/publication-li-ion, [Accessed January 1st, 2022].

Dunn, J. B., James, C., Gaines, L., Gallagher, K., Dai, Q. & Kelly, J. C. 2015 Material and energy flows in the production of cathode and anode materials for lithium ion batteries. https://greet.es.anl.gov/publication-anode-cathode-liion, [Accessed January 1st, 2022].

Dühnen, S., Betz, J., Kolek, M., Schmuch, R., Winter, M. & Placke, T. 2020 Toward green battery cells: Perspective on materials and technologies. *Small Methods* **4** (7), 2000039.

EC 2020*a* Batteries Europe, strategic research agenda for batteries. https://ec.europa.eu/energy/topics/technology-and-innovation/batteries-europe/news-articles-and-publications/sra_en, [Accessed January 1st, 2022].

EC 2020*b* Communication from the commission to the european parliament, the council, the european economic and social committee and the committee of the regions: Critical raw materials resilience: Charting a path towards greater security and sustainability. http://ec.europa.eu/growth/sectors/raw-materials/specific-interest/critical_nl, [Accessed January 1st, 2022].

EC 2021 *Proposal for a regulation of the European parliament and of the Council concerning batteries and waste batteries, repealing Directive 2006/66/EC and amending Regulation (EU) No 2019/1020. COM(2020) 798/3*. European Commision.

ECC 2021 European construction costs: cost index. http://constructioncosts.eu/cost-index/, [Accessed January 1st, 2022].

EEA 2021 EEA greenhouse gases - data viewer. https://www.eea.europa.eu/publications/data-and-maps/data/data-viewers/greenhouse-gases-viewer, [Accessed January 1st, 2022].

Ellingsen, L. A.-W., Majeau-Bettez, G., Singh, B., Srivastava, A. K., Valøen, L. O. & Strømman, A. H. 2014 Life cycle assessment of a lithium-ion battery vehicle pack. *Journal of Industrial Ecology* **18** (1), 113–124.

Ellingsen, L. A.-W., Singh, B. & Strømman, A. H. 2016 The size and range effect: lifecycle greenhouse gas emissions of electric vehicles. *Environmental Research Letters* **11** (5), 054010.

Engels, P., Cerdas, F., Dettmer, T., Frey, C., Hentschel, J., Herrmann, C., Mirfabrikikar, T. & Schueler, M. 2022 Life cycle assessment of natural graphite production for lithium-ion battery anodes based on industrial primary data. *Journal of Cleaner Production* **336**, 130474.

EPA 2021*a* Data on cars used for testing fuel economy. https://www.epa.gov/compliance-and-fuel-economy-data/data-cars-used-testing-fuel-economy, [Accessed January 1st, 2022].

EPA 2021*b* EPA's transportation and air quality document index system (dis). https://iaspub.epa.gov/otaqpub/, [Accessed January 1st, 2022].

Epp, A., Wendland, R., Behrendt, J., Gerlach, R. & Sauer, D. U. 2022 Holistic battery system design optimization for electric vehicles using a multiphysically coupled lithium-ion battery design tool. *Journal of Energy Storage* **52**, 104854.

Eurostat 2021*a* Electricity prices for non-household consumers - NRG_PC_205. https://ec.europa.eu/eurostat/databrowser/view/nrg_pc_205/default/table?lang=en, [Accessed January 1st, 2022].

Eurostat 2021*b* Gas prices for non-household consumers - NRG_PC_203. https://ec.europa.eu/eurostat/databrowser/view/nrg_pc_203/default/table?lang=en, [Accessed January 1st, 2022].

Eurostat 2021*c* Labour cost levels by NACE Rev. 2 activity (lc_lci_lev). https://ec.europa.eu/eurostat/databrowser/view/lc_lci_lev/default/table?lang=en, [Accessed January 2nd, 2022].

EV-Database 2021 All electric vehicles. https://ev-database.org, [Accessed January 1st, 2022].

Greenwood, M., Wentker, M. & Leker, J. 2021 A bottom-up performance and cost assessment of lithium-ion battery pouch cells utilizing nickel-rich cathode active materials and silicon-graphite composite anodes. *Journal of Power Sources Advances* **9**, 100055.

Günter, F. J. & Wassiliadis, N. 2022 State of the art of lithium-ion pouch cells in automotive applications: Cell teardown and characterization. *Journal of The Electrochemical Society* **169** (3), 030515.

Heil, G., Kormann, C. & Adel, J. 2003 Lithium oxide containing lithium intercalation compounds. *European Patent Application No. EP1204601, published on* **2**, 19.

Helbig, C., Bradshaw, A. M., Wietschel, L., Thorenz, A. & Tuma, A. 2018 Supply risks associated with lithium-ion battery materials. *Journal of Cleaner Production* **172**, 274–286.

IEA 2021 Global EV policy explorer. https://www.iea.org/articles/global-ev-policy-explorer, [Accessed January 1st, 2022].

IMF 2022 Primary commodity price system. https://www.imf.org/en/Research/commodity-prices, [Accessed January 4th, 2022].

ISO 2020 *ISO 14040:2006+A1:2020 Environmental management Life cycle assessment Principles and framework*. Geneva: International Organization for Standardization.

Jinasena, A., Burheim, O. S. & Strømman, A. H. 2021 A flexible model for benchmarking the energy usage of automotive lithium-ion battery cell manufacturing. *Batteries* **7** (1), 14.

Kallitsis, E., Korre, A., Kelsall, G., Kupfersberger, M. & Nie, Z. 2020 Environmental life cycle assessment of the production in china of lithium-ion batteries with nickel-cobalt-manganese cathodes utilising novel electrode chemistries. *Journal of Cleaner Production* **254**, 120067.

Kelly, J. C., Wang, M., Dai, Q. & Winjobi, O. 2021 Energy, greenhouse gas, and water life cycle analysis of lithium carbonate and lithium hydroxide monohydrate from brine and ore resources and their use in lithium ion battery cathodes and lithium ion batteries. *Resources, Conservation and Recycling* **174**.

Kim, H. C. & Wallington, T. J. 2016 Life cycle assessment of vehicle lightweighting: A physics-based model to estimate use-phase fuel consumption of electrified vehicles. *Environ Sci Technol* **50** (20), 11226–11233.

Kim, H. C., Wallington, T. J., Arsenault, R., Bae, C., Ahn, S. & Lee, J. 2016 Cradle-to-gate emissions from a commercial electric vehicle li-ion battery: A comparative analysis. *Environ Sci Technol* **50** (14), 7715–22.

Knehr, K. W., Kubal, J. J., Nelson, P. A. & Ahmed, S. 2022 Battery performance and cost modeling for electric-drive vehicles: A manual for batpac v5.0.

Kwade, A., Haselrieder, W., Leithoff, R., Modlinger, A., Dietrich, F. & Droeder, K. 2018 Current status and challenges for automotive battery production technologies. *Nature Energy* **3** (4), 290–300.

Lain, M. J., Brandon, J. & Kendrick, E. 2019 Design strategies for high power vs. high energy lithium ion cells. *Batteries* **5** (4), 64.

Lamb, W. F., Grubb, M., Diluiso, F. & Minx, J. C. 2021 Countries with sustained greenhouse gas emissions reductions: an analysis of trends and progress by sector. *Climate Policy* **22** (1), 1–17.

Lambert, F. 2021 Tesla gives rare update on 4680 battery cell production: still some work to do. `https://electrek.co/2021/07/26/tesla-update-4680-battery-cell-production/`, [Accessed January 1st, 2022].

Lee, J.-W., Kim, J.-I. & Roh, K. C. 2012 Lithium manganese oxide with excellent electrochemical performance prepared from chemical manganese dioxide for lithium ion batteries. *Solid State Sciences* **14** (9), 1251–1255.

Li, J., Fleetwood, J., Hawley, W. B. & Kays, W. 2022 From materials to cell: State-of-the-art and prospective technologies for lithium-ion battery electrode processing. *Chem Rev* **122** (1), 903–956.

Liu, Z., Yu, A. & Lee, J. Y. 1999 Synthesis and characterization of lini1xycoxmnyo2 as the cathode materials of secondary lithium batteries. *Journal of Power Sources* **81-82**, 416–419.

Liu, Z., Yu, Q., Zhao, Y., He, R., Xu, M., Feng, S., Li, S., Zhou, L. & Mai, L. 2019 Silicon oxides: a promising family of anode materials for lithium-ion batteries. *Chem Soc Rev* **48** (1), 285–309.

LME 2022 Reports by metal. https://www.lme.com/Market-Data/Reports-and-data/Reports-by-metal, [Accessed January 4th, 2022].

Majeau-Bettez, G., Hawkins, T. R. & Stromman, A. H. 2011*a* Life cycle environmental assessment of lithium-ion and nickel metal hydride batteries for plug-in hybrid and battery electric vehicles. *Environ Sci Technol* **45** (10), 4548–54.

Majeau-Bettez, G., Stromman, A. H. & Hertwich, E. G. 2011*b* Evaluation of process- and input-output-based life cycle inventory data with regard to truncation and aggregation issues. *Environ Sci Technol* **45** (23), 10170–7.

Manjong, N. B., Usai, L., Burheim, O. S. & Strømman, A. H. 2021 Life cycle modelling of extraction and processing of battery minerals—a parametric approach. *Batteries* **7** (3), 57.

MARIAN 2019 Four insulating materials for use in ev batteries. https://blog.marianinc.com/blog/4-insulating-materials-for-use-in-ev-batery, [Accessed January 1st, 2022].

Mauler, L., Lou, X., Duffner, F. & Leker, J. 2022 Technological innovation vs. tightening raw material markets: Falling battery costs put at risk. *Energy Advances* .

McKinsey 2021 Building better batteries: Insights on chemistry and design from china. https://www.mckinsey.com/industries/automotive-and-assembly/our-insights/building-better-batteries-insights-on-chemistry-and-design-from-china, [Accessed January 1st, 2022].

Melin, H. E., Rajaeifar, M. A., Ku, A. Y., Kendall, A., Harper, G. & Heidrich, O. 2021 Global implications of the eu battery regulation. *Science* **373** (6553), 384–387.

Nelson, P., Gallagher, K., Bloom, I. & Dees, D. 2012 Modeling the performance and cost of lithium-ion batteries for electric-drive vehicles - second edition. https://www.osti.gov/biblio/1209682-modeling-performance-cost-lithium-ion-batteries-electric-drive-vehicles-second-edition, [Accessed January 4th, 2022].

Nelson, P., Gallagher, K., Bloom, I. & Dees, D. 2019 Modeling the performance and cost of lithium-ion batteries for electric-drive vehicles - third edition. https://publications.anl.gov/anlpubs/2011/10/71302.pdf, [Accessed January 1st, 2022].

Ngala, J. K., Chernova, N. A., Ma, M., Mamak, M., Zavalij, P. Y. & Whittingham, M. S. 2004 The synthesis, characterization and electrochemical behavior of the layered lini0.4mn0.4co0.2o2 compound. *Journal of Materials Chemistry* **14** (2), 214–220.

Notter, D. A., Gauch, M., Widmer, R., Wäger, P., Stamp, A., Zah, R. & Althaus, H.-J. 2010 Contribution of li-ion batteries to the environmental impact of electric vehicles. *Environmental Science & Technology* **44** (17), 6550–6556.

OECD 2022 Inflation (cpi). https://data.oecd.org/price/inflation-cpi.htm, [Accessed January 4th, 2022].

Olivetti, E. A. & Cullen, J. M. 2018 Toward a sustainable materials system. *Science* **360** (6396), 1396–1398.

Pell, R. & Tijsseling, L. 2020 First cobalt refinery - life cycle assessment. "https://www.firstcobalt.com/_resources/reports/First-Cobalt-LCA-Final-Report.pdf?v=0.477", [Accessed January 1st, 2022].

Peters, J., Buchholz, D., Passerini, S. & Weil, M. 2016 Life cycle assessment of sodium-ion batteries. *Energy Environmental Science* **9** (5), 1744–1751.

Peters, J. F., Baumann, M., Zimmermann, B., Braun, J. & Weil, M. 2017 The environmental impact of li-ion batteries and the role of key parameters – a review. *Renewable and Sustainable Energy Reviews* **67**, 491–506.

Peters, J. F. & Weil, M. 2018 Providing a common base for life cycle assessments of li-ion batteries. *Journal of Cleaner Production* **171**, 704–713.

Peters, M., Timmerhaus, K. & West, R. 2003 *Plant Design and Economics for Chemical Engineers*. New York: McGraw-Hill Education.

Pettinger, K.-H. & Dong, W. 2016 When does the operation of a battery become environmentally positive? *Journal of The Electrochemical Society* **164** (1), A6274–A6277.

Pettinger, K.-H., Kampker, A., Hohenthanner, C.-R., Deutskens, C., Heimes, H. & vom Hemdt, A. 2018 Lithium-ion cell and battery production processes. In *Lithium-Ion Batteries: Basics and Applications* (ed. R. Korthauer), pp. 211–226. Berlin, Heidelberg: Springer.

Philippot, M., Alvarez, G., Ayerbe, E., Van Mierlo, J. & Messagie, M. 2019 Eco-efficiency of a lithium-ion battery for electric vehicles: Influence of manufacturing country and commodity prices on ghg emissions and costs. *Batteries* **5** (1), 23.

Quinn, J. B., Waldmann, T., Richter, K., Kasper, M. & Wohlfahrt-Mehrens, M. 2018 Energy density of cylindrical li-ion cells: A comparison of commercial 18650 to the 21700 cells. *Journal of The Electrochemical Society* **165** (14), A3284–A3291.

Reuters 2021 Lithium price surge could charge demand for lead in batteries. https://www.reuters.com/markets/commodities/lithium-price-surge-could-charge-demand-lead-batteries-2021-12-16/, [Accessed January 1st, 2022].

Roskill 2020 https://roskill.com/news/silicon-graphite-mou-for-lithium-ion-battery-raw-material-supply/, [Accessed January 1st, 2022].

Sacchi, R., Bauer, C., Cox, B. & Mutel, C. 2022 When, where and how can the electrification of passenger cars reduce greenhouse gas emissions? *Renewable and Sustainable Energy Reviews* **162**, 112475.

Safoutin, M., McDonald, J. & Ellies, B. 2018 Predicting the future manufacturing cost of batteries for plug-in vehicles for the u.s. environmental protection agency (epa) 2017–2025 light-duty greenhouse gas standards. *World Electric Vehicle Journal* **9** (3), 42.

Sankaran, G. & Venkatesan, S. 2021 Standardization of electric vehicle battery pack geometry form factors for passenger car segments in india. *Journal of Power Sources* **502**, 230008.

Satyavani, T. V. S. L., Srinivas Kumar, A. & Subba Rao, P. S. V. 2016 Methods of synthesis and performance improvement of lithium iron phosphate for high rate li-ion batteries: A review. *Engineering Science and Technology, an International Journal* **19** (1), 178–188.

Schmuch, R., Wagner, R., Hörpel, G., Placke, T. & Winter, M. 2018 Performance and cost of materials for lithium-based rechargeable automotive batteries. *Nature Energy* **3** (4), 267–278.

Schnell, J., Knörzer, H., Imbsweiler, A. J. & Reinhart, G. 2020 Solid versus liquid—a bottom-up calculation model to analyze the manufacturing cost of future high-energy batteries. *Energy Technology* **8** (3), 1901237.

Shaffer, B., Auffhammer, M. & Samaras, C. 2021 Make electric vehicles lighter to maximize climate and safety benefits. *Nature* **598**, 254–256.

SMM 2022 Shanghai metals market - new energy. https://www.metal.com/, [Accessed Mai 13th, 2022].

Statista 2022 Graphite prices worldwide from 2011 to 2020, by flake grade. https://www.statista.com/statistics/452304/graphite-prices-worldwide-prediction-by-flake-grade/, [Accessed January 1st, 2022].

Stefanova, M., Tripepi, C., Zamagni, A. & Masoni, P. 2014 Goal and scope in life cycle sustainability analysis: The case of hydrogen production from biomass. *Sustainability* **6** (8), 5463–5475.

Sun, X., Luo, X., Zhang, Z., Meng, F. & Yang, J. 2020 Life cycle assessment of lithium nickel cobalt manganese oxide (NCM) batteries for electric passenger vehicles. *Journal of Cleaner Production* **273**, 123006.

Surovtseva, D., Crossin, E., Pell, R. & Stamford, L. 2022 Toward a life cycle inventory for graphite production. *Journal of Industrial Ecology* **n/a** (n/a), in press.

Susarla, N. & Ahmed, S. 2020 Estimating the cost and energy demand of producing lithium manganese oxide for li-ion batteries. Report. Argonne National Lab.(ANL), Argonne, IL (United States).

Terrafame 2020 Terrafame's nickel sulphate production offers the lowest carbon footprint in the industry - altogether 60% lower than in existing conventional processes. https://www.terrafame.com/media/terrafame-ltd-carbon-footprint.pdf.

Townsend, T. . 2020 International construction market survey. https://www.turnerandtownsend.com/en/perspectives/international-construction-market-survey-2019/, [Accessed January 1st, 2022].

USGS 2017 Graphite [advance release]. In *Minerals Yearbook* (ed. USGS), pp. 32.1 – 32.14. Reston: U.S. Geological Survey.

USGS 2021*a* Mineral commodity summaries. https://www.usgs.gov/centers/national-minerals-information-center/mineral-commodity-summaries, [Accessed January 4th, 2022].

USGS 2021*b* Minerals yearbook [tables only]. https://www.usgs.gov/centers/national-minerals-information-center/manganese-statistics-and-information, [Accessed January 4th, 2022].

Volkswagen 2021 2021 volkswagen id.4 technical specifications. Report. [Accessed January 1st, 2022].

Wells, P. 2018 Degrowth and techno-business model innovation: The case of riversimple. *Journal of Cleaner Production* **197**, 1704–1710.

Wentker, M., Greenwood, M. & Leker, J. 2019 A bottom-up approach to lithium-ion battery cost modeling with a focus on cathode active materials. *Energies* **12** (3).

Winjobi, O., Dai, Q. & Kelly, J. C. 2020 Update of bill-of-materials and cathode chemistry addition for lithium-ion batteries in greet 2020. https://greet.es.anl.gov/publication-li_update_2020, [Accessed January 1st, 2022].

Wolfram, P., Tu, Q., Heeren, N., Pauliuk, S. & Hertwich, E. G. 2021 Material efficiency and climate change mitigation of passenger vehicles. *Journal of Industrial Ecology* **25** (2), 494–510.

Wood, D. L., Li, J. & Daniel, C. 2015 Prospects for reducing the processing cost of lithium ion batteries. *Journal of Power Sources* **275**, 234–242.

World Bank 2022 World bank commodity price data - annual prices, 1960 to present, real 2010 us dollars. https://www.worldbank.org/en/research/commodity-markets, [Accessed January 4th, 2022].

Yang, C., Tong, H., Luo, C., Yuan, S., Chen, G. & Yang, Y. 2017 Boehmite particle coating modified microporous polyethylene membrane: A promising separator for lithium ion batteries. *Journal of Power Sources* **348**, 80–86.

Yuan, C., Deng, Y., Li, T. & Yang, F. 2017 Manufacturing energy analysis of lithium ion battery pack for electric vehicles. *CIRP Annals* **66** (1), 53–56.

Zackrisson, M., Avellán, L. & Orlenius, J. 2010 Life cycle assessment of lithium-ion batteries for plug-in hybrid electric vehicles – critical issues. *Journal of Cleaner Production* **18** (15), 1519–1529.

Zampori, L. & Pant, R. 2019 Suggestions for updating the product environmental footprint (pef) method. *Publications Office of the European Union: Luxembourg* .

Zhang, S. S. 2006 A review on electrolyte additives for lithium-ion batteries. *Journal of Power Sources* **162** (2), 1379–1394.

Zhang, X., Wang, D., Qiu, X., Ma, Y., Kong, D., Mullen, K., Li, X. & Zhi, L. 2020 Stable high-capacity and high-rate silicon-based lithium battery anodes upon two-dimensional covalent encapsulation. *Nat Commun* **11** (1), 3826.

Zhu, P., Gastol, D., Marshall, J., Sommerville, R., Goodship, V. & Kendrick, E. 2021 A review of current collectors for lithium-ion batteries. *Journal of Power Sources* **485**, 229321.
